# Supplementary material for: Telomere damage induces internal loops that generate telomeric circles
Source: Nat Commun. 2020 Oct 20;11:5297. doi: 10.1038/s41467-020-19139-4 (PMC7576219; doi:10.1038/s41467-020-19139-4)
Supplement: Supplementary file 1 — Supplementary Information [file 41467_2020_19139_MOESM1_ESM.pdf]

Supplementary Information

Telomere damage induces internal loops that generate telomeric circles

Mazzucco et al.

Supplementary Figure 1

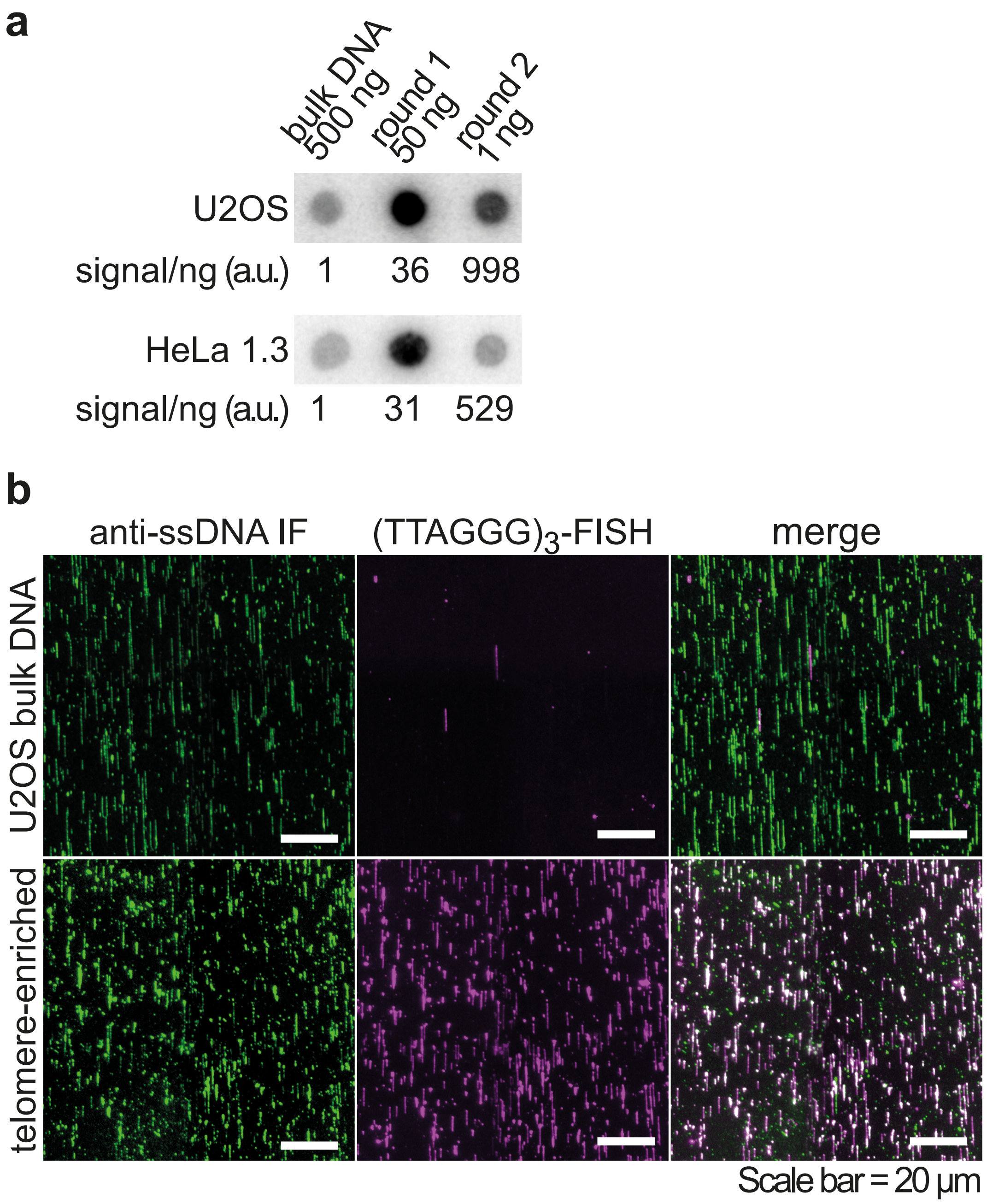

Supplementary Figure 1. Enrichment of telomeric repeats from human cells (related to Figure 1).

a. Dot blot analysis showing the enrichment of the telomeric repeats. Genomic DNA prepared from U2OS cells and HeLa 1.3 cells with long telomeres, was subjected to the telomere enrichment procedure (described in Figure 1). The indicated amounts from each enrichment step were spotted on a membrane and hybridized with a probe recognizing the TTAGGG repeats. The amount of signal/ng is reported relative to the non-enriched DNA. Source data are provid ed as a Source Data File.

b. Single molecule analysis showing telomere enrichment from U2OS cells. Enriched telomeric DNA was combed on silanized coverslips, denatured in situ and labeled sequentially with an antibody against single-stranded DNA and a Cy3-labeled (TTAGGG)3 PNA probe.

Supplementary Figure 2

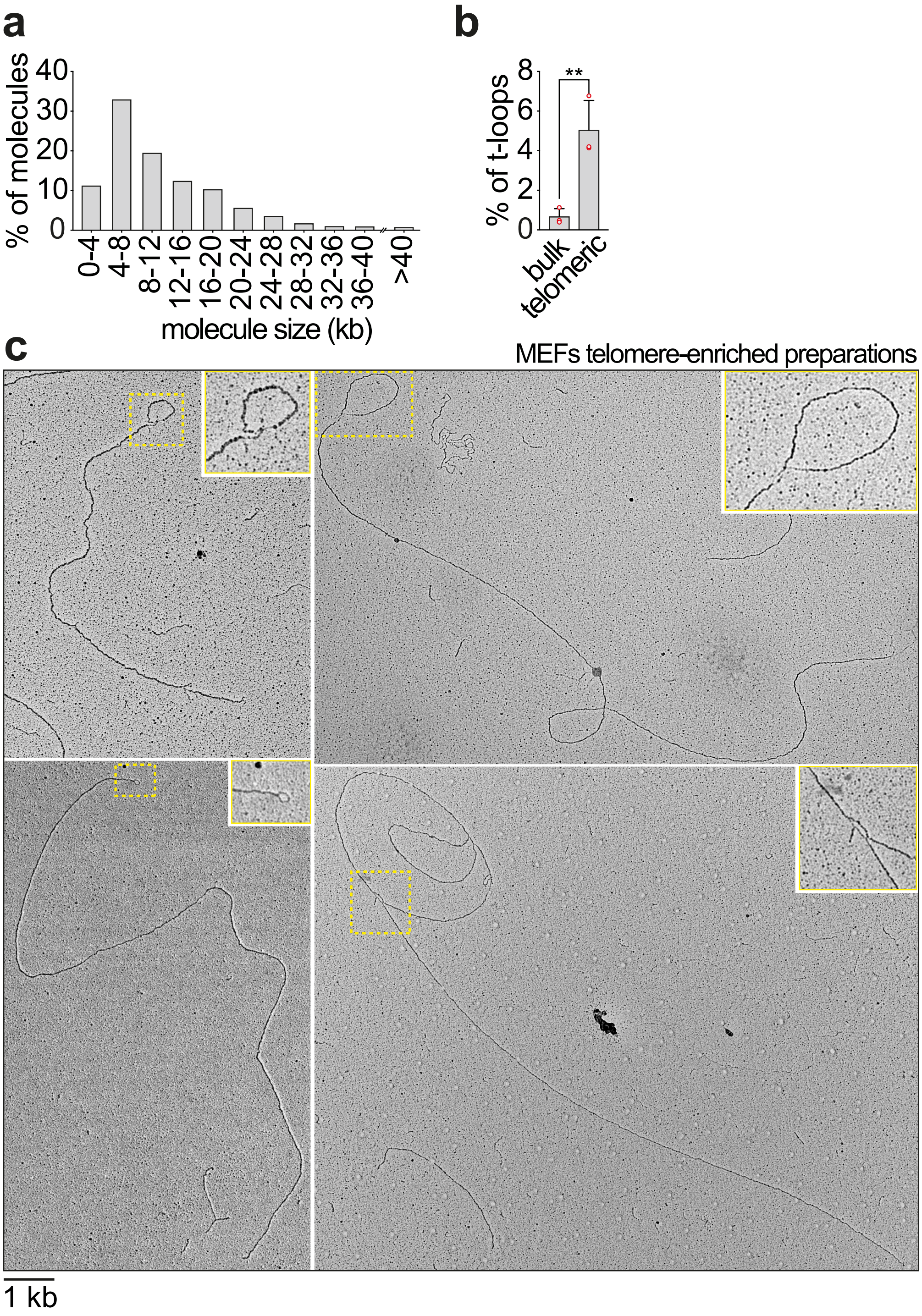

Supplementary Figure 2. Occurrence of t-loops in the telomere-enriched samples (related to Figure 2).

a. Molecule length distribution from the experiments with telomere-enriched DNA, described in Figure 2. N=1516 molecules. Source data are provided as a Source Data File.

b. Frequency of t-loops in the telomere-enriched (telomeric) and non-enriched genomic DNA (bulk) samples from the experiments described in Figure 2. Bars represent the mean with the standard deviation. Single data points are also shown as red dots. P value = 0.0082, was derived from unpaired, two-tailed, Student's t-test. Source data are provided as a Source Data File.

c. Examples of molecules with t-loops observed in the telomere-enriched samples. Insets show 2X enlargements of the area inside the yellow rectangles.

Supplementary Figure 3

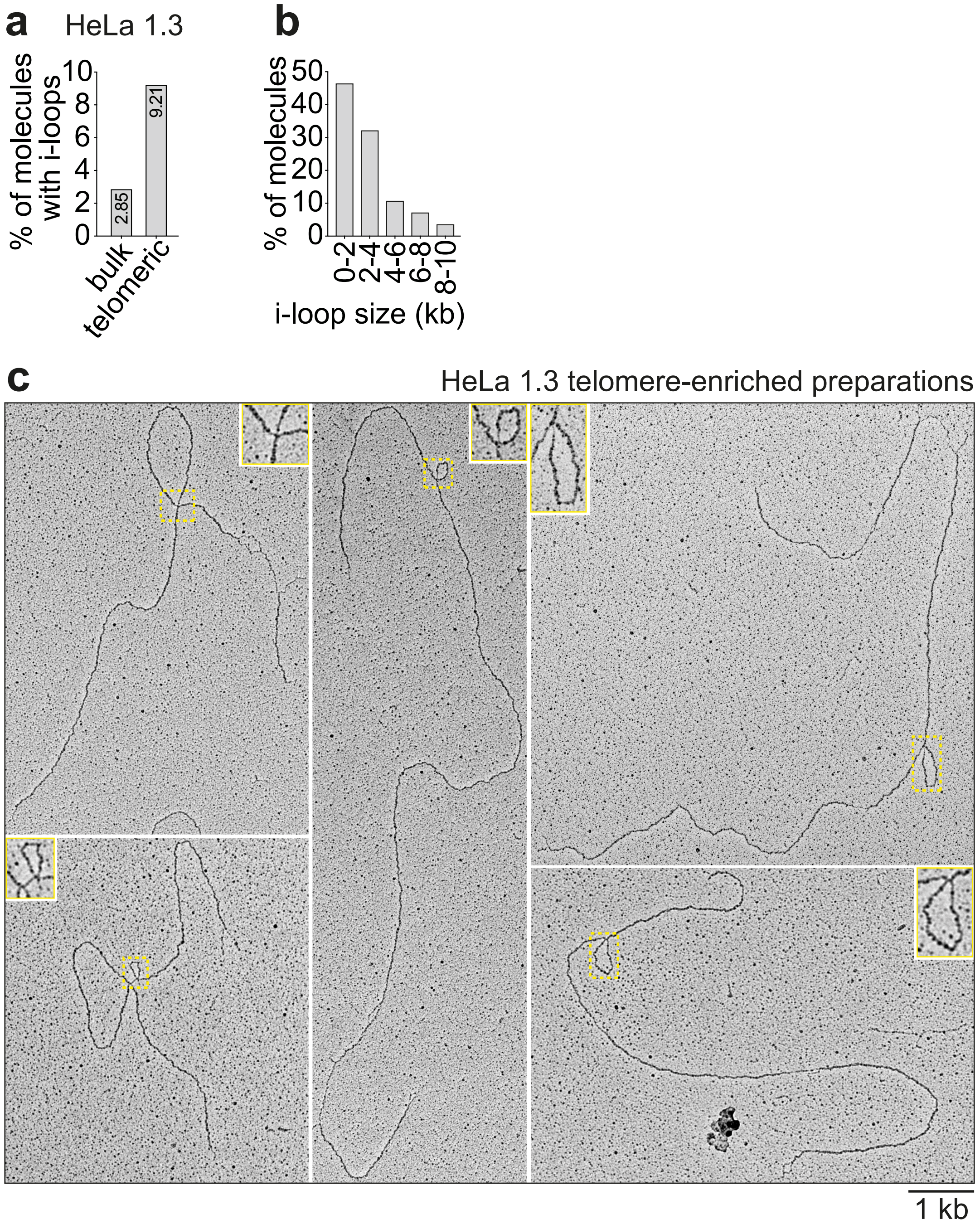

Supplementary Figure 3. Accumulation of i-loops at human telomeres (related to Figure 2).

a. Quantification of i-loop occurrence in telomere-enriched (telomeric) and non-enriched genomic DNA (bulk) from HeLa 1.3 cells with long telomeres. The percentage of molecules with i-loops is reported for each sample. Telomere enriched N=239, non-enriched N=1510 molecules.

b. I-loop size distribution, from the experiment described in (A). N=28 i-loops.

c. Examples of molecules with i-loops observed in the telomere-enriched sample from HeLa 1.3 cells. Insets show 2X enlargements of the area inside the yellow rectangles. Source data are provided as a Source Data File.

Supplementary Figure 4

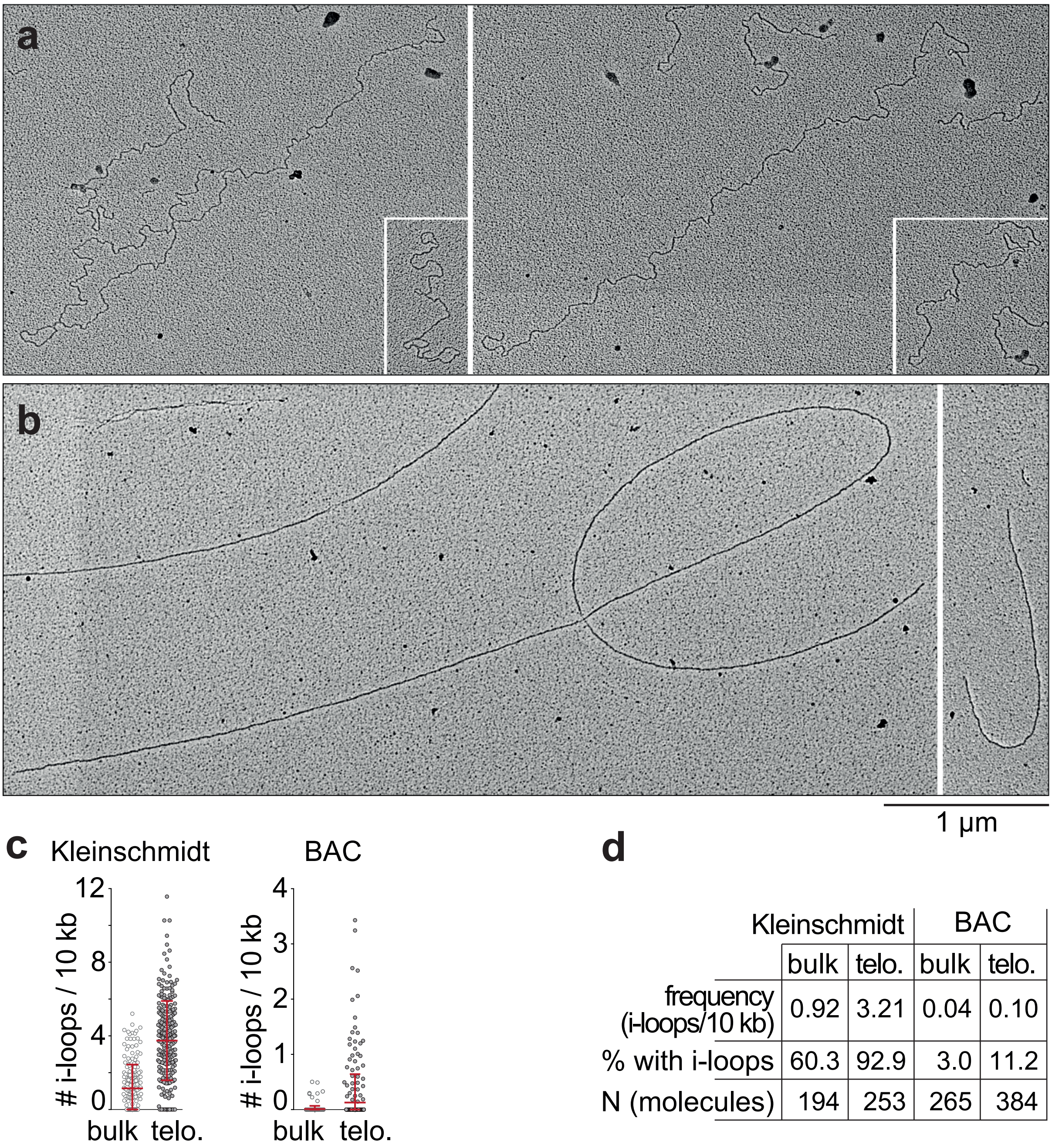

d

	Kleinschmidt		BAC	
	bulk	telo.	bulk	telo.
frequency (i-loops/10 kb)	0.92	3.21	0.04	0.10
% with i-loops	60.3	92.9	3.0	11.2
N (molecules)	194	253	265	384

Supplementary Figure 4. Accumulation of i-loops at telomeric repeats, seen both in Kleinschmidt and BAC EM spreads (related to Figure 2).

a, b. KpnI-digested mouse DNA spread either with the Kleinschmidt method (a) or with the BAC method (b). Large, overlapping field were acquired in EM and stitched with the Gatan Digital Micrograph software. Scale bar 1 µm.

c. I-loop density per molecule (i.e. number of i-loops/10 kb) in the molecules analyzed for each of the indicated samples Each dot represents a molecule and bars represent mean with SD. In table (d) is reported the number of molecules analysed for each sample. Source data are provided as a Source Data File.

d. Table comparing the i-loop occurrence in BAC vs Kleinschmidt spreads. The same DNA preparations were spread in parallel with the two methods and analyzed in EM. The first line reports the mean i-loop frequency (i.e. the cumulative number of i-loops encountered/cumulative molecule length) for each sample. The second line reports the percentage of the molecules with one or more i-loops, in the indicated samples. The third line represents the number of molecules analyzed for each sample.

Supplementary Figure 5

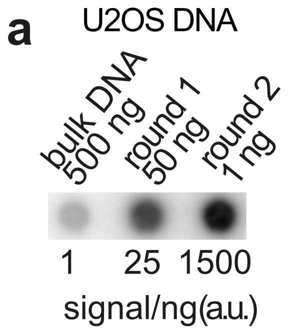

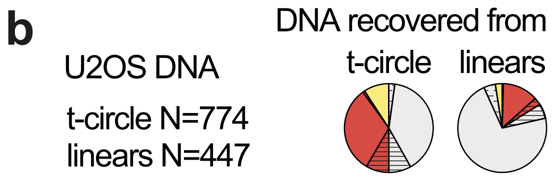

	t-circle	linears
**circles**	**9.2%**	**2.5%**
**with i-loops**	**40.8%**	**16.1%**
t-loops with i.loops	8.4%	2.7%
branched with i.loops	0.8%	0.4%
linear with i.loops	31.6%	13.0%
**without i-loops**	**50.0%**	**81.4%**
t-loops	8.4%	5.4%
branched	2.2%	4.2%
linear	39.4%	71.8%

Supplementary Figure 5. I-loops in the t-circle arc of ALT cells (related to Figure 4).
a. Dot blot showing the U2OS telomere enrichment for the experiment shown in Figure 4a. The indicated amounts from each enrichment step were spotted on a membrane and hybridized with a probe recognizing the TTAGGG repeats. In the second round, 1 ng of DNA, recovered from the linear signal, was used to verify the enrichment. The amount of signal/ng is reported relative to the non-enriched DNA. Source data are provided as a Source Data File.
b. This is the same pie chart shown in Figure 4b, that includes the detailed distribution of the molecules recovered from the 2D-gel. Note that i-loops occur also in molecules having a t-loop at the end, or at branched molecules (Y-shaped or X-shaped).

Supplementary Figure 6

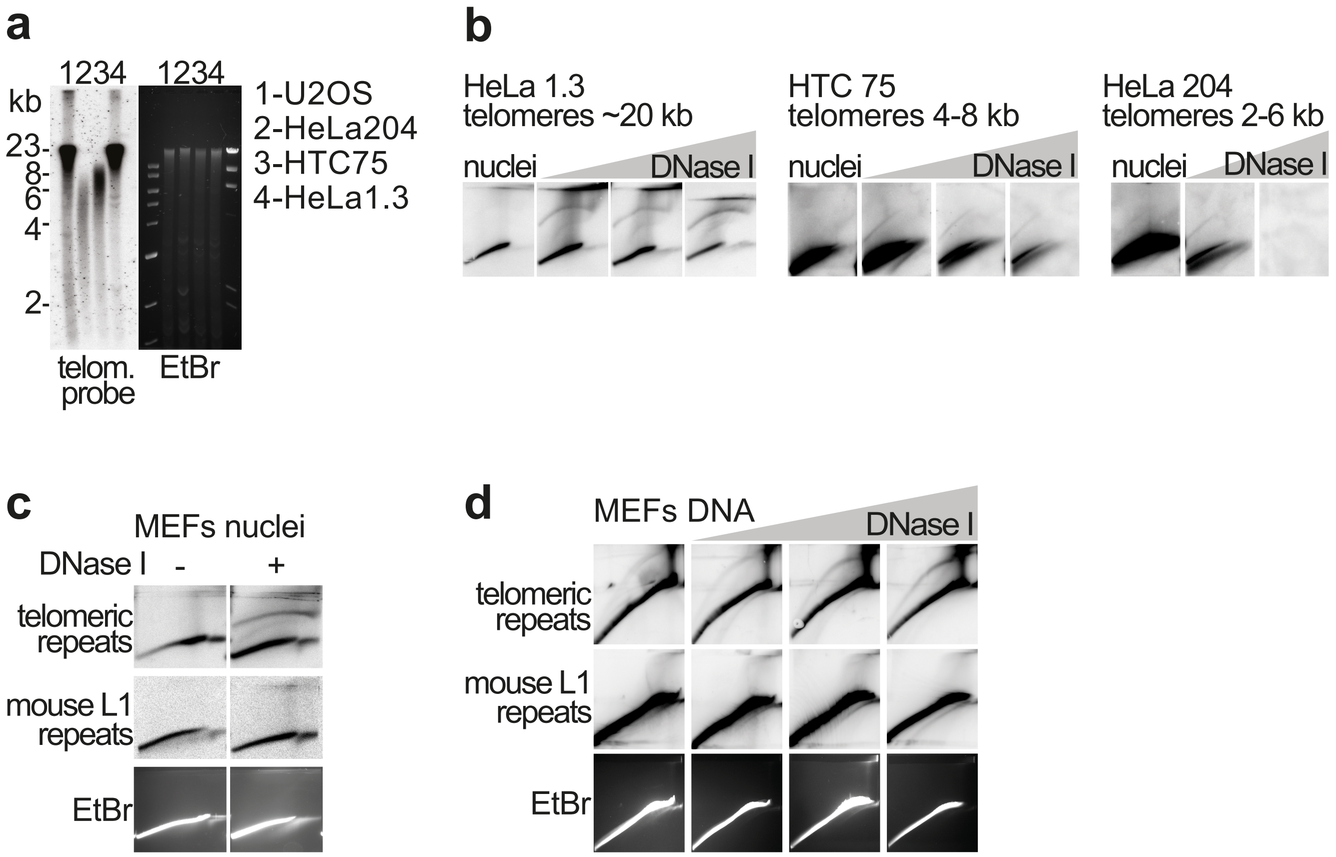

Supplementary Figure 6. I-loops are induced by single-strand damage at telomeric repeats (related to Figure 5).

a. Blot of AluI- and MboI-digested DNA showing telomere lengths for the indicated cell lines. Source data are provided as a Source Data File.

b. Induction of the t-circle arc by nicks and gaps at human telomeres and its dependence on telomere length. HeLa 1.3 nuclei (with telomeres around, ~20 kb in average) were incubated with 0; 0.5; 1 or 2.5 µg/ml of DNase I for 8 minutes at RT. HTC75 nuclei (with telomeres around 4-8 kb) and HeLa 204 nuclei (with telomeres around, 2-6 kb) were incubated with 0; 5; 10 or 20 µg/ml of DNase I for 8 minutes at RT. The nuclei were then processed for 2D-gel analysis, as described in Figure 5a. Source data are provided as a Source Data File.

c. DNase I treatment does not induce the t-circle arc in the bulk genomic DNA or at the mouse L1 repeats. In a similar experiment as the one described in Figure 5A, nuclei were treated with 2.5 µg/ml of DNase I. DNA was digested with BglI, split in two and separated on 2D-gels, in duplicate. After blotting, one membrane was hybridized with a telomeric probe, while the other with a probe recognizing the mouse L1 repeats. The EtBr staining of one of the second-dimension gels is shown at the bottom. Source data are provided as a Source Data File.

d. DNase I treatment on isolated DNA does not induce the t-circle arc in the bulk genomic DNA or at the L1 repeats. DNA from the same experiment shown in Figure 5B, was digested with KpnI, split in two and then separated in 2D-gels, in duplicate. After blotting, one membrane was hybridized with a probe recognizing the TTAGGG repeats and the other with a probe recognizing the mouse L1 repeats. The EtBr staining of one of the second-dimension gels is shown at the bottom. Source data are provided as a Source Data File.
