# Supplementary material for: Telomere damage induces internal loops that generate telomeric circles
Source: Nat Commun. 2020 Oct 20;11:5297. doi: 10.1038/s41467-020-19139-4 (PMC7576219; doi:10.1038/s41467-020-19139-4)
Supplement: Supplementary file 3 — Description of Additional Supplementary Files [file 41467_2020_19139_MOESM3_ESM.pdf]

Description of Additional Supplementary Files

File Name: Supplementary Software 1
Description: The macro is written in IJ macro language, by Paolo Maiuri at IFOM. It can be opened by dragging and dropping the file in the FIJI/Image J bar.

Operations:

Once provided with a folder with EM images the macro opens them in sequential order and asks the user to define which areas of an image she wants to analyze.

-duplicates the user-selected areas in a new window so that the user can analyze it and stores the coordinates (ROIs).

-after the user has finished analyzing an area, the macro presents a prompt window where the user can enter the different parameters analyzed in user-defined fields (e.g. the molecule has an i-loop or is linear, length of the molecule = 5 µm; length of the i-loop is 0.2 µm).

-the macro then stores an annotated copy of the area analyzed and appends relevant values, entered by the user, to a text file for further analysis.

This automation saves time during the analysis of EM images and allows for a more consistent annotation and storage of data across multiple experiments.
