# Supplementary material for: Telomere damage induces internal loops that generate telomeric circles
Source: Nat Commun. 2020 Oct 20;11:5297. doi: 10.1038/s41467-020-19139-4 (PMC7576219; doi:10.1038/s41467-020-19139-4)
Supplement: Supplementary file 4 — Supplementary Software 1 [file 41467_2020_19139_MOESM4_ESM.zip › Supplementary Software File.rtf]

//This Macro was written by Paolo Maiuri at IFOM, The FIRC Institute of Molecular Onclolgy, Milan//setBatchMode(true);filesuffix = ".dm3";testRoisExist = false; //if you want to load existing ROIs, you have to change this statementto "true" and then leave only the ROI folder in the "destination folder" waitForUser("Have you changed the extension of your files to .dm3?");redo=true;dirX = getDirectory("Choose Source Directory "); starti=0;if(File.exists(dirX+"/TEMP_MacroYlli.txt")==0)	File.append("Temp rescue file", dirX+"/TEMP_MacroYlli.txt");else{	start_str = File.openAsString(dirX+"/TEMP_MacroYlli.txt");	ArrayLines=split(start_str,"\n");	starti_str=ArrayLines[lengthOf(ArrayLines)-1];	Dialog.create("Re-Start analysis");	Dialog.addCheckbox("Go to the next?", redo);	Dialog.show();	redo = Dialog.getCheckbox();	if(redo) starti = 1+parseInt(starti_str);	else  starti = parseInt(starti_str);};	listX = getFileList(dirX); if(starti != 0) print("Image to be analyzed: "+listX[starti]);nlistX =lengthOf(listX);dirOUT0 = getDirectory("Choose Output Directory "); dirOUT = dirOUT0+"Linears/";dirIntLoop = dirOUT0+"IntLoop/";dirLoops = dirOUT0+"Tloops/";dirCircle = dirOUT0+"Circle/";dirXAsymmetric = dirOUT0+"XAsymmetric/";dirXSymmetric = dirOUT0+"XSymmetric/";dirYAsymmetric = dirOUT0+"YAsymmetric/";dirYSymmetric = dirOUT0+"YSymmetric/";dirOthers = dirOUT0+"Others/";dirROI = dirOUT0+"ROI/";if(File.exists(dirOUT)==0)	File.makeDirectory(dirOUT);if(File.exists(dirLoops)==0)	File.makeDirectory(dirLoops);if(File.exists(dirIntLoop)==0)	File.makeDirectory(dirIntLoop);if(File.exists(dirCircle)==0)	File.makeDirectory(dirCircle);if(File.exists(dirXAsymmetric)==0)	File.makeDirectory(dirXAsymmetric);if(File.exists(dirXSymmetric)==0)	File.makeDirectory(dirXSymmetric);if(File.exists(dirYAsymmetric)==0)	File.makeDirectory(dirYAsymmetric);if(File.exists(dirYSymmetric)==0)	File.makeDirectory(dirYSymmetric);if(File.exists(dirOthers)==0)	File.makeDirectory(dirOthers);if(File.exists(dirROI)==0)	File.makeDirectory(dirROI);PathSummaryFile = dirOUT0+"Summary.csv";VariablesString = "TItle\tLength\tIntLoop\tXAsym\tXSym\tYAsym\tYSym\tTloop\tCircle\tOther\tLengthGap1\tLengthGap2\tLengthGap3\tLengthGap4";if (File.exists(PathSummaryFile)==false){	File.append(VariablesString, PathSummaryFile);}for (i=starti; i<nlistX; i++){	File.append(i, dirX+"/TEMP_MacroYlli.txt");	showProgress(i/nlistX);	PathToOpen = dirX+listX[i];	open(PathToOpen);	//run("Set... ", "zoom=40");	imageID0 =  getImageID();	title0 = getTitle();		if(endsWith(title0, filesuffix))		titleROI = replace(title0, filesuffix, "_ROIs.zip");	else if(endsWith(title0, ".tif"))		titleROI = replace(title0, ".tif", "_ROIs.zip");	print(titleROI);	roiManager("reset")	if(testRoisExist==false || File.exists(dirROI+titleROI)==false){		setTool("rectangle");		waitForUser("Find your molecules", "Select molecules you want to analyze\nDraw a rectangular selection & press 't'");}	else		roiManager("Open", dirROI+titleROI);	nrois = roiManager("count");		for(j=0; j<nrois; ++j)						//added by Ylli: This part renames ROIs in consecutive numbers, making it easier to identify single ROIs when needed	{												//added by Ylli		roiManager("select", j);					//added by Ylli		roiManager("rename", j);					//added by Ylli	}												//added by Ylli	roiManager("save", dirROI+titleROI);		for(j=0; j<nrois; j++)	{		roiManager("select", j);		run("Duplicate...", " ");		titlej = replace(title0, filesuffix, "_"+j+filesuffix );		rename(titlej);		setTool("freeline");		run("Clear Results");		waitForUser("Measure molecules", "Measure all path\n press 'm'");		//selectWindow("Results");		Dialog.create("Paths descriptor");		Dialog.addNumber("L:", 0);		Dialog.addNumber("IntLoop:", 0);		Dialog.addNumber("XAsym:", 0);		Dialog.addNumber("XSym:", 0);		Dialog.addNumber("YAsym:", 0);		Dialog.addNumber("YSym:", 0);		Dialog.addNumber("tLoop:", 0);		Dialog.addNumber("Circle:", 0);		Dialog.addNumber("Other:", 0);		Dialog.addNumber("gap1?:", 0);		Dialog.addNumber("gap2?:", 0);		Dialog.addNumber("gap3?:", 0);		Dialog.addNumber("gap4?:", 0);		Dialog.show();		L = Dialog.getNumber();		IntLoop = Dialog.getNumber();		XAsym = Dialog.getNumber();		XSym = Dialog.getNumber();		YAsym = Dialog.getNumber();		YSym = Dialog.getNumber();		tLoop =  Dialog.getNumber();		Circle =  Dialog.getNumber();		Other =  Dialog.getNumber();		g1 =  Dialog.getNumber();		g2 =  Dialog.getNumber();		g3 =  Dialog.getNumber();		g4 =  Dialog.getNumber();			VariablesString = titlej+"\t"+L+"\t"+IntLoop+"\t"+XAsym+"\t"+XSym+"\t"+YAsym+"\t"+YSym+"\t"+tLoop+"\t"+Circle+"\t"+Other+"\t"+g1+"\t"+g2+"\t"+g3+"\t"+g4;		//print(VariablesString);		File.append(VariablesString, PathSummaryFile);		getDimensions(width, height, channels, slices, frames);		fsize = 0.03*maxOf(width, height);					//the scale bar is set to 0.36 microns which corresponds to 1kb. If the images are acquired at a higher resolution, the scale bar will be given in nm and this value will need to be corrected. 		run("Scale Bar...", "width=0.36 height=8 font="+fsize+" color=Red background=None location=[Lower Right] overlay");  						setColor("blue");		setFont("SansSerif", fsize, "plain");		Overlay.drawString("L = "+L, 0.05*width, 0.1*height, 0.0);		Overlay.drawString(titlej, 0.05*width, 0.05*height, 0.0);		//this part of the code writes the values of the branches on the saved image. It has been commented because this macro is mostly being used to count internal loops.				//if(Lb1 != 0)			//Overlay.drawString("LB1 = "+Lb1, 0.05*width, 0.7*height, 0.0);		//if(Lb2 != 0)			//Overlay.drawString("LB2 = "+Lb2, 0.05*width, 0.75*height, 0.0);		//if(Lb3 != 0)			//Overlay.drawString("LB3 = "+Lb3, 0.05*width, 0.8*height, 0.0);		//if(Lb4 != 0)			//Overlay.drawString("LB4 = "+Lb4, 0.05*width, 0.85*height, 0.0);		//if(Lb5 != 0)			//Overlay.drawString("LB5 = "+Lb5, 0.05*width, 0.9*height, 0.0);			//if(g1 != 0)			//Overlay.drawString("gap1 = "+g1, 0.55*width, 0.1*height, 0.0);		//if(g2 != 0)			//Overlay.drawString("gap2 = "+g2, 0.55*width, 0.15*height, 0.0);		//if(g3 != 0)			//Overlay.drawString("gap3 = "+g3, 0.55*width, 0.2*height, 0.0);		//if(g4 != 0)			//Overlay.drawString("gap4 = "+g4, 0.55*width, 0.25*height, 0.0);			if(XAsym != 0)			saveAs("PNG", dirXAsymmetric+titlej);		else if(XSym != 0)			saveAs("PNG", dirXSymmetric+titlej);		else if(YAsym != 0)			saveAs("PNG", dirYAsymmetric+titlej);		else if(YSym != 0)			saveAs("PNG", dirYSymmetric+titlej);		else if(tLoop != 0)			saveAs("PNG", dirLoops+titlej);		else if(Circle != 0)			saveAs("PNG", dirCircle+titlej);		else if(Other != 0)			saveAs("PNG", dirOthers+titlej);		else if(IntLoop != 0)			saveAs("PNG", dirIntLoop+titlej);				else			saveAs("PNG", dirOUT+titlej);		close();	}	selectImage(imageID0);	close();	}print("--DONE!--");
