# Supplementary material for: Telomere damage induces internal loops that generate telomeric circles
Source: Nat Commun. 2020 Oct 20;11:5297. doi: 10.1038/s41467-020-19139-4 (PMC7576219; doi:10.1038/s41467-020-19139-4)
Supplement: Supplementary file 5 — Reporting Summary [file 41467_2020_19139_MOESM5_ESM.pdf]

nature research

Corresponding author(s): Ylli Doksani

Last updated by author(s): Sep 4, 2020

Reporting Summary

Nature Research wishes to improve the reproducibility of the work that we publish. This form provides structure for consistency and transparency in reporting. For further information on Nature Research policies, see our Editorial Policies and the Editorial Policy Checklist.

Statistics

For all statistical analyses, confirm that the following items are present in the figure legend, table legend, main text, or Methods section.

n/a	Confirmed	
☐	☒	The exact sample size (*n*) for each experimental group/condition, given as a discrete number and unit of measurement
☐	☒	A statement on whether measurements were taken from distinct samples or whether the same sample was measured repeatedly
☐	☒	The statistical test(s) used AND whether they are one- or two-sided<br>*Only common tests should be described solely by name; describe more complex techniques in the Methods section.*
☒	☐	A description of all covariates tested
☒	☐	A description of any assumptions or corrections, such as tests of normality and adjustment for multiple comparisons
☐	☒	A full description of the statistical parameters including central tendency (e.g. means) or other basic estimates (e.g. regression coefficient) AND variation (e.g. standard deviation) or associated estimates of uncertainty (e.g. confidence intervals)
☐	☒	For null hypothesis testing, the test statistic (e.g. *F*, *t*, *r*) with confidence intervals, effect sizes, degrees of freedom and *P* value noted<br>*Give P values as exact values whenever suitable.*
☒	☐	For Bayesian analysis, information on the choice of priors and Markov chain Monte Carlo settings
☒	☐	For hierarchical and complex designs, identification of the appropriate level for tests and full reporting of outcomes
☒	☐	Estimates of effect sizes (e.g. Cohen's *d*, Pearson's *r*), indicating how they were calculated

Our web collection on statistics for biologists contains articles on many of the points above.

Software and code

Policy information about availability of computer code

Data collection	-TEM pictures were taken using a FEI Tecnai12 Bio twin microscope operated at 120 KV and equipped with a side-mounted GATAN Orius SC-1000 camera controlled by the Digital Micrograph software v2.3.3. 64-Bit.<br>-Ethidium Bromide gel acquisitions were performed with a Chemidoc XRS+ Imaging system and Image Lab software (v3.0, Bio-Rad Laboratories).<br>-Telomere fiber analysis images were acquired in DV Elite system (GE Healthcare) equipped with a IX71 microscope (Olympus) and a sCMOS camera and driven by softWoRx version 7.0.0 or in a CSU spinning disk confocal microscope (Olympus) Camera Ixon Ultra 897 (Andor) Laser lines 405nm, 488nm, 561nm, 640nm Software cellSens Dimension 1.18<br>-Radioactive signal was captured on phosphor screens (FUJIFILM Storage Phosphor screen MS3543 E), read on a Typhon Trio (GE) scanner software version 2.0.0.6.
Data analysis	- All digital images were analyzed in FIJI/ImageJ software v2.0.0-rc-69/1.52p<br>- Statistical analysis were performed with Prism (v6.0c, Graph Pad).

For manuscripts utilizing custom algorithms or software that are central to the research but not yet described in published literature, software must be made available to editors and reviewers. We strongly encourage code deposition in a community repository (e.g. GitHub). See the Nature Research guidelines for submitting code & software for further information.

Data

Policy information about availability of data

All manuscripts must include a data availability statement. This statement should provide the following information, where applicable:

- Accession codes, unique identifiers, or web links for publicly available datasets
- A list of figures that have associated raw data
- A description of any restrictions on data availability

All data generated or analyzed during this study are included in this published article (and its supplementary information files or source data files). All raw EM images analyzed are available from the corresponding author upon reasonable request.

Field-specific reporting

Please select the one below that is the best fit for your research. If you are not sure, read the appropriate sections before making your selection.

☒ Life sciences ☐ Behavioural & social sciences ☐ Ecological, evolutionary & environmental sciences

For a reference copy of the document with all sections, see nature.com/documents/nr-reporting-summary-flat.pdf

Life sciences study design

All studies must disclose on these points even when the disclosure is negative.

Sample size	Sample size was determined according to previously conducted experiments (Zellweger, R. & Lopes, M. Dynamic Architecture of Eukaryotic DNA Replication Forks In Vivo, Visualized by Electron Microscopy. Methods Mol Biol 1672, 261-294, doi:10.1007/978-1-4939-7306-4_19 (2018)) and determined to be adequate based on the magnitude and consistency of measurable differences between groups. The number of experiments (n) is indicated in each figure legend.
Data exclusions	No data were excluded from the analysis.
Replication	Each of the finding has been reproduced at least 3 times unless otherwise specified. All attempts at replication were successful.
Randomization	This work did not involve group analyses that require randomization.
Blinding	Initial observation of i-loops in telomere enriched samples was not hypothesis-based and therefore not subjective. The data were confirmed in blind acquisition and analysis. Acquisition and analysis of other data in this work was not subjective.

Reporting for specific materials, systems and methods

We require information from authors about some types of materials, experimental systems and methods used in many studies. Here, indicate whether each material, system or method listed is relevant to your study. If you are not sure if a list item applies to your research, read the appropriate section before selecting a response.

Materials & experimental systems

n/a	Involved in the study	
☐	☒	Antibodies
☐	☒	Eukaryotic cell lines
☒	☐	Palaeontology and archaeology
☒	☐	Animals and other organisms
☒	☐	Human research participants
☒	☐	Clinical data
☒	☐	Dual use research of concern

Methods

n/a	Involved in the study	
☒	☐	ChIP-seq
☒	☐	Flow cytometry
☒	☐	MRI-based neuroimaging

Antibodies

Antibodies used	Anti-DNA Antibody, single stranded, clone 16-19, Millipore MAB3034, LOT N: 2899758
Validation	Details are provided in the Methods section of the paper.

Eukaryotic cell lines

Policy information about cell lines

Cell line source(s)	U2OS cells were obtained by ATCC. SV40-LT immortalized MEFs were generated by Y.D. in the Titia de Lange laboratory. HeLa1.3, HeLa 204 and HTC75 are a gift from Titia de Lange
Authentication	U2OS cells were authenticated using the GenePrint® 10 System (10-Locus STR System for Cell Line Authentication) by Promega CAT. NUM. B9510. The relevant propertied of HeLa1.3, HeLa204 and HTC75 in this study (i.e. telomere length) was verified in telomere blots.
Mycoplasma contamination	All cell lines are tested for mycoplasma both upon arrival at IFOM and after a new stock of cells is made, and all of them resulted to be negative for mycoplasma contamination. Mycoplasma test is performed by the IFOM Cell Biology UNIT and consist of two independent tests: a PCR analysis (For detail protocol see PMID: 21516400) and a biochemical test (MycoAlert Detection Kit, Lonza Catalog #: LT07-418).
Commonly misidentified lines (See ICLAC register)	No commonly-misidentified cell lines were used in this study
