# Supplementary material for: Telomere damage induces internal loops that generate telomeric circles
Source: Nat Commun. 2020 Oct 20;11:5297. doi: 10.1038/s41467-020-19139-4 (PMC7576219; doi:10.1038/s41467-020-19139-4)
Supplement: Supplementary file 6 — Source Data [file 41467_2020_19139_MOESM6_ESM.zip › Source data 2nd rev/Source data Figure 2.docx]

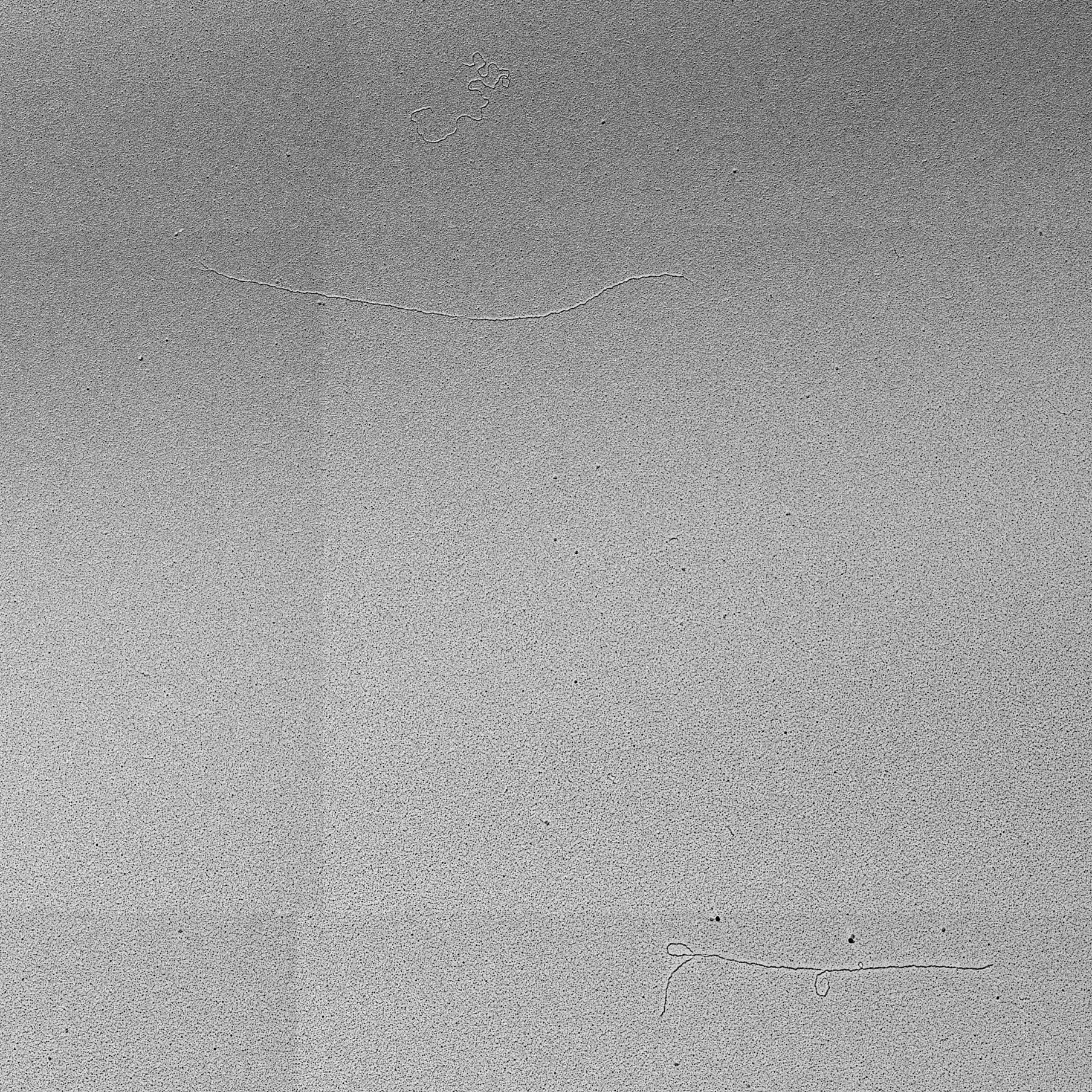

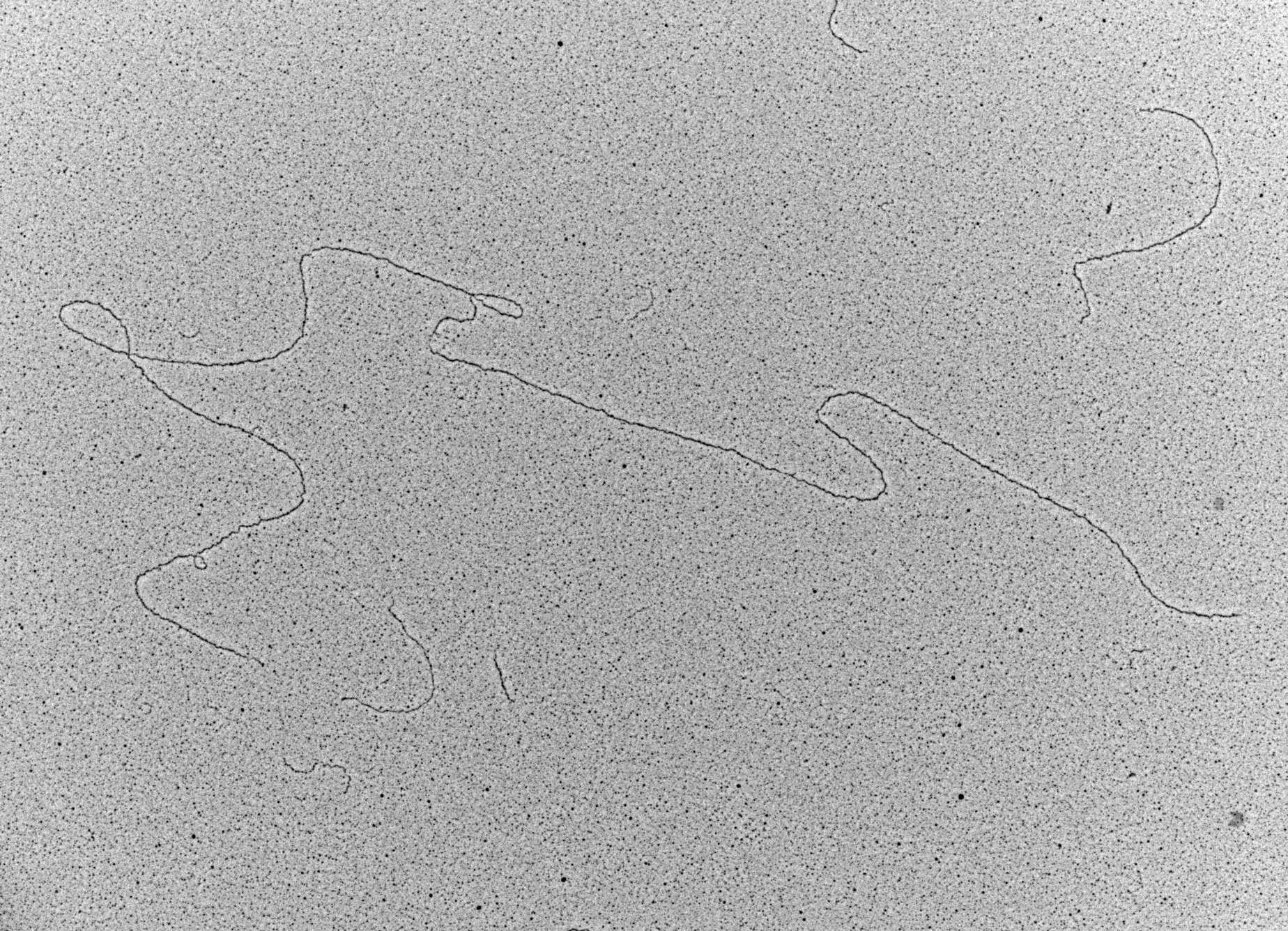

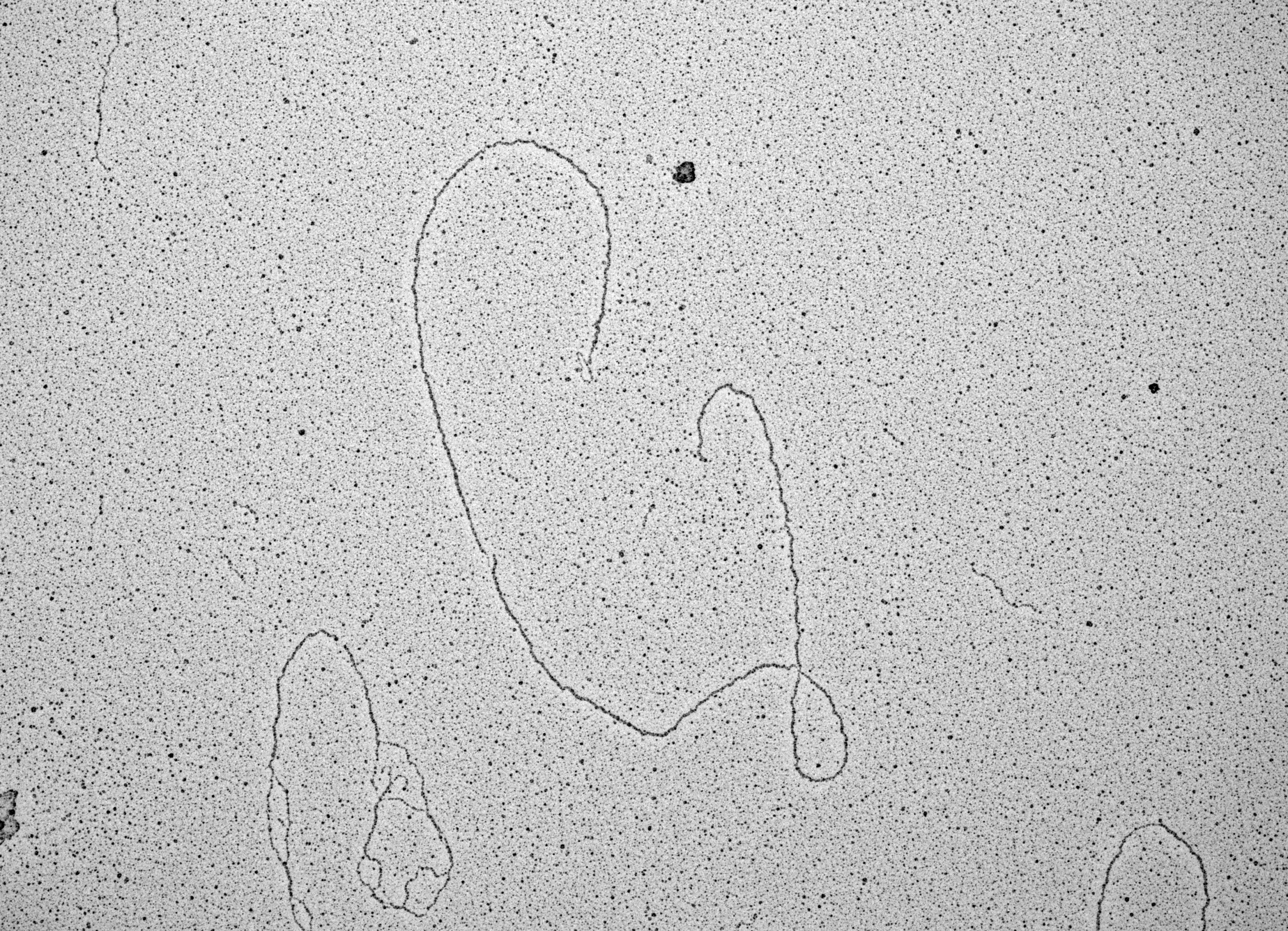

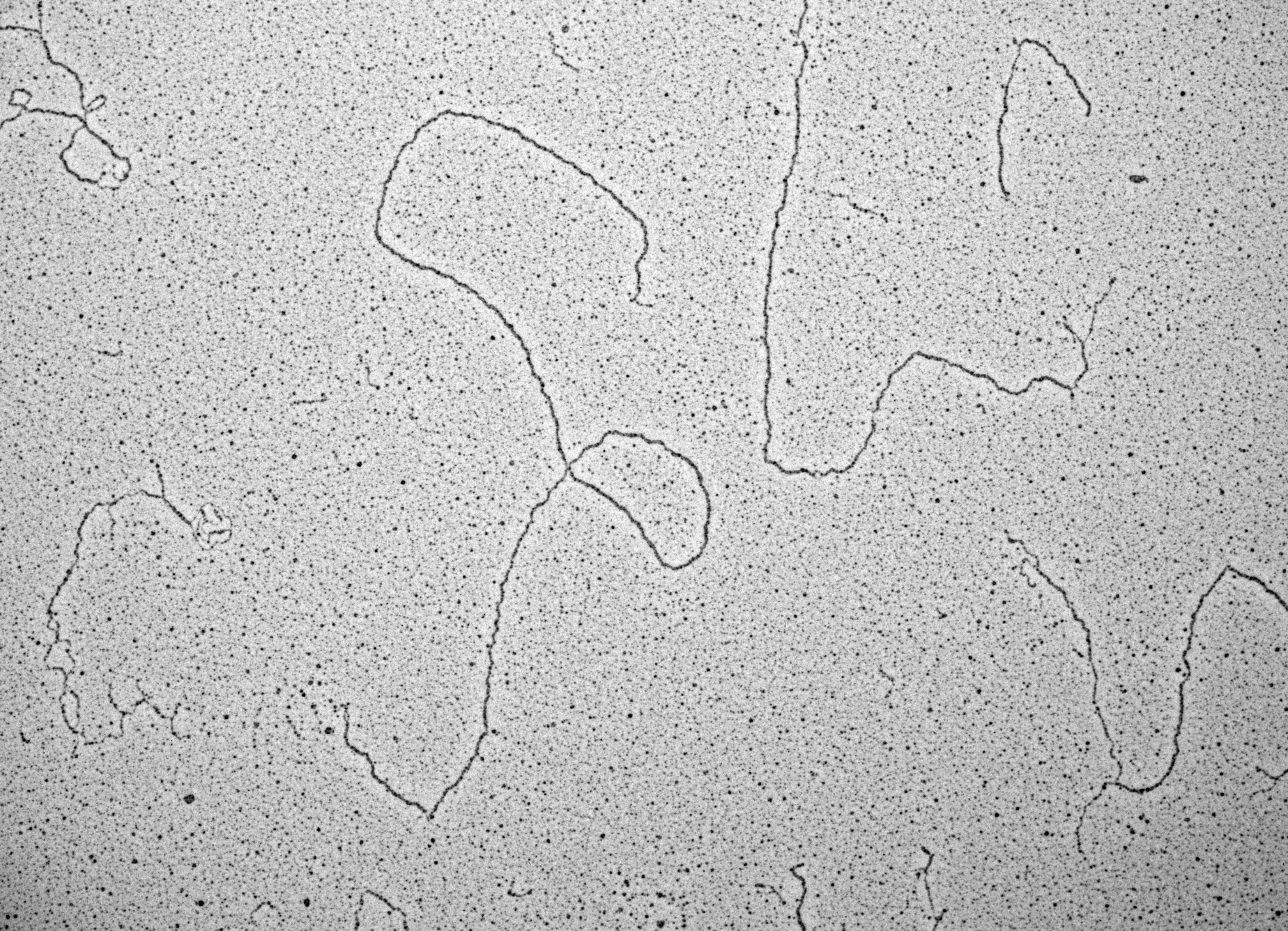

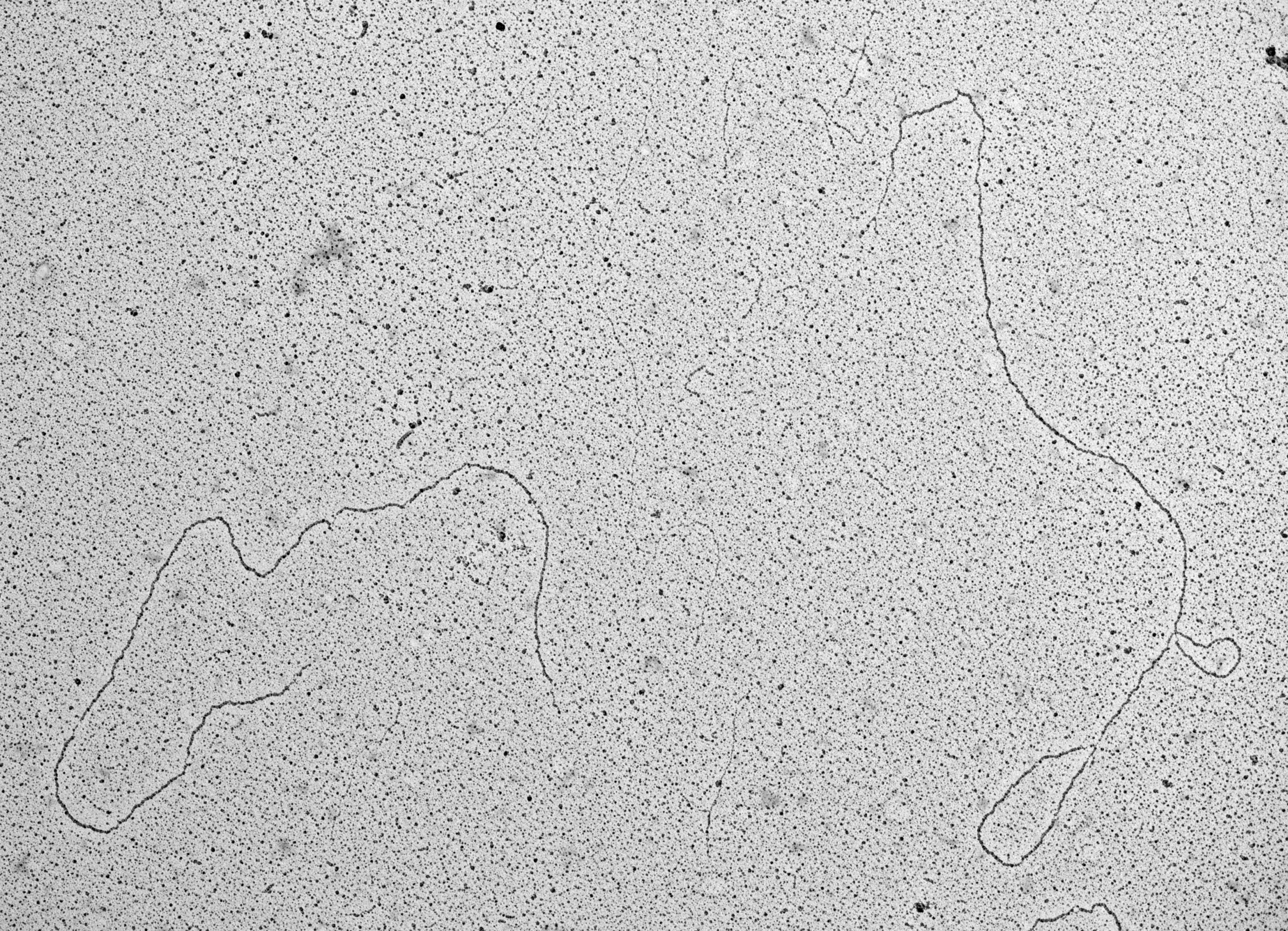

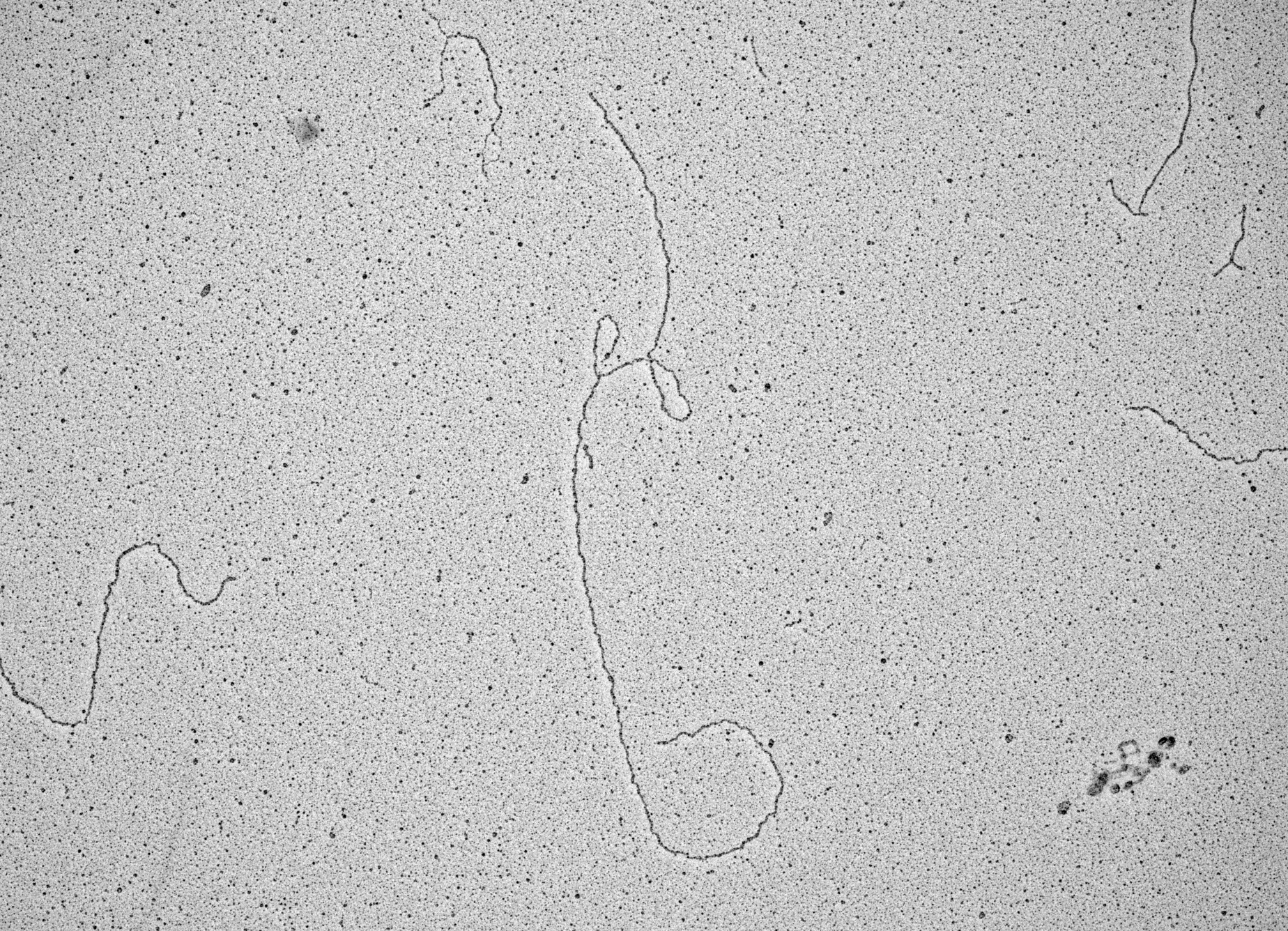
Figure 2a

| **Figure 2B** |  |  |  |  |
| --- | --- | --- | --- | --- |
|  |  |  |  |  |
|  |  | N (molecules counted) | | % of molecules with internal loops |
| telomere-enriched | Exp1 | 310 |  | 16.13 |
| telomere-enriched | Exp2 | 715 |  | 15.38 |
| telomere-enriched | Exp3 | 845 |  | 12.42 |
|  |  |  |  |  |
| Bulk | Exp1 | 1466 |  | 3.55 |
| Bulk | Exp2 | 776 |  | 3.74 |
| Bulk | Exp3 | 1776 |  | 1.69 |

| **Figure 2C** | i-loop size distribution (kb) |
| --- | --- |
|  |  |
|  | 26.55556 |
|  | 19.58333 |
|  | 16.36111 |
|  | 15.38889 |
|  | 14.33333 |
|  | 13.61111 |
|  | 10.33333 |
|  | 10.27778 |
|  | 8.5 |
|  | 8.388889 |
|  | 8.055556 |
|  | 7.666667 |
|  | 6.722222 |
|  | 6.388889 |
|  | 6.361111 |
|  | 6.166667 |
|  | 5.611111 |
|  | 5.388889 |
|  | 5.305556 |
|  | 5.222222 |
|  | 5.166667 |
|  | 5.111111 |
|  | 5.055556 |
|  | 4.972222 |
|  | 4.444444 |
|  | 4.111111 |
|  | 3.916667 |
|  | 3.638889 |
|  | 3.194444 |
|  | 3.083333 |
|  | 3.055556 |
|  | 2.805556 |
|  | 2.666667 |
|  | 2.611111 |
|  | 1.833333 |
|  | 1.75 |
|  | 1.277778 |
|  | 0.833333 |
|  | 0.777778 |
|  | 0.722222 |
|  | 0.666667 |
|  | 0.638889 |
|  | 0.638889 |
|  | 0.611111 |
|  | 0.555556 |
|  | 0.5 |
|  | 0.472222 |
|  | 0.388889 |
|  | 0.333333 |
|  | 0.333333 |
|  | 9.444444 |
|  | 5.388889 |
|  | 4 |
|  | 3.166667 |
|  | 2.416667 |
|  | 2.111111 |
|  | 1.944444 |
|  | 1.916667 |
|  | 1.833333 |
|  | 1.583333 |
|  | 1.472222 |
|  | 1.222222 |
|  | 0.416667 |
|  | 14.27778 |
|  | 2.416667 |
|  | 0.666667 |
|  | 0.277778 |
|  | 0.222222 |
|  | 20.38889 |
|  | 18.22222 |
|  | 15.91667 |
|  | 14.83333 |
|  | 14.16667 |
|  | 12.11111 |
|  | 11.77778 |
|  | 10.44444 |
|  | 9 |
|  | 8.861111 |
|  | 8.694444 |
|  | 6.361111 |
|  | 6.055556 |
|  | 5.694444 |
|  | 4.694444 |
|  | 4.5 |
|  | 4.444444 |
|  | 4.333333 |
|  | 4.333333 |
|  | 3.944444 |
|  | 3.916667 |
|  | 3.833333 |
|  | 3.694444 |
|  | 3.555556 |
|  | 3.527778 |
|  | 3.5 |
|  | 3.222222 |
|  | 3.055556 |
|  | 3.055556 |
|  | 2.944444 |
|  | 2.888889 |
|  | 2.694444 |
|  | 2.666667 |
|  | 2.527778 |
|  | 2.5 |
|  | 2.388889 |
|  | 2.277778 |
|  | 2.25 |
|  | 2.138889 |
|  | 2.138889 |
|  | 2.083333 |
|  | 2.083333 |
|  | 2.055556 |
|  | 2.055556 |
|  | 2.055556 |
|  | 2.027778 |
|  | 2 |
|  | 1.972222 |
|  | 1.916667 |
|  | 1.916667 |
|  | 1.861111 |
|  | 1.861111 |
|  | 1.805556 |
|  | 1.75 |
|  | 1.75 |
|  | 1.694444 |
|  | 1.694444 |
|  | 1.666667 |
|  | 1.666667 |
|  | 1.638889 |
|  | 1.611111 |
|  | 1.555556 |
|  | 1.527778 |
|  | 1.5 |
|  | 1.444444 |
|  | 1.416667 |
|  | 1.388889 |
|  | 1.333333 |
|  | 1.333333 |
|  | 1.305556 |
|  | 1.277778 |
|  | 1.277778 |
|  | 1.166667 |
|  | 1.138889 |
|  | 1.083333 |
|  | 1.083333 |
|  | 1.055556 |
|  | 1.055556 |
|  | 1.055556 |
|  | 1.027778 |
|  | 0.972222 |
|  | 0.888889 |
|  | 0.888889 |
|  | 0.861111 |
|  | 0.777778 |
|  | 0.75 |
|  | 0.694444 |
|  | 0.666667 |
|  | 0.666667 |
|  | 0.666667 |
|  | 0.638889 |
|  | 0.555556 |
|  | 0.555556 |
|  | 0.555556 |
|  | 0.416667 |
|  | 0.388889 |
|  | 0.333333 |
|  | 0.333333 |
|  | 6.138889 |
|  | 5.694444 |
|  | 5 |
|  | 4.888889 |
|  | 4.805556 |
|  | 3.333333 |
|  | 3.277778 |
|  | 3.25 |
|  | 2.777778 |
|  | 2.555556 |
|  | 2.111111 |
|  | 2.083333 |
|  | 2.027778 |
|  | 1.888889 |
|  | 1.75 |
|  | 1.444444 |
|  | 1.388889 |
|  | 1.222222 |
|  | 1.222222 |
|  | 1.166667 |
|  | 1.027778 |
|  | 0.888889 |
|  | 0.805556 |
|  | 0.805556 |
|  | 0.666667 |
|  | 0.638889 |
|  | 0.583333 |
|  | 0.444444 |
|  | 0.444444 |
|  | 0.416667 |
|  | 0.166667 |
|  | 6.194444 |
|  | 2.861111 |
|  | 1.722222 |
|  | 1.277778 |
|  | 0.75 |
|  | 0.555556 |
|  | 17.5 |
|  | 12.36111 |
|  | 11.47222 |
|  | 10.66667 |
|  | 10.41667 |
|  | 7.194444 |
|  | 6.416667 |
|  | 5.805556 |
|  | 5.638889 |
|  | 5.027778 |
|  | 4.777778 |
|  | 4.611111 |
|  | 4.5 |
|  | 4.444444 |
|  | 4.361111 |
|  | 4.277778 |
|  | 4.222222 |
|  | 3.583333 |
|  | 3.138889 |
|  | 2.833333 |
|  | 2.805556 |
|  | 2.388889 |
|  | 2.25 |
|  | 2.222222 |
|  | 2.138889 |
|  | 2.027778 |
|  | 2 |
|  | 1.972222 |
|  | 1.888889 |
|  | 1.75 |
|  | 1.722222 |
|  | 1.638889 |
|  | 1.611111 |
|  | 1.583333 |
|  | 1.555556 |
|  | 1.527778 |
|  | 1.527778 |
|  | 1.527778 |
|  | 1.527778 |
|  | 1.5 |
|  | 1.444444 |
|  | 1.361111 |
|  | 1.333333 |
|  | 1.305556 |
|  | 1.305556 |
|  | 1.277778 |
|  | 1.277778 |
|  | 1.25 |
|  | 1.222222 |
|  | 1.166667 |
|  | 1.111111 |
|  | 1.083333 |
|  | 1.083333 |
|  | 1.083333 |
|  | 1.055556 |
|  | 1.055556 |
|  | 1.055556 |
|  | 1.027778 |
|  | 0.972222 |
|  | 0.972222 |
|  | 0.916667 |
|  | 0.916667 |
|  | 0.888889 |
|  | 0.861111 |
|  | 0.861111 |
|  | 0.861111 |
|  | 0.805556 |
|  | 0.805556 |
|  | 0.777778 |
|  | 0.75 |
|  | 0.722222 |
|  | 0.722222 |
|  | 0.722222 |
|  | 0.694444 |
|  | 0.694444 |
|  | 0.666667 |
|  | 0.583333 |
|  | 0.555556 |
|  | 0.555556 |
|  | 0.555556 |
|  | 0.527778 |
|  | 0.527778 |
|  | 0.527778 |
|  | 0.527778 |
|  | 0.527778 |
|  | 0.527778 |
|  | 0.527778 |
|  | 0.5 |
|  | 0.5 |
|  | 0.5 |
|  | 0.472222 |
|  | 0.472222 |
|  | 0.388889 |
|  | 0.388889 |
|  | 0.361111 |
|  | 0.333333 |
|  | 0.333333 |
|  | 0.305556 |
|  | 0.277778 |
|  | 0.277778 |
|  | 0.25 |
|  | 0.25 |
|  | 0.25 |
|  | 0.25 |
|  | 0.222222 |
|  | 0.194444 |
|  | 7.5 |
|  | 7.277778 |
|  | 6.861111 |
|  | 5.25 |
|  | 2.638889 |
|  | 2.083333 |
|  | 1.861111 |
|  | 1.75 |
|  | 1.388889 |
|  | 1.305556 |
|  | 1.305556 |
|  | 1.222222 |
|  | 1.194444 |
|  | 1.138889 |
|  | 1.083333 |
|  | 1.027778 |
|  | 0.75 |
|  | 0.638889 |
|  | 0.611111 |
|  | 0.611111 |
|  | 0.5 |
|  | 0.277778 |
|  | 0.222222 |
|  | 11.33333 |
|  | 4 |
|  | 3.833333 |
|  | 3.027778 |
|  | 1.583333 |
|  | 1.5 |
|  | 1.305556 |
|  | 1.166667 |
|  | 5 |
|  | 2.444444 |

| **Figure 2D** | number of i-loops per molecule | |
| --- | --- | --- |
|  |  |  |
|  | 4 |  |
|  | 4 |  |
|  | 3 |  |
|  | 3 |  |
|  | 3 |  |
|  | 3 |  |
|  | 3 |  |
|  | 3 |  |
|  | 3 |  |
|  | 3 |  |
|  | 3 |  |
|  | 3 |  |
|  | 3 |  |
|  | 3 |  |
|  | 3 |  |
|  | 3 |  |
|  | 3 |  |
|  | 2 |  |
|  | 2 |  |
|  | 2 |  |
|  | 2 |  |
|  | 2 |  |
|  | 2 |  |
|  | 2 |  |
|  | 2 |  |
|  | 2 |  |
|  | 2 |  |
|  | 2 |  |
|  | 2 |  |
|  | 2 |  |
|  | 2 |  |
|  | 2 |  |
|  | 2 |  |
|  | 2 |  |
|  | 2 |  |
|  | 2 |  |
|  | 2 |  |
|  | 2 |  |
|  | 2 |  |
|  | 2 |  |
|  | 2 |  |
|  | 2 |  |
|  | 2 |  |
|  | 2 |  |
|  | 2 |  |
|  | 2 |  |
|  | 2 |  |
|  | 2 |  |
|  | 2 |  |
|  | 2 |  |
|  | 2 |  |
|  | 2 |  |
|  | 2 |  |
|  | 2 |  |
|  | 2 |  |
|  | 2 |  |
|  | 2 |  |
|  | 2 |  |
|  | 2 |  |
|  | 2 |  |
|  | 2 |  |
|  | 2 |  |
|  | 2 |  |
|  | 2 |  |
|  | 2 |  |
|  | 2 |  |
|  | 2 |  |
|  | 2 |  |
|  | 1 |  |
|  | 1 |  |
|  | 1 |  |
|  | 1 |  |
|  | 1 |  |
|  | 1 |  |
|  | 1 |  |
|  | 1 |  |
|  | 1 |  |
|  | 1 |  |
|  | 1 |  |
|  | 1 |  |
|  | 1 |  |
|  | 1 |  |
|  | 1 |  |
|  | 1 |  |
|  | 1 |  |
|  | 1 |  |
|  | 1 |  |
|  | 1 |  |
|  | 1 |  |
|  | 1 |  |
|  | 1 |  |
|  | 1 |  |
|  | 1 |  |
|  | 1 |  |
|  | 1 |  |
|  | 1 |  |
|  | 1 |  |
|  | 1 |  |
|  | 1 |  |
|  | 1 |  |
|  | 1 |  |
|  | 1 |  |
|  | 1 |  |
|  | 1 |  |
|  | 1 |  |
|  | 1 |  |
|  | 1 |  |
|  | 1 |  |
|  | 1 |  |
|  | 1 |  |
|  | 1 |  |
|  | 1 |  |
|  | 1 |  |
|  | 1 |  |
|  | 1 |  |
|  | 1 |  |
|  | 1 |  |
|  | 1 |  |
|  | 1 |  |
|  | 1 |  |
|  | 1 |  |
|  | 1 |  |
|  | 1 |  |
|  | 1 |  |
|  | 1 |  |
|  | 1 |  |
|  | 1 |  |
|  | 1 |  |
|  | 1 |  |
|  | 1 |  |
|  | 1 |  |
|  | 1 |  |
|  | 1 |  |
|  | 1 |  |
|  | 1 |  |
|  | 1 |  |
|  | 1 |  |
|  | 1 |  |
|  | 1 |  |
|  | 1 |  |
|  | 1 |  |
|  | 1 |  |
|  | 1 |  |
|  | 1 |  |
|  | 1 |  |
|  | 1 |  |
|  | 1 |  |
|  | 1 |  |
|  | 1 |  |
|  | 1 |  |
|  | 1 |  |
|  | 1 |  |
|  | 1 |  |
|  | 1 |  |
|  | 1 |  |
|  | 1 |  |
|  | 1 |  |
|  | 1 |  |
|  | 1 |  |
|  | 1 |  |
|  | 1 |  |
|  | 1 |  |
|  | 1 |  |
|  | 1 |  |
|  | 1 |  |
|  | 1 |  |
|  | 1 |  |
|  | 1 |  |
|  | 1 |  |
|  | 1 |  |
|  | 1 |  |
|  | 1 |  |
|  | 1 |  |
|  | 1 |  |
|  | 1 |  |
|  | 1 |  |
|  | 1 |  |
|  | 1 |  |
|  | 1 |  |
|  | 1 |  |
|  | 1 |  |
|  | 1 |  |
|  | 1 |  |
|  | 1 |  |
|  | 1 |  |
|  | 1 |  |
|  | 1 |  |
|  | 1 |  |
|  | 1 |  |
|  | 1 |  |
|  | 1 |  |
|  | 1 |  |
|  | 1 |  |
|  | 1 |  |
|  | 1 |  |
|  | 1 |  |
|  | 1 |  |
|  | 1 |  |
|  | 1 |  |
|  | 1 |  |
|  | 1 |  |
|  | 1 |  |
|  | 1 |  |
|  | 1 |  |
|  | 1 |  |
|  | 1 |  |
|  | 1 |  |
|  | 1 |  |
|  | 1 |  |
|  | 1 |  |
|  | 1 |  |
|  | 1 |  |
|  | 1 |  |
|  | 1 |  |
|  | 1 |  |
|  | 1 |  |
|  | 1 |  |
|  | 1 |  |
|  | 1 |  |
|  | 1 |  |
|  | 1 |  |
|  | 1 |  |
|  | 1 |  |
|  | 1 |  |
|  | 1 |  |
|  | 1 |  |
|  | 1 |  |
|  | 1 |  |
|  | 1 |  |
|  | 1 |  |
|  | 1 |  |
|  | 1 |  |
|  | 1 |  |
|  | 1 |  |
|  | 1 |  |
|  | 1 |  |
|  | 1 |  |
|  | 1 |  |
|  | 1 |  |
|  | 1 |  |
|  | 1 |  |
|  | 1 |  |
|  | 1 |  |
|  | 1 |  |
|  | 1 |  |
|  | 1 |  |
|  | 1 |  |
|  | 1 |  |
|  | 1 |  |
|  | 1 |  |
|  | 1 |  |
|  | 1 |  |
|  | 1 |  |
|  | 1 |  |
|  | 1 |  |
|  | 1 |  |
|  | 1 |  |
|  | 1 |  |
|  | 1 |  |
|  | 1 |  |
|  | 1 |  |
|  | 1 |  |
|  | 1 |  |
|  | 1 |  |
|  | 1 |  |
|  | 1 |  |
