# Supplementary material for: Telomere damage induces internal loops that generate telomeric circles
Source: Nat Commun. 2020 Oct 20;11:5297. doi: 10.1038/s41467-020-19139-4 (PMC7576219; doi:10.1038/s41467-020-19139-4)

Figure 5A

DNaseI treatment in MEFs nuclei, telomeric probe

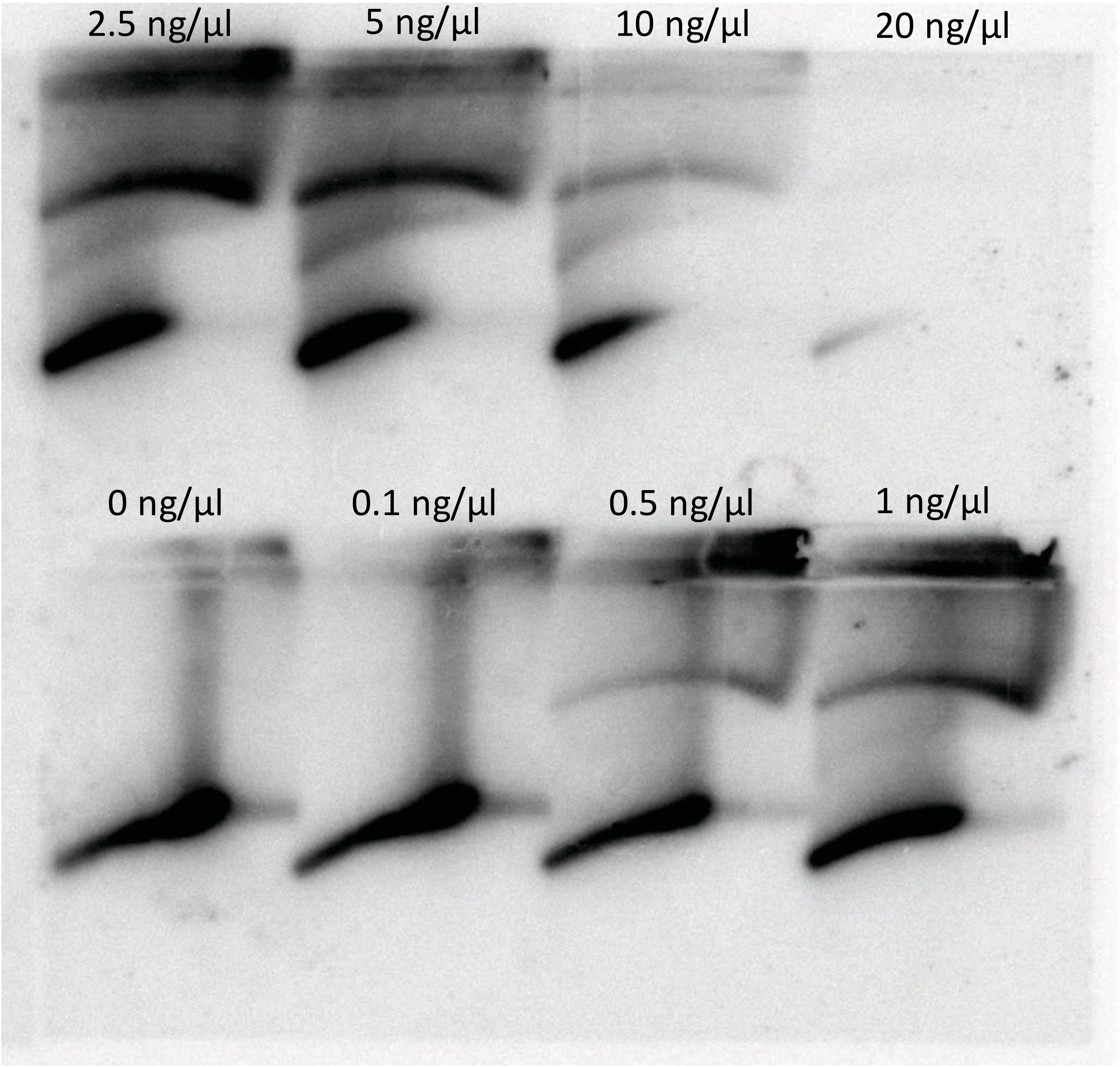

Figure 5B

DNaseI treatment on isolated mouse DNA

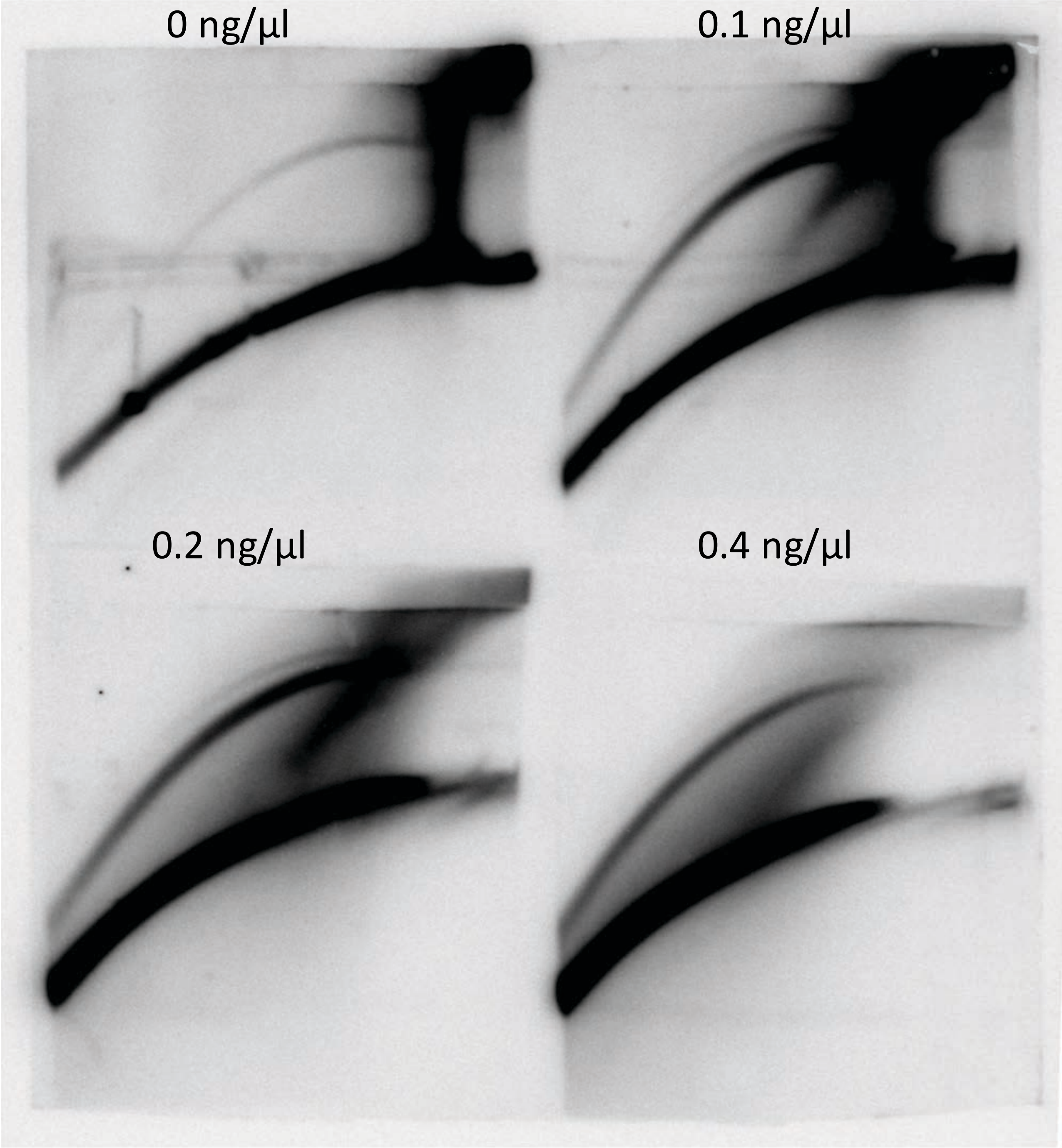

Figure 5C

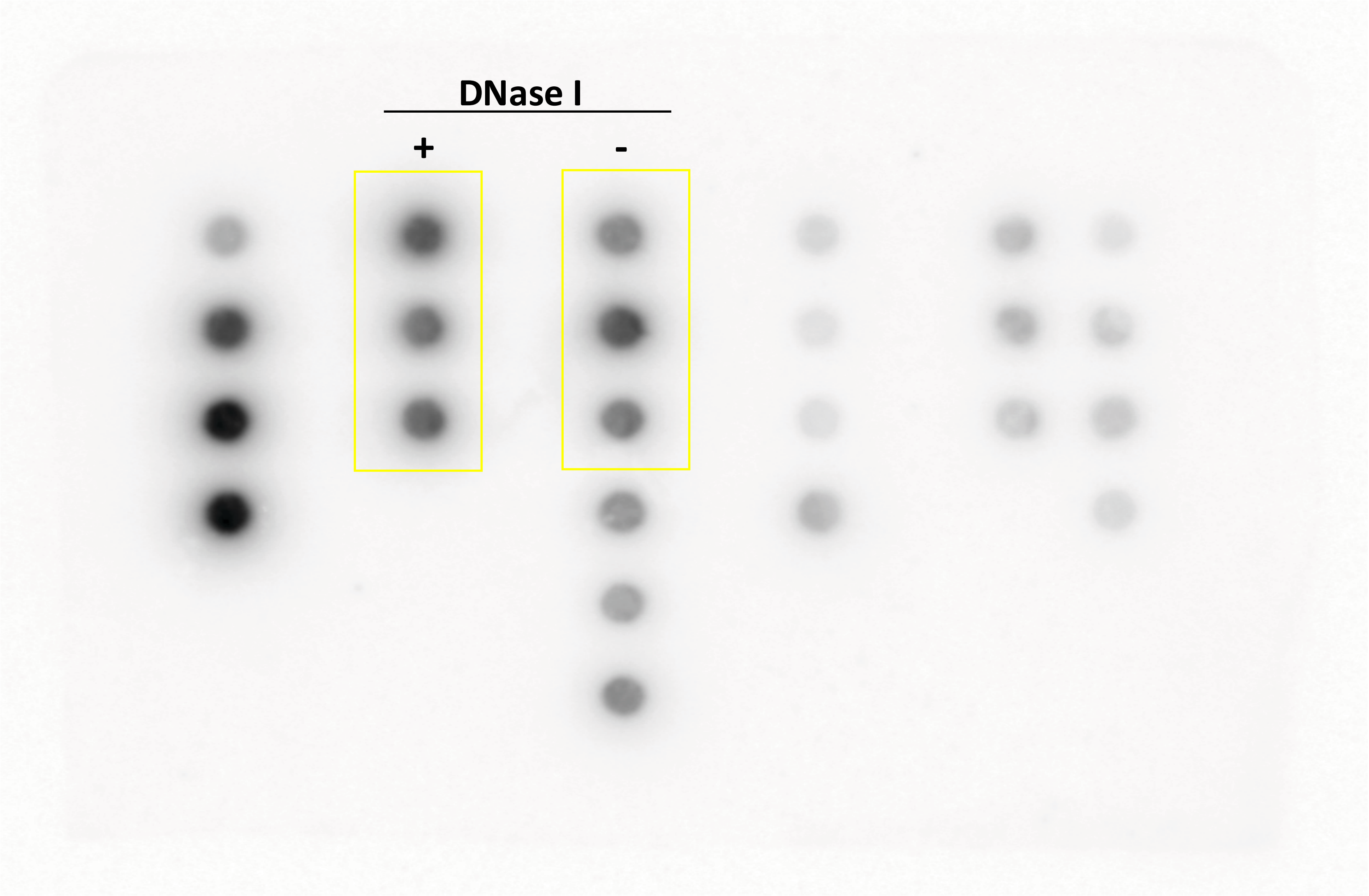

Figure 5e

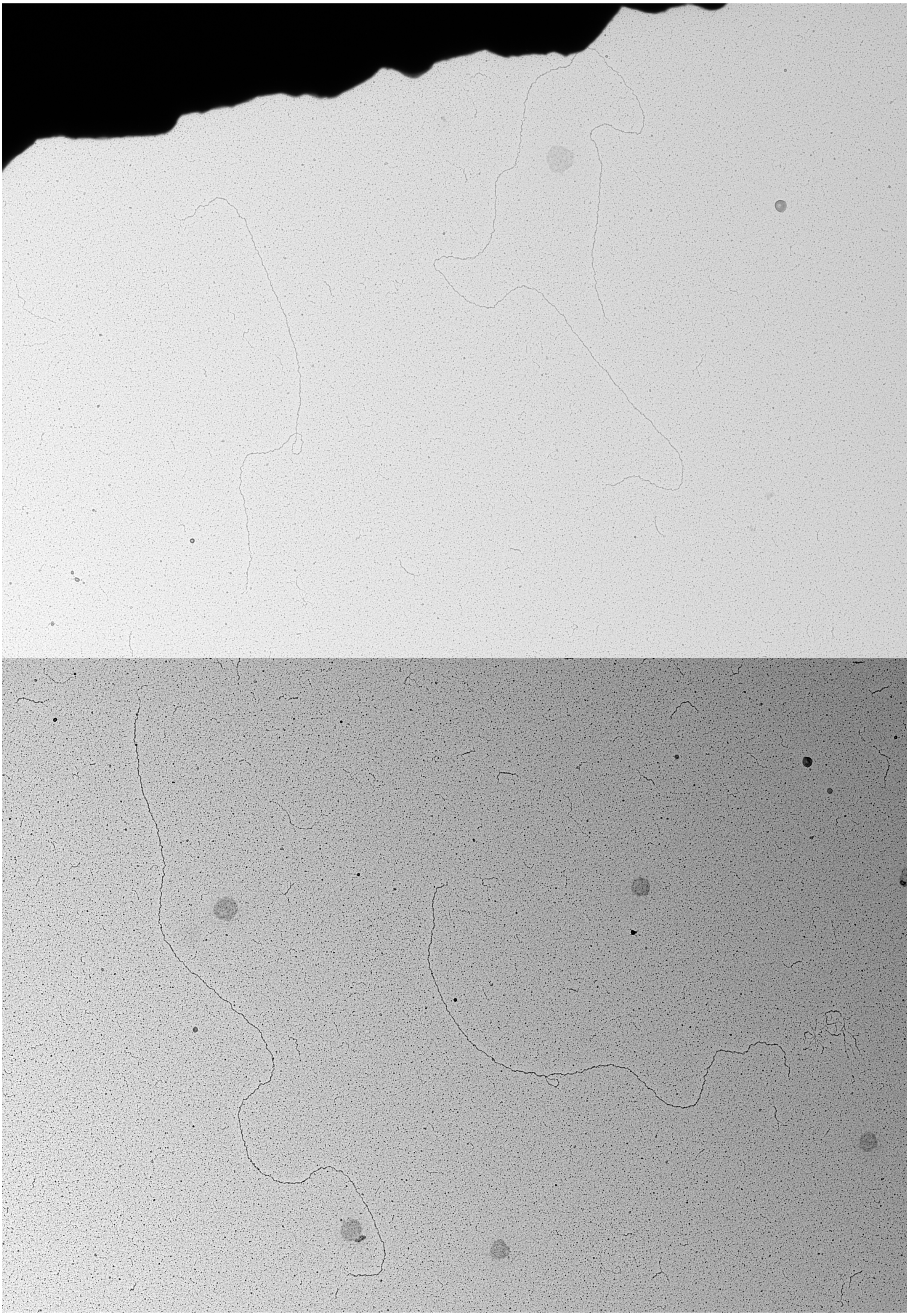

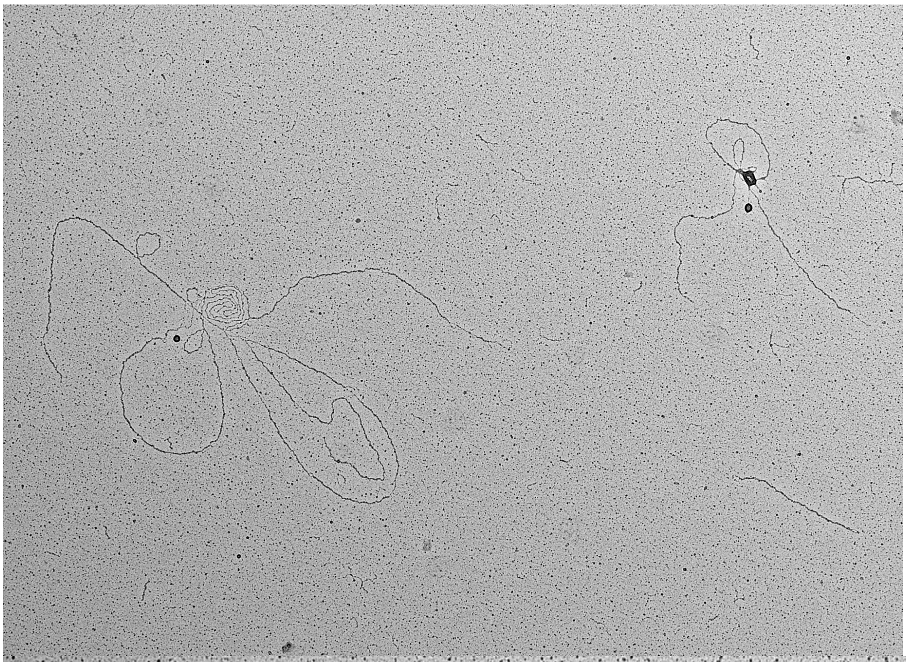
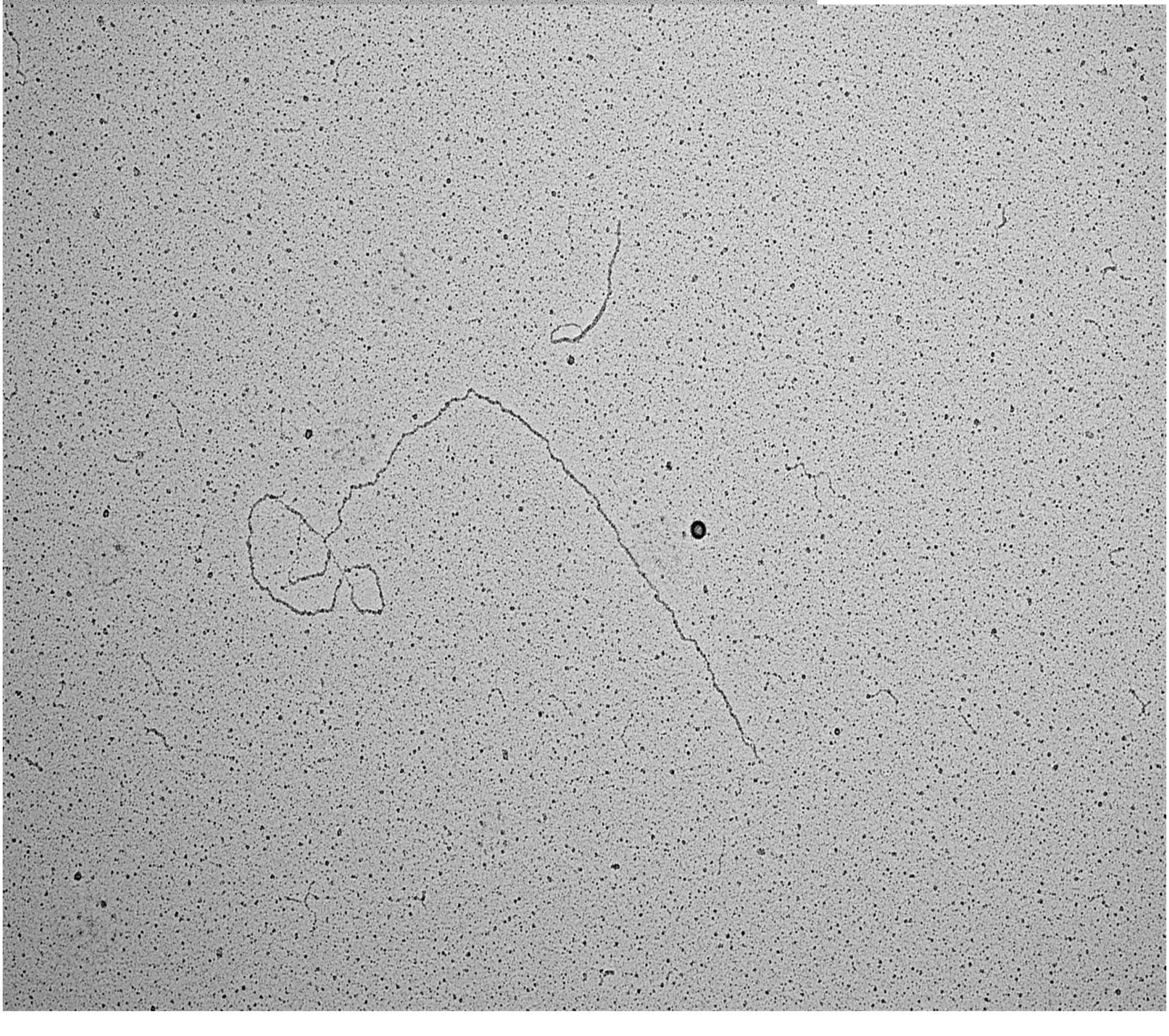

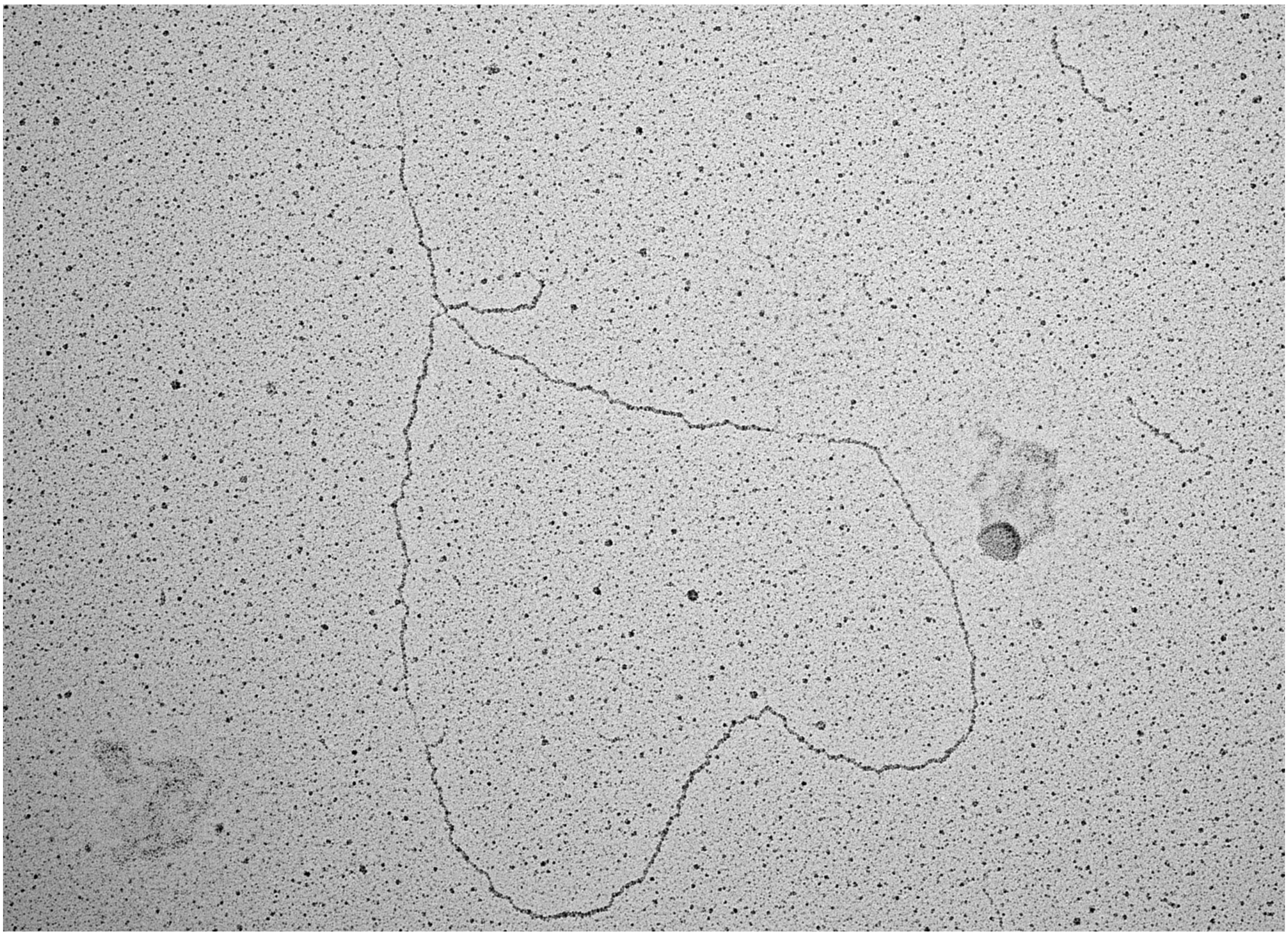

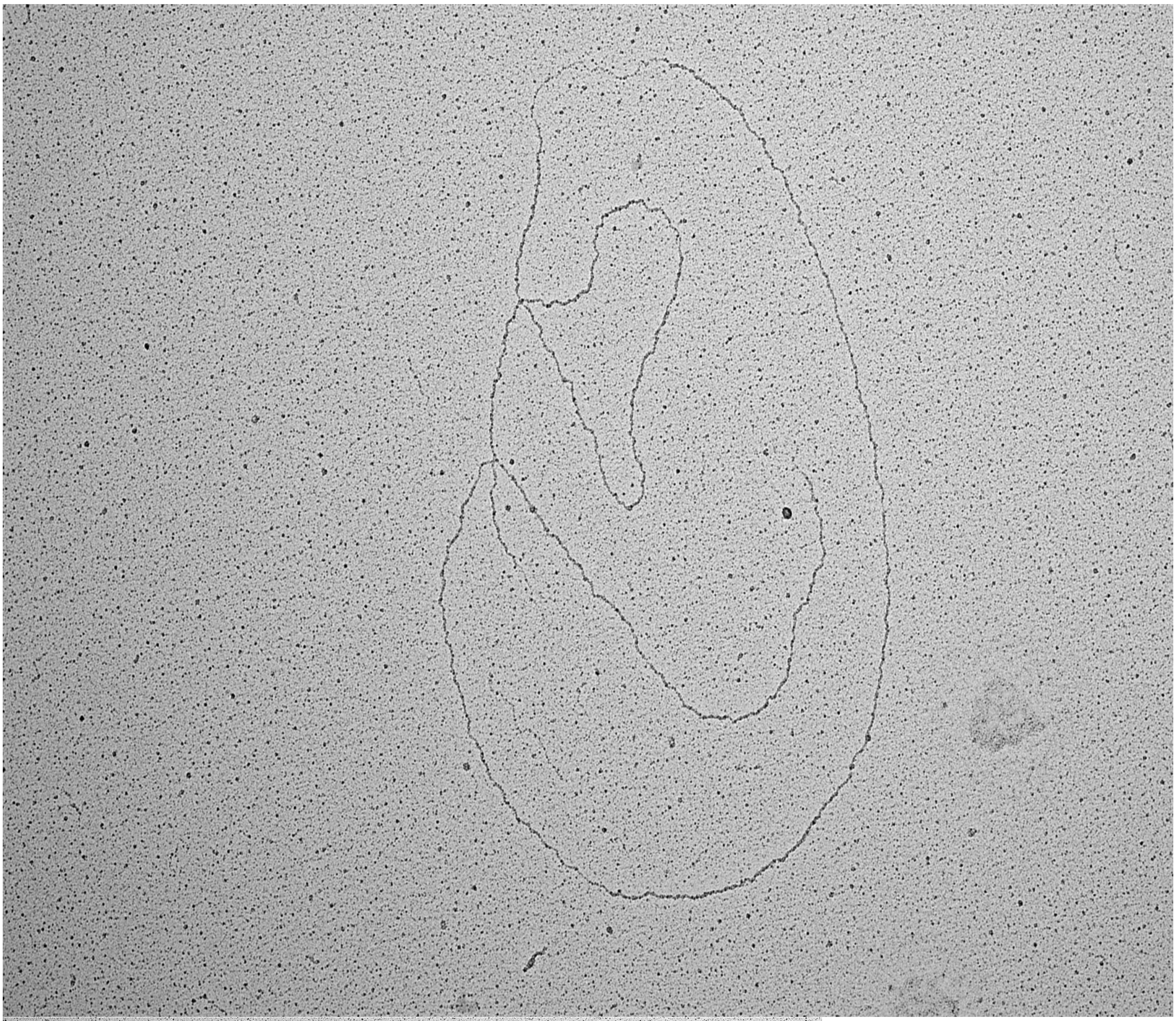

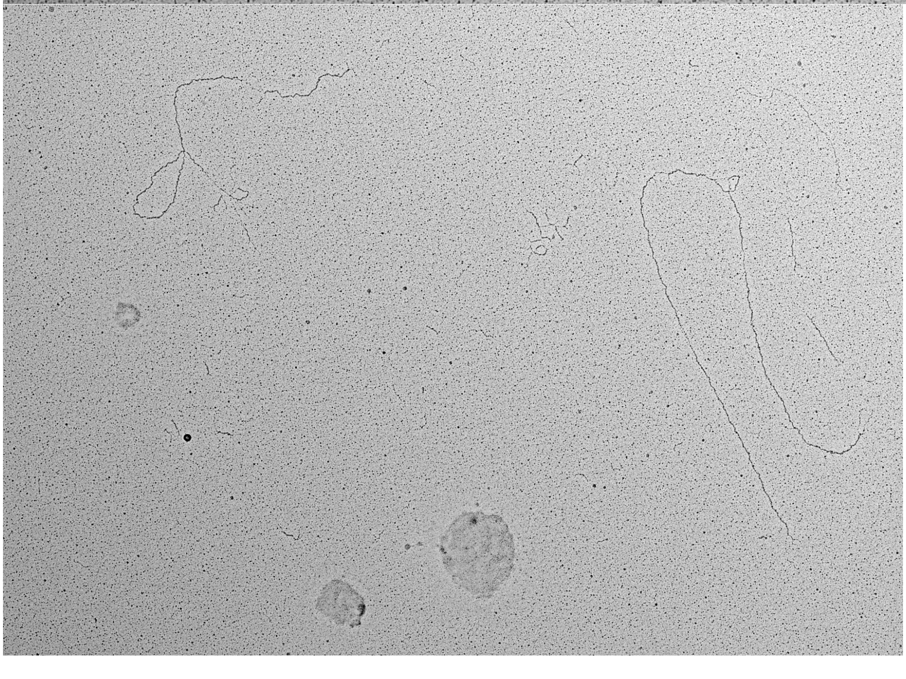

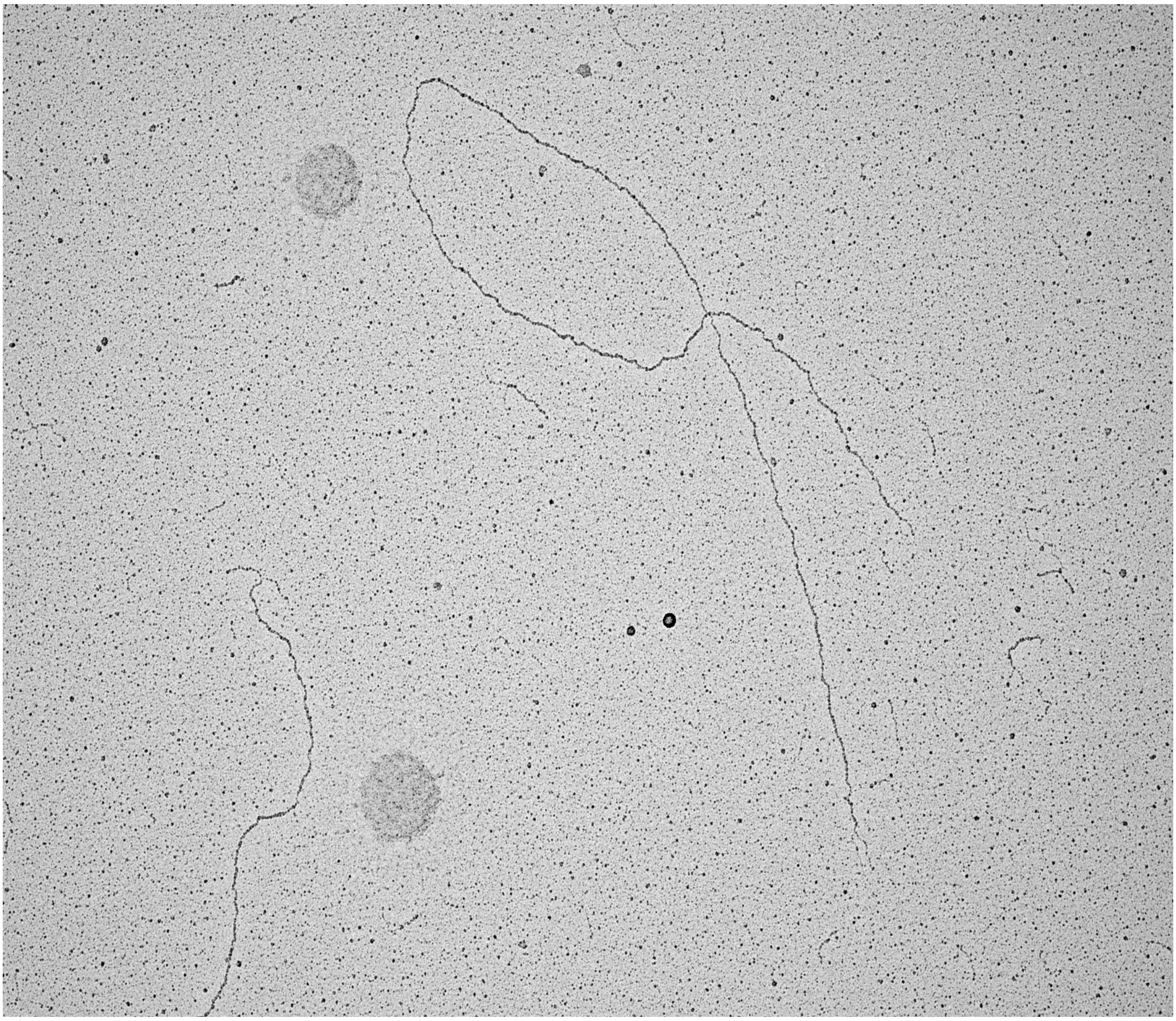

Supplement: Supplementary file 6 — Source Data [file 41467_2020_19139_MOESM6_ESM.zip › Source data 2nd rev/Source data Figure 5.pdf]
