# Supplementary material for: Telomere damage induces internal loops that generate telomeric circles
Source: Nat Commun. 2020 Oct 20;11:5297. doi: 10.1038/s41467-020-19139-4 (PMC7576219; doi:10.1038/s41467-020-19139-4)
Supplement: Supplementary file 6 — Source Data [file 41467_2020_19139_MOESM6_ESM.zip › Source data 2nd rev/Source data Figure 7.pdf]

Figure 7E

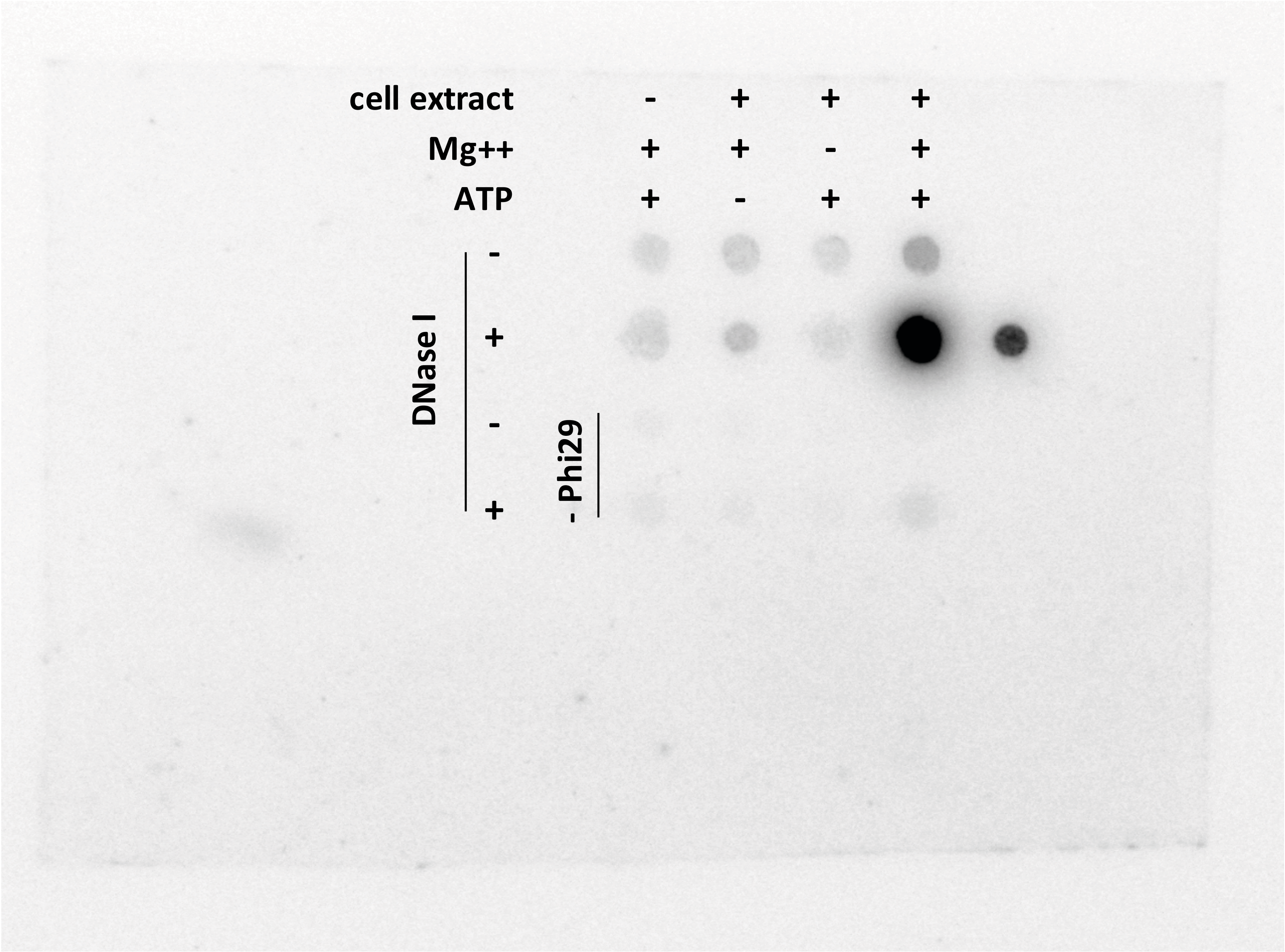

Figure 7F

	C-circle signal (arbitrary units)			
	- DNaseI - extract	+DnaseI -extract	**+DnaseI +extract**	**-DnaseI +extract**
Exp1	1	1.2	**5.03**	**1.39**
Exp2	1	ND	**4.71**	**1.26**
Exp3	1	0.69	**4.51**	**0.56**

The value for column 1 (the control) was set arbitrarily to 1 and the other values were reported relative to column 1
