# Supplementary material for: Telomere damage induces internal loops that generate telomeric circles
Source: Nat Commun. 2020 Oct 20;11:5297. doi: 10.1038/s41467-020-19139-4 (PMC7576219; doi:10.1038/s41467-020-19139-4)

**Supplementary Figure 2a**

| molecule length distribution (kb) | |  |  |  |  |  |  |
| --- | --- | --- | --- | --- | --- | --- | --- |
|  |  |  |  |  |  |  |  |
| 50.5556 | 20.4200 | 15.6944 | 11.5556 | 8.7778 | 7.0556 | 5.6111 | 4.0556 |
| 49.7500 | 20.3333 | 15.6667 | 11.5278 | 8.7500 | 7.0556 | 5.6111 | 4.0556 |
| 49.6667 | 20.3300 | 15.6389 | 11.5278 | 8.7500 | 7.0278 | 5.6111 | 4.0278 |
| 49.5000 | 20.2778 | 15.5833 | 11.5000 | 8.7500 | 7.0278 | 5.5833 | 4.0278 |
| 49.1389 | 20.2778 | 15.5556 | 11.5000 | 8.7500 | 7.0278 | 5.5833 | 4.0278 |
| 47.9167 | 20.2500 | 15.5556 | 11.4167 | 8.7500 | 7.0278 | 5.5600 | 4.0278 |
| 46.4444 | 20.2500 | 15.5278 | 11.3889 | 8.7222 | 7.0000 | 5.5556 | 4.0278 |
| 43.9444 | 20.2500 | 15.5000 | 11.3611 | 8.7222 | 6.9722 | 5.5556 | 4.0000 |
| 43.0833 | 20.1111 | 15.5000 | 11.3333 | 8.6944 | 6.9722 | 5.5556 | 4.0000 |
| 42.5556 | 20.0833 | 15.5000 | 11.3333 | 8.6944 | 6.9444 | 5.5556 | 3.9722 |
| 42.1944 | 20.0833 | 15.4444 | 11.3333 | 8.6944 | 6.9444 | 5.5556 | 3.9722 |
| 42.0833 | 20.0800 | 15.4444 | 11.2778 | 8.6667 | 6.9444 | 5.5556 | 3.9722 |
| 41.1111 | 20.0556 | 15.4400 | 11.2500 | 8.6389 | 6.9444 | 5.5556 | 3.9444 |
| 40.0833 | 20.0556 | 15.4167 | 11.1667 | 8.6111 | 6.9444 | 5.5278 | 3.9444 |
| 39.6944 | 20.0300 | 15.3611 | 11.1111 | 8.6111 | 6.9444 | 5.5000 | 3.9444 |
| 38.9722 | 20.0300 | 15.3333 | 11.1111 | 8.6111 | 6.9167 | 5.5000 | 3.9167 |
| 38.6667 | 20.0000 | 15.3333 | 11.1111 | 8.6111 | 6.9167 | 5.5000 | 3.9167 |
| 37.8333 | 19.9444 | 15.3056 | 11.1111 | 8.5833 | 6.8889 | 5.5000 | 3.8889 |
| 37.7800 | 19.9444 | 15.2778 | 11.0833 | 8.5833 | 6.8889 | 5.4722 | 3.8889 |
| 37.7500 | 19.9200 | 15.2222 | 11.0556 | 8.5556 | 6.8611 | 5.4722 | 3.8889 |
| 37.7500 | 19.8611 | 15.2222 | 11.0556 | 8.5556 | 6.8611 | 5.4722 | 3.8889 |
| 37.5278 | 19.8300 | 15.2222 | 11.0278 | 8.5556 | 6.8611 | 5.4722 | 3.8889 |
| 37.0000 | 19.8056 | 15.1389 | 11.0278 | 8.5300 | 6.8611 | 5.4444 | 3.8611 |
| 36.8333 | 19.8056 | 15.1111 | 11.0278 | 8.5278 | 6.8611 | 5.4444 | 3.8611 |
| 36.5000 | 19.7778 | 15.0833 | 11.0000 | 8.5278 | 6.8333 | 5.4444 | 3.8333 |
| 36.3056 | 19.7778 | 15.0556 | 10.9722 | 8.5278 | 6.8333 | 5.4444 | 3.8333 |
| 36.2200 | 19.7500 | 15.0300 | 10.9722 | 8.5278 | 6.8333 | 5.4444 | 3.8333 |
| 36.1389 | 19.7222 | 15.0278 | 10.9444 | 8.5278 | 6.8333 | 5.4444 | 3.8333 |
| 35.8611 | 19.7222 | 15.0278 | 10.9444 | 8.5000 | 6.8194 | 5.4167 | 3.8333 |
| 35.1667 | 19.6389 | 15.0000 | 10.9444 | 8.4722 | 6.8056 | 5.3889 | 3.8056 |
| 34.1389 | 19.6389 | 15.0000 | 10.9167 | 8.4722 | 6.8056 | 5.3889 | 3.8056 |
| 33.5556 | 19.6389 | 15.0000 | 10.8611 | 8.4722 | 6.8056 | 5.3889 | 3.7778 |
| 33.3333 | 19.5556 | 14.9444 | 10.8056 | 8.4722 | 6.7778 | 5.3889 | 3.7778 |
| 33.3300 | 19.5000 | 14.9444 | 10.7778 | 8.4444 | 6.7500 | 5.3889 | 3.7500 |
| 33.0000 | 19.4444 | 14.9167 | 10.7500 | 8.4444 | 6.7500 | 5.3889 | 3.7500 |
| 32.9722 | 19.3889 | 14.8900 | 10.7222 | 8.4444 | 6.7500 | 5.3889 | 3.7500 |
| 32.6111 | 19.3056 | 14.8889 | 10.7222 | 8.4444 | 6.7500 | 5.3889 | 3.7500 |
| 32.5000 | 19.2778 | 14.8611 | 10.6944 | 8.4167 | 6.7222 | 5.3889 | 3.7222 |
| 32.5000 | 19.2500 | 14.8333 | 10.6944 | 8.4167 | 6.7222 | 5.3611 | 3.7222 |
| 32.5000 | 19.1667 | 14.8333 | 10.6389 | 8.4167 | 6.7222 | 5.3611 | 3.6944 |
| 32.0556 | 19.0833 | 14.8056 | 10.6389 | 8.4167 | 6.6944 | 5.3611 | 3.6667 |
| 32.0556 | 19.0833 | 14.7778 | 10.6389 | 8.4167 | 6.6944 | 5.3333 | 3.6667 |
| 32.0278 | 19.0556 | 14.7222 | 10.6389 | 8.4167 | 6.6944 | 5.3056 | 3.6667 |
| 31.6111 | 19.0000 | 14.6900 | 10.6111 | 8.4167 | 6.6667 | 5.3056 | 3.6667 |
| 31.4167 | 18.9722 | 14.6667 | 10.6111 | 8.3889 | 6.6667 | 5.3056 | 3.6667 |
| 31.0833 | 18.9444 | 14.6667 | 10.5833 | 8.3889 | 6.6389 | 5.3056 | 3.6667 |
| 31.0556 | 18.9167 | 14.6111 | 10.5556 | 8.3889 | 6.6389 | 5.3056 | 3.6389 |
| 30.9444 | 18.8889 | 14.5833 | 10.5278 | 8.3889 | 6.6389 | 5.2800 | 3.6389 |
| 30.8300 | 18.8889 | 14.5556 | 10.5278 | 8.3611 | 6.6111 | 5.2778 | 3.6389 |
| 30.7500 | 18.8889 | 14.5278 | 10.5000 | 8.3333 | 6.6111 | 5.2778 | 3.6111 |
| 30.4444 | 18.8333 | 14.5000 | 10.5000 | 8.3333 | 6.6111 | 5.2778 | 3.6111 |
| 30.4167 | 18.8333 | 14.4722 | 10.4722 | 8.3333 | 6.6111 | 5.2500 | 3.5833 |
| 30.0000 | 18.8333 | 14.4444 | 10.4722 | 8.3333 | 6.6111 | 5.2500 | 3.5833 |
| 29.6389 | 18.8333 | 14.3611 | 10.4722 | 8.3333 | 6.6111 | 5.2500 | 3.5833 |
| 29.4167 | 18.8300 | 14.3611 | 10.4444 | 8.3056 | 6.6111 | 5.2500 | 3.5556 |
| 29.3333 | 18.8100 | 14.3611 | 10.4444 | 8.2778 | 6.5833 | 5.2500 | 3.5556 |
| 29.1667 | 18.8056 | 14.3333 | 10.4444 | 8.2778 | 6.5833 | 5.2500 | 3.5556 |
| 29.1667 | 18.7800 | 14.2778 | 10.3889 | 8.2778 | 6.5833 | 5.2500 | 3.5278 |
| 29.1111 | 18.7778 | 14.2500 | 10.3889 | 8.2500 | 6.5556 | 5.2222 | 3.5278 |
| 29.0833 | 18.7500 | 14.2500 | 10.3611 | 8.2500 | 6.5556 | 5.1944 | 3.5000 |
| 29.0800 | 18.6944 | 14.2500 | 10.3333 | 8.2222 | 6.5556 | 5.1944 | 3.5000 |
| 29.0278 | 18.6667 | 14.2500 | 10.3333 | 8.1944 | 6.5556 | 5.1667 | 3.4722 |
| 28.6900 | 18.6389 | 14.2500 | 10.3333 | 8.1944 | 6.5556 | 5.1667 | 3.4444 |
| 28.6389 | 18.6111 | 14.2500 | 10.3333 | 8.1900 | 6.5278 | 5.1667 | 3.4167 |
| 28.3889 | 18.6111 | 14.2222 | 10.3333 | 8.1667 | 6.5278 | 5.1667 | 3.4167 |
| 28.2778 | 18.6111 | 14.2222 | 10.3333 | 8.1667 | 6.5278 | 5.1389 | 3.3889 |
| 28.1944 | 18.5000 | 14.2222 | 10.3056 | 8.1389 | 6.5278 | 5.1389 | 3.3611 |
| 28.0833 | 18.3889 | 14.1944 | 10.2778 | 8.1389 | 6.5278 | 5.1389 | 3.3611 |
| 28.0278 | 18.3889 | 14.1900 | 10.2500 | 8.1111 | 6.5278 | 5.1111 | 3.3611 |
| 27.9200 | 18.3600 | 14.1667 | 10.2500 | 8.1111 | 6.5278 | 5.1111 | 3.3611 |
| 27.8889 | 18.3333 | 14.1389 | 10.2222 | 8.1111 | 6.5278 | 5.1111 | 3.3333 |
| 27.4167 | 18.3056 | 14.1389 | 10.2222 | 8.1111 | 6.5000 | 5.1111 | 3.3333 |
| 27.1111 | 18.2778 | 14.1389 | 10.1944 | 8.1111 | 6.5000 | 5.1111 | 3.3333 |
| 27.1111 | 18.2778 | 14.1389 | 10.1944 | 8.0833 | 6.5000 | 5.0833 | 3.3056 |
| 27.0278 | 18.2778 | 14.1111 | 10.1944 | 8.0833 | 6.5000 | 5.0833 | 3.3056 |
| 26.9722 | 18.2222 | 14.1111 | 10.1944 | 8.0833 | 6.5000 | 5.0278 | 3.2778 |
| 26.8889 | 18.1944 | 14.0833 | 10.1667 | 8.0556 | 6.4444 | 5.0000 | 3.2778 |
| 26.8056 | 18.1667 | 14.0833 | 10.1389 | 8.0556 | 6.4444 | 5.0000 | 3.2778 |
| 26.7222 | 18.1389 | 14.0800 | 10.1389 | 8.0556 | 6.4444 | 5.0000 | 3.2778 |
| 26.6944 | 18.1389 | 14.0556 | 10.1389 | 8.0278 | 6.4444 | 4.9722 | 3.2778 |
| 26.5833 | 18.1111 | 14.0556 | 10.1389 | 8.0000 | 6.4444 | 4.9722 | 3.2778 |
| 26.4722 | 18.0556 | 14.0300 | 10.1389 | 8.0000 | 6.4444 | 4.9722 | 3.2778 |
| 26.4167 | 18.0556 | 14.0278 | 10.1389 | 8.0000 | 6.4400 | 4.9722 | 3.2778 |
| 26.3611 | 18.0000 | 13.9444 | 10.1389 | 7.9722 | 6.4167 | 4.9722 | 3.2500 |
| 26.1700 | 17.9444 | 13.9444 | 10.1389 | 7.9722 | 6.4167 | 4.9444 | 3.2500 |
| 26.1400 | 17.9167 | 13.9444 | 10.1111 | 7.9722 | 6.4167 | 4.9444 | 3.2500 |
| 26.1111 | 17.8333 | 13.9167 | 10.0833 | 7.9444 | 6.4167 | 4.9167 | 3.2500 |
| 26.0833 | 17.8056 | 13.9167 | 10.0833 | 7.9167 | 6.4167 | 4.9167 | 3.2222 |
| 25.9722 | 17.7800 | 13.8900 | 10.0556 | 7.8900 | 6.4167 | 4.8889 | 3.2222 |
| 25.9200 | 17.7778 | 13.8889 | 10.0556 | 7.8889 | 6.3889 | 4.8889 | 3.1944 |
| 25.6389 | 17.7500 | 13.8889 | 10.0278 | 7.8611 | 6.3889 | 4.8889 | 3.1944 |
| 25.5556 | 17.6667 | 13.8611 | 10.0278 | 7.8611 | 6.3611 | 4.8611 | 3.1944 |
| 25.5556 | 17.6667 | 13.8611 | 10.0000 | 7.8611 | 6.3611 | 4.8333 | 3.1944 |
| 25.4167 | 17.6667 | 13.8611 | 10.0000 | 7.8333 | 6.3611 | 4.8333 | 3.1667 |
| 25.3889 | 17.5833 | 13.8600 | 9.9722 | 7.8333 | 6.3611 | 4.8333 | 3.1667 |
| 25.2500 | 17.5556 | 13.8056 | 9.9722 | 7.8333 | 6.3600 | 4.8056 | 3.1667 |
| 25.2222 | 17.5000 | 13.8056 | 9.9722 | 7.8333 | 6.3333 | 4.8056 | 3.1667 |
| 25.1400 | 17.5000 | 13.7778 | 9.9722 | 7.8333 | 6.3333 | 4.7800 | 3.1667 |
| 25.1111 | 17.5000 | 13.7778 | 9.9444 | 7.8056 | 6.3056 | 4.7778 | 3.1389 |
| 24.9444 | 17.5000 | 13.7500 | 9.9444 | 7.8056 | 6.3056 | 4.7778 | 3.1389 |
| 24.8900 | 17.4722 | 13.6944 | 9.9444 | 7.8056 | 6.3056 | 4.7778 | 3.1389 |
| 24.8900 | 17.4700 | 13.6667 | 9.9167 | 7.7778 | 6.3056 | 4.7778 | 3.1111 |
| 24.8889 | 17.4444 | 13.6389 | 9.9167 | 7.7500 | 6.3056 | 4.7778 | 3.1111 |
| 24.8611 | 17.4444 | 13.6111 | 9.8900 | 7.7500 | 6.3056 | 4.7778 | 3.1111 |
| 24.8611 | 17.3889 | 13.6111 | 9.8333 | 7.7222 | 6.2778 | 4.7778 | 3.1111 |
| 24.8056 | 17.3611 | 13.6111 | 9.8333 | 7.7222 | 6.2778 | 4.7500 | 3.0833 |
| 24.7500 | 17.3056 | 13.5833 | 9.8333 | 7.7222 | 6.2778 | 4.7500 | 3.0556 |
| 24.6667 | 17.3056 | 13.5556 | 9.8056 | 7.7222 | 6.2778 | 4.7222 | 3.0556 |
| 24.6667 | 17.2222 | 13.5300 | 9.7778 | 7.7222 | 6.2500 | 4.7222 | 3.0000 |
| 24.6667 | 17.1667 | 13.5000 | 9.7778 | 7.7222 | 6.2500 | 4.7222 | 2.9722 |
| 24.5600 | 17.1111 | 13.3889 | 9.7528 | 7.7139 | 6.2500 | 4.7222 | 2.9444 |
| 24.5556 | 17.1111 | 13.3611 | 9.7500 | 7.6944 | 6.2500 | 4.7222 | 2.9444 |
| 24.5000 | 17.1100 | 13.3611 | 9.7500 | 7.6944 | 6.2500 | 4.7222 | 2.9167 |
| 24.4167 | 17.0833 | 13.3333 | 9.6667 | 7.6944 | 6.2222 | 4.7222 | 2.8889 |
| 24.3900 | 17.0556 | 13.2800 | 9.6389 | 7.6667 | 6.2222 | 4.7222 | 2.8889 |
| 24.3889 | 17.0556 | 13.2778 | 9.6389 | 7.6667 | 6.2222 | 4.6944 | 2.8611 |
| 24.3611 | 17.0300 | 13.2778 | 9.6389 | 7.6667 | 6.2222 | 4.6900 | 2.8611 |
| 24.2500 | 17.0278 | 13.2222 | 9.6389 | 7.6667 | 6.2222 | 4.6667 | 2.8333 |
| 24.1667 | 17.0278 | 13.1667 | 9.6111 | 7.6389 | 6.1944 | 4.6667 | 2.8333 |
| 24.1389 | 17.0278 | 13.1667 | 9.5833 | 7.6111 | 6.1944 | 4.6667 | 2.8333 |
| 24.0833 | 17.0278 | 13.1111 | 9.5833 | 7.6111 | 6.1667 | 4.6389 | 2.8333 |
| 24.0278 | 17.0278 | 13.0833 | 9.5556 | 7.6111 | 6.1667 | 4.6389 | 2.8333 |
| 24.0000 | 17.0000 | 13.0556 | 9.5556 | 7.6111 | 6.1667 | 4.6389 | 2.8056 |
| 23.9400 | 17.0000 | 13.0556 | 9.5278 | 7.5833 | 6.1667 | 4.6389 | 2.7778 |
| 23.8611 | 16.9722 | 13.0556 | 9.5278 | 7.5556 | 6.1389 | 4.6111 | 2.7778 |
| 23.8611 | 16.9167 | 12.9722 | 9.5278 | 7.5556 | 6.1111 | 4.6111 | 2.7778 |
| 23.8611 | 16.8611 | 12.9722 | 9.5000 | 7.5556 | 6.1111 | 4.6111 | 2.7778 |
| 23.8333 | 16.8611 | 12.9722 | 9.5000 | 7.5556 | 6.1111 | 4.5833 | 2.7500 |
| 23.7778 | 16.8056 | 12.9444 | 9.5000 | 7.5278 | 6.1111 | 4.5833 | 2.7500 |
| 23.6944 | 16.8056 | 12.9167 | 9.5000 | 7.5278 | 6.1100 | 4.5833 | 2.6944 |
| 23.5556 | 16.7778 | 12.8889 | 9.5000 | 7.5278 | 6.0833 | 4.5833 | 2.6900 |
| 23.5278 | 16.7778 | 12.8889 | 9.5000 | 7.5278 | 6.0833 | 4.5556 | 2.6667 |
| 23.4722 | 16.7500 | 12.8611 | 9.4722 | 7.5278 | 6.0556 | 4.5556 | 2.6667 |
| 23.4444 | 16.7222 | 12.8333 | 9.4444 | 7.5000 | 6.0556 | 4.5556 | 2.6667 |
| 23.3333 | 16.6944 | 12.8333 | 9.4444 | 7.5000 | 6.0556 | 4.5278 | 2.6111 |
| 23.3333 | 16.6667 | 12.8333 | 9.4444 | 7.5000 | 6.0556 | 4.5278 | 2.5833 |
| 23.3333 | 16.6667 | 12.8056 | 9.4167 | 7.5000 | 6.0556 | 4.5000 | 2.5833 |
| 23.2200 | 16.6667 | 12.8056 | 9.4167 | 7.4722 | 6.0300 | 4.5000 | 2.5556 |
| 23.1389 | 16.6389 | 12.7500 | 9.3889 | 7.4722 | 6.0300 | 4.5000 | 2.5278 |
| 23.0000 | 16.6111 | 12.7222 | 9.3611 | 7.4444 | 6.0278 | 4.5000 | 2.5278 |
| 22.9167 | 16.5833 | 12.6667 | 9.3611 | 7.4444 | 6.0000 | 4.4722 | 2.5000 |
| 22.9167 | 16.5278 | 12.6389 | 9.3611 | 7.4444 | 6.0000 | 4.4722 | 2.4722 |
| 22.8889 | 16.5000 | 12.6389 | 9.3333 | 7.4444 | 6.0000 | 4.4722 | 2.4444 |
| 22.8056 | 16.5000 | 12.6111 | 9.3056 | 7.4444 | 6.0000 | 4.4722 | 2.4167 |
| 22.8056 | 16.4722 | 12.6111 | 9.3056 | 7.4444 | 5.9722 | 4.4722 | 2.4167 |
| 22.7222 | 16.4444 | 12.6111 | 9.3056 | 7.4167 | 5.9722 | 4.4722 | 2.3889 |
| 22.6667 | 16.4400 | 12.5833 | 9.2778 | 7.4167 | 5.9722 | 4.4444 | 2.3611 |
| 22.6111 | 16.4167 | 12.5833 | 9.2778 | 7.3889 | 5.9722 | 4.4444 | 2.3611 |
| 22.4722 | 16.3900 | 12.5556 | 9.2500 | 7.3611 | 5.9722 | 4.4444 | 2.3333 |
| 22.4444 | 16.3611 | 12.5556 | 9.2500 | 7.3611 | 5.9444 | 4.4444 | 2.3333 |
| 22.3889 | 16.3611 | 12.5556 | 9.2222 | 7.3611 | 5.9444 | 4.4167 | 2.3333 |
| 22.3611 | 16.3600 | 12.5278 | 9.2222 | 7.3611 | 5.9444 | 4.4167 | 2.3056 |
| 22.2800 | 16.2778 | 12.5278 | 9.2222 | 7.3333 | 5.9200 | 4.4167 | 2.3056 |
| 22.2800 | 16.2500 | 12.5000 | 9.1944 | 7.3333 | 5.9167 | 4.4167 | 2.2778 |
| 22.2500 | 16.2500 | 12.4444 | 9.1667 | 7.3333 | 5.9167 | 4.3889 | 2.2500 |
| 22.2222 | 16.2500 | 12.3889 | 9.1667 | 7.3056 | 5.9167 | 4.3611 | 2.2500 |
| 22.1667 | 16.2200 | 12.3889 | 9.1389 | 7.3056 | 5.9167 | 4.3611 | 2.2500 |
| 22.1389 | 16.1944 | 12.3889 | 9.1389 | 7.3056 | 5.9167 | 4.3611 | 2.2500 |
| 22.0278 | 16.1900 | 12.3611 | 9.1389 | 7.2800 | 5.9167 | 4.3611 | 2.2500 |
| 21.9444 | 16.1700 | 12.3611 | 9.1389 | 7.2778 | 5.8889 | 4.3333 | 2.2500 |
| 21.9167 | 16.1667 | 12.1389 | 9.1111 | 7.2778 | 5.8889 | 4.3333 | 2.2222 |
| 21.8889 | 16.1667 | 12.1389 | 9.0833 | 7.2778 | 5.8889 | 4.3333 | 2.2222 |
| 21.8611 | 16.1667 | 12.1111 | 9.0833 | 7.2500 | 5.8889 | 4.3056 | 2.1944 |
| 21.7222 | 16.1389 | 12.0833 | 9.0556 | 7.2500 | 5.8611 | 4.3056 | 2.1667 |
| 21.6944 | 16.0833 | 12.0833 | 9.0556 | 7.2500 | 5.8611 | 4.3056 | 2.1667 |
| 21.6389 | 16.0800 | 12.0833 | 9.0556 | 7.2500 | 5.8611 | 4.3056 | 2.1389 |
| 21.6389 | 16.0556 | 12.0556 | 9.0556 | 7.2222 | 5.8611 | 4.3056 | 2.0833 |
| 21.6389 | 16.0556 | 12.0278 | 9.0278 | 7.2222 | 5.8333 | 4.3056 | 1.9722 |
| 21.5000 | 16.0278 | 12.0000 | 9.0278 | 7.2222 | 5.8333 | 4.3056 | 1.9444 |
| 21.5000 | 16.0278 | 12.0000 | 9.0278 | 7.2222 | 5.8056 | 4.3056 | 1.9167 |
| 21.5000 | 16.0000 | 11.9722 | 9.0000 | 7.2222 | 5.8056 | 4.3056 | 1.8056 |
| 21.4167 | 16.0000 | 11.9722 | 9.0000 | 7.1944 | 5.8056 | 4.2778 | 1.7500 |
| 21.2778 | 16.0000 | 11.9444 | 9.0000 | 7.1944 | 5.8056 | 4.2778 | 1.7500 |
| 21.2500 | 15.9722 | 11.8333 | 8.9722 | 7.1944 | 5.7778 | 4.2500 | 1.6944 |
| 21.2222 | 15.9722 | 11.8333 | 8.9722 | 7.1944 | 5.7778 | 4.2500 | 1.6389 |
| 21.2222 | 15.9700 | 11.8333 | 8.9722 | 7.1944 | 5.7778 | 4.2500 | 1.6111 |
| 21.1389 | 15.9444 | 11.8056 | 8.9722 | 7.1667 | 5.7778 | 4.2500 | 1.4722 |
| 20.9444 | 15.9444 | 11.7778 | 8.9444 | 7.1667 | 5.7500 | 4.2500 | 1.3889 |
| 20.9444 | 15.9167 | 11.7778 | 8.9444 | 7.1389 | 5.7500 | 4.2500 | 1.1667 |
| 20.9167 | 15.8889 | 11.7778 | 8.9167 | 7.1389 | 5.7500 | 4.2500 |  |
| 20.9167 | 15.8611 | 11.7778 | 8.8889 | 7.1389 | 5.7222 | 4.2222 |  |
| 20.8900 | 15.8333 | 11.7500 | 8.8889 | 7.1111 | 5.7222 | 4.2222 |  |
| 20.8889 | 15.7778 | 11.7500 | 8.8889 | 7.1111 | 5.7222 | 4.1944 |  |
| 20.8889 | 15.7778 | 11.7222 | 8.8611 | 7.1111 | 5.6944 | 4.1944 |  |
| 20.8300 | 15.7778 | 11.6667 | 8.8333 | 7.0833 | 5.6667 | 4.1944 |  |
| 20.7222 | 15.7778 | 11.6389 | 8.8333 | 7.0833 | 5.6667 | 4.1944 |  |
| 20.6667 | 15.7500 | 11.6111 | 8.8333 | 7.0833 | 5.6667 | 4.1944 |  |
| 20.6667 | 15.7500 | 11.6111 | 8.8333 | 7.0833 | 5.6667 | 4.1667 |  |
| 20.6389 | 15.7500 | 11.5833 | 8.8333 | 7.0833 | 5.6389 | 4.1389 |  |
| 20.6389 | 15.7500 | 11.5833 | 8.8056 | 7.0556 | 5.6111 | 4.0833 |  |
| 20.5556 | 15.7500 | 11.5556 | 8.7778 | 7.0556 | 5.6111 | 4.0833 |  |

Supplementary Figure 2b

|  |  | N (molecules counted) | | % t-loops |
| --- | --- | --- | --- | --- |
| telomere-enriched | Exp1 | 310 |  | 6.77 |
| telomere-enriched | Exp2 | 715 |  | 4.2 |
| telomere-enriched | Exp3 | 845 |  | 4.14 |
|  |  |  |  |  |
| Bulk | Exp1 | 1466 |  | 0.48 |
| Bulk | Exp2 | 776 |  | 0.39 |
| Bulk | Exp3 | 1776 |  | 1.13 |

Supplementary Figure 2c


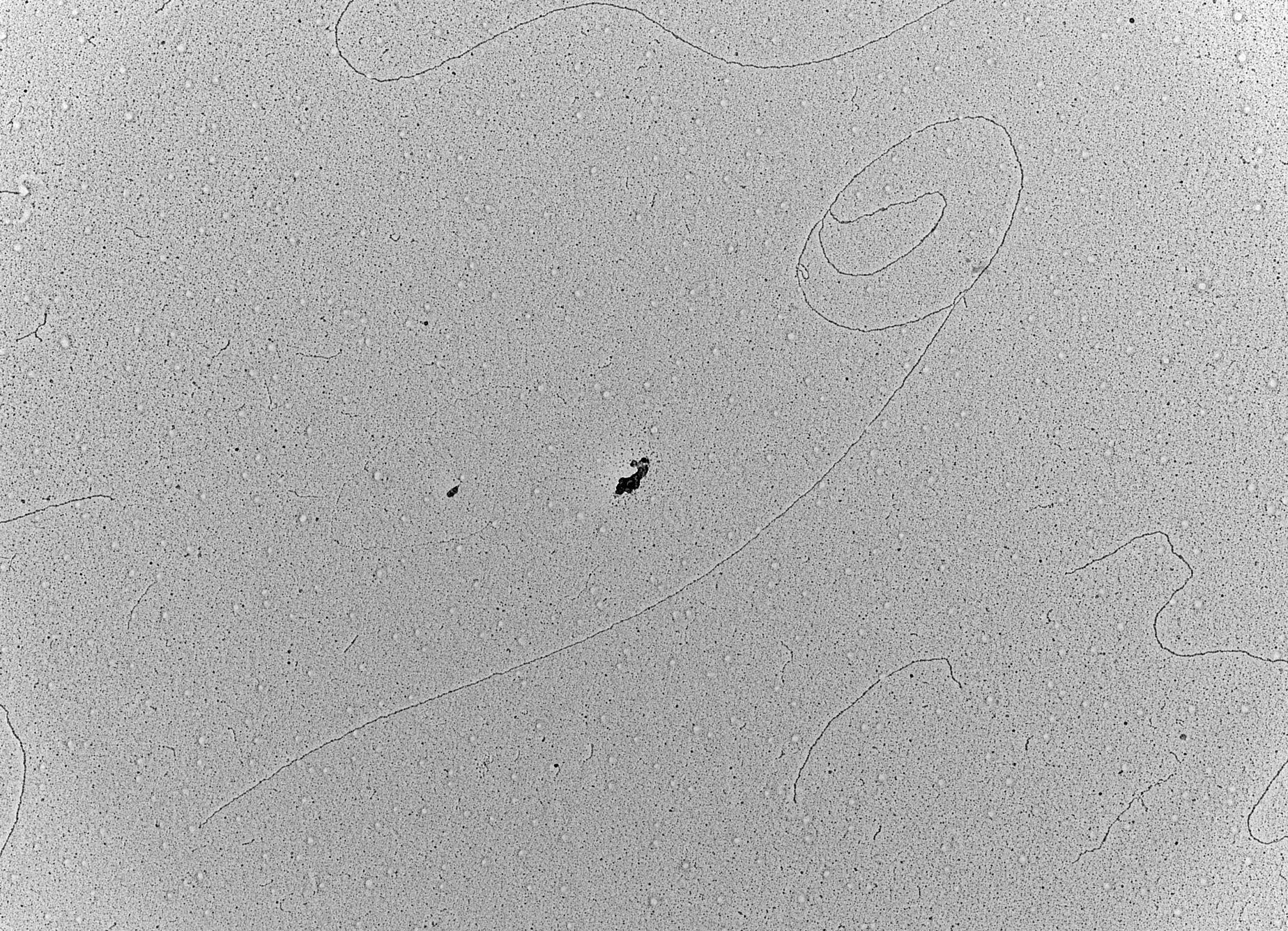

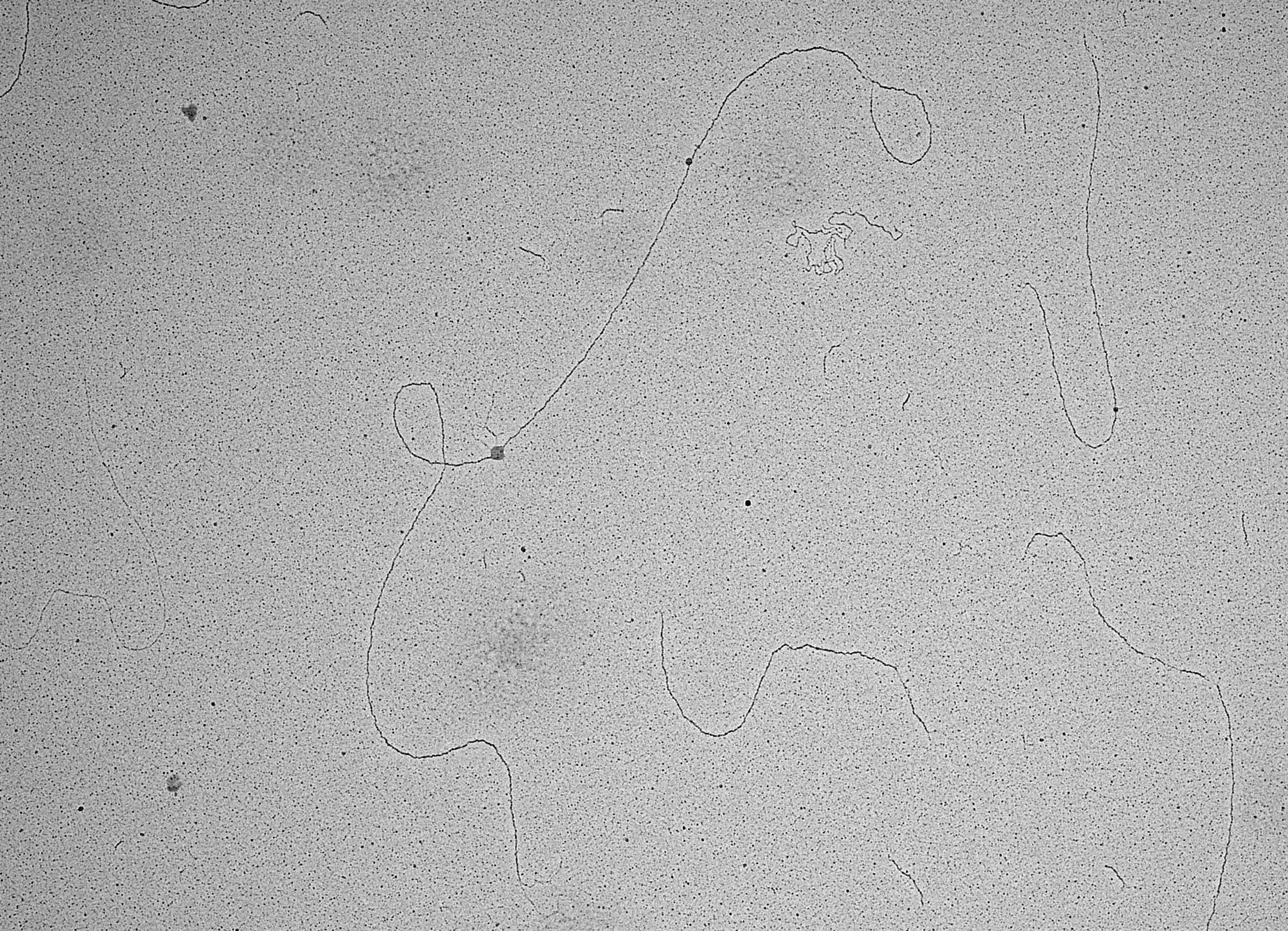

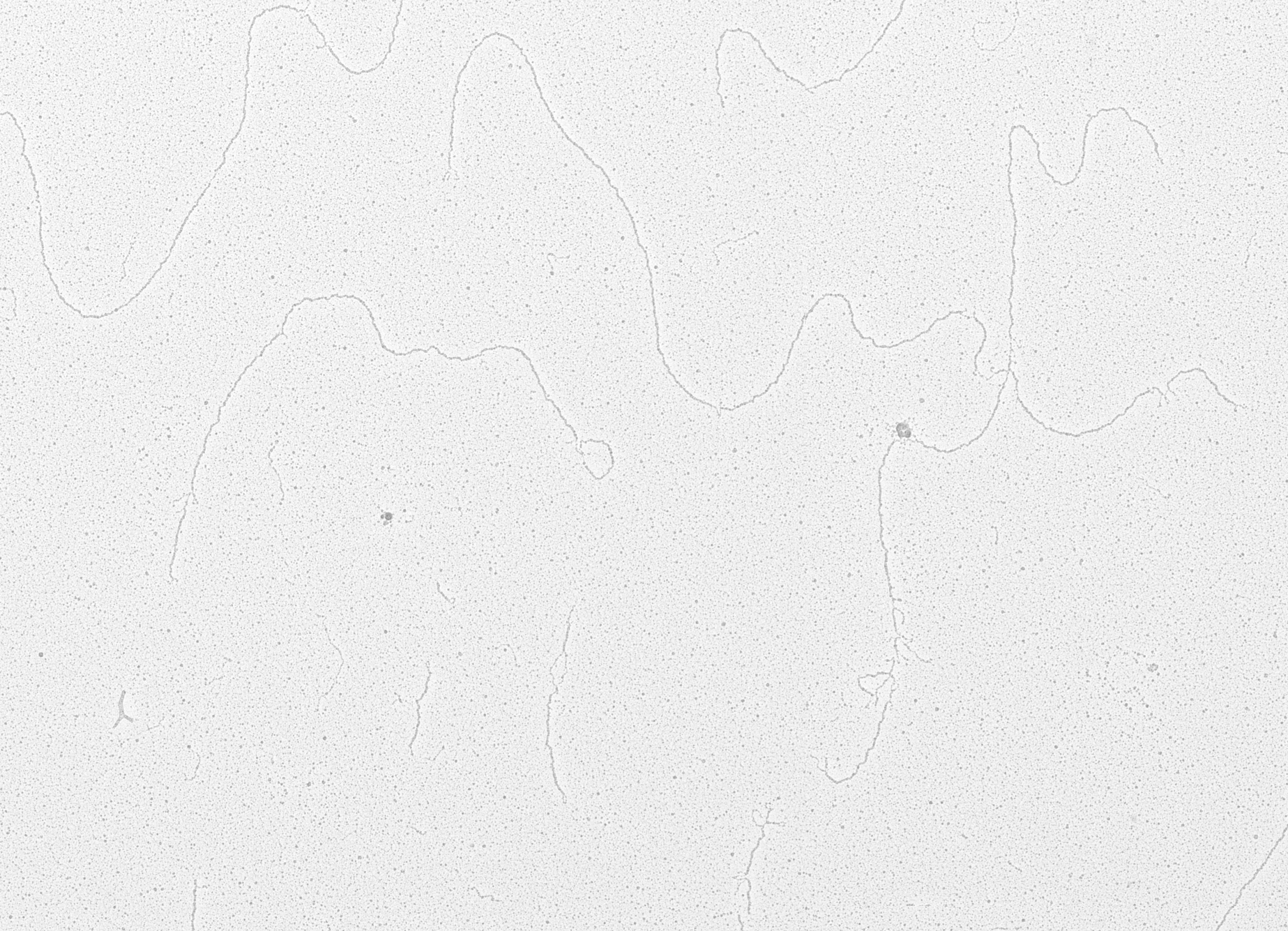

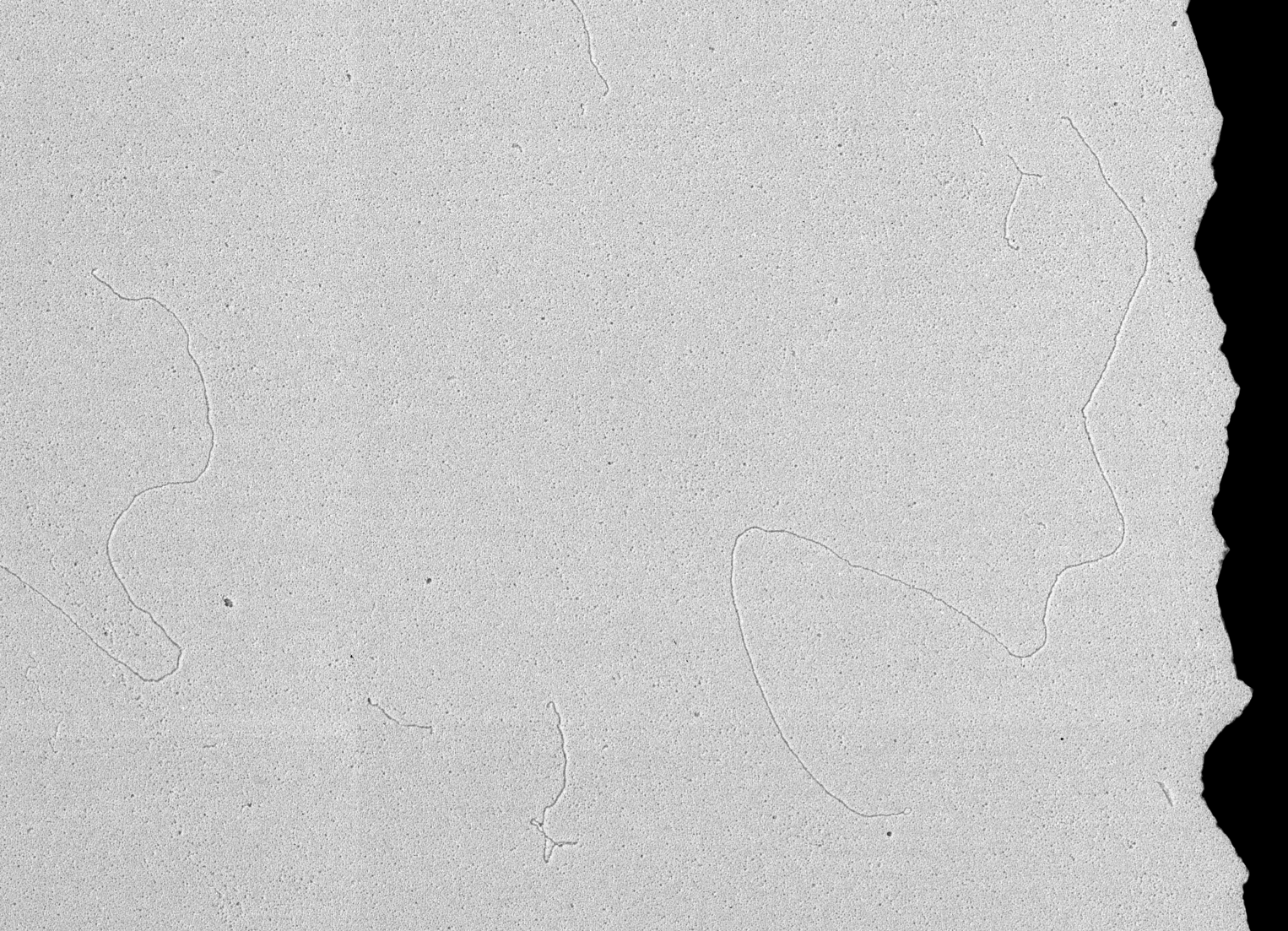

Supplement: Supplementary file 6 — Source Data [file 41467_2020_19139_MOESM6_ESM.zip › Source data 2nd rev/Source data Supplementary Figure 2.docx]
