# Supplementary material for: Telomere damage induces internal loops that generate telomeric circles
Source: Nat Commun. 2020 Oct 20;11:5297. doi: 10.1038/s41467-020-19139-4 (PMC7576219; doi:10.1038/s41467-020-19139-4)
Supplement: Supplementary file 6 — Source Data [file 41467_2020_19139_MOESM6_ESM.zip › Source data 2nd rev/Source data Supplementary Figure 4.docx]

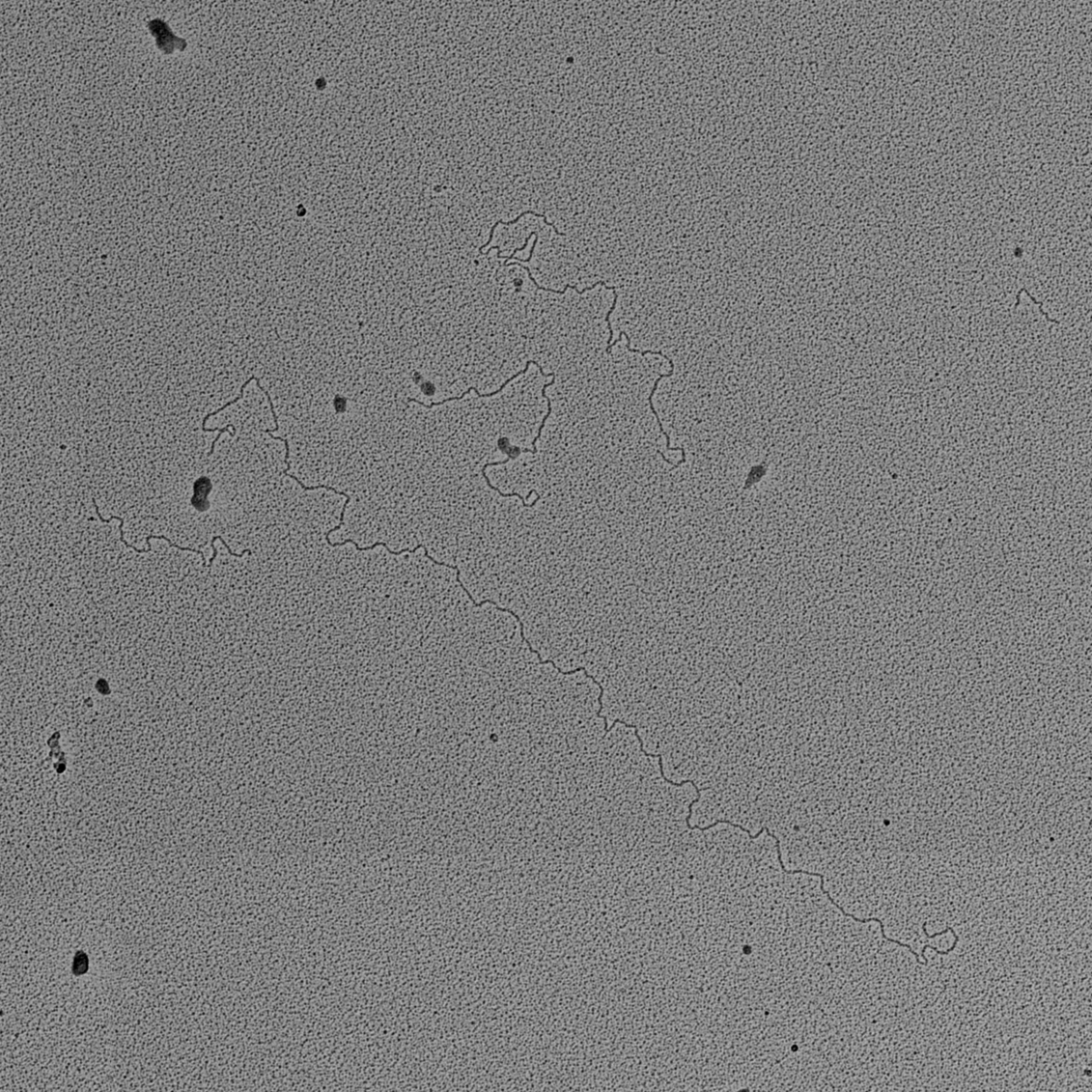
Supplementary Figure 4a


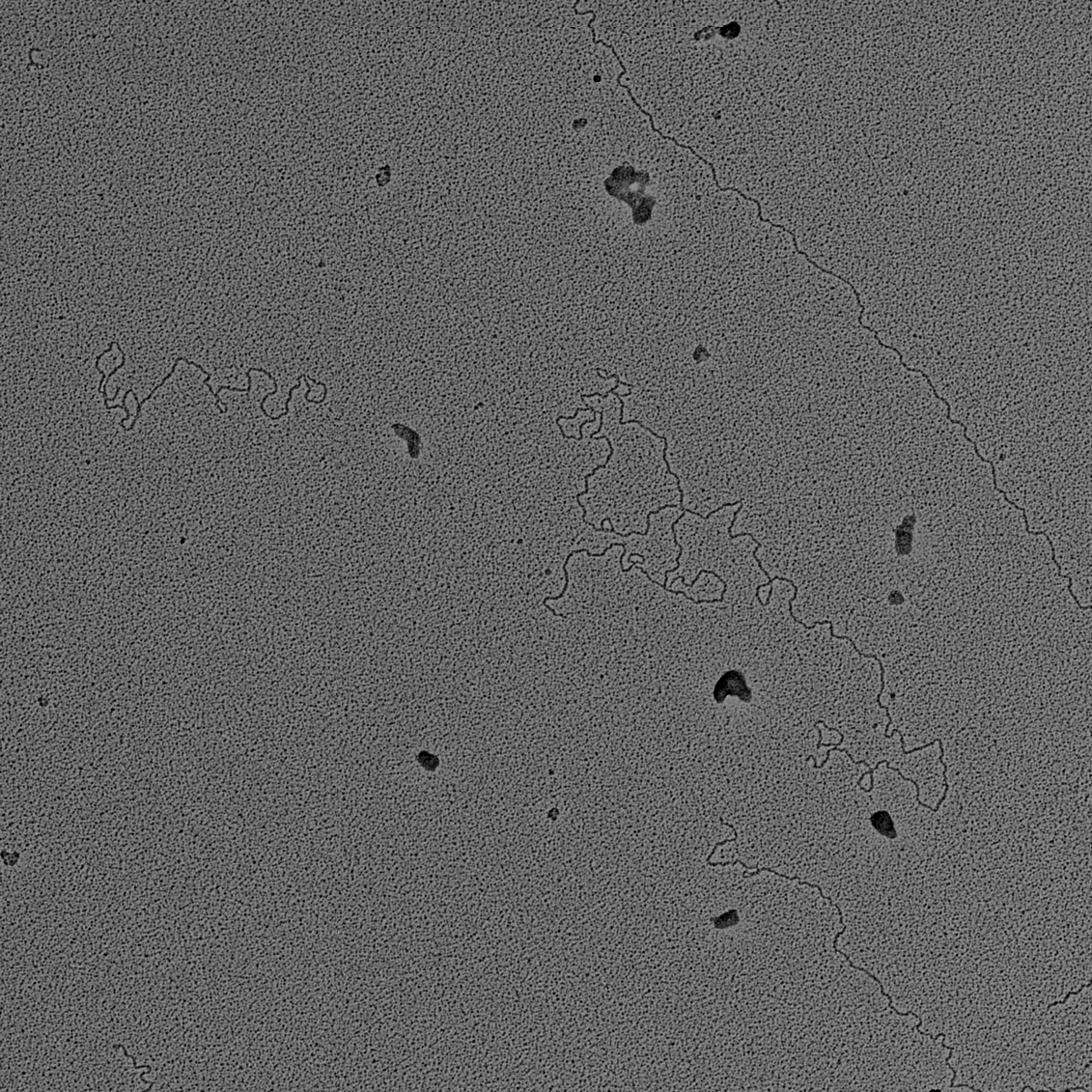

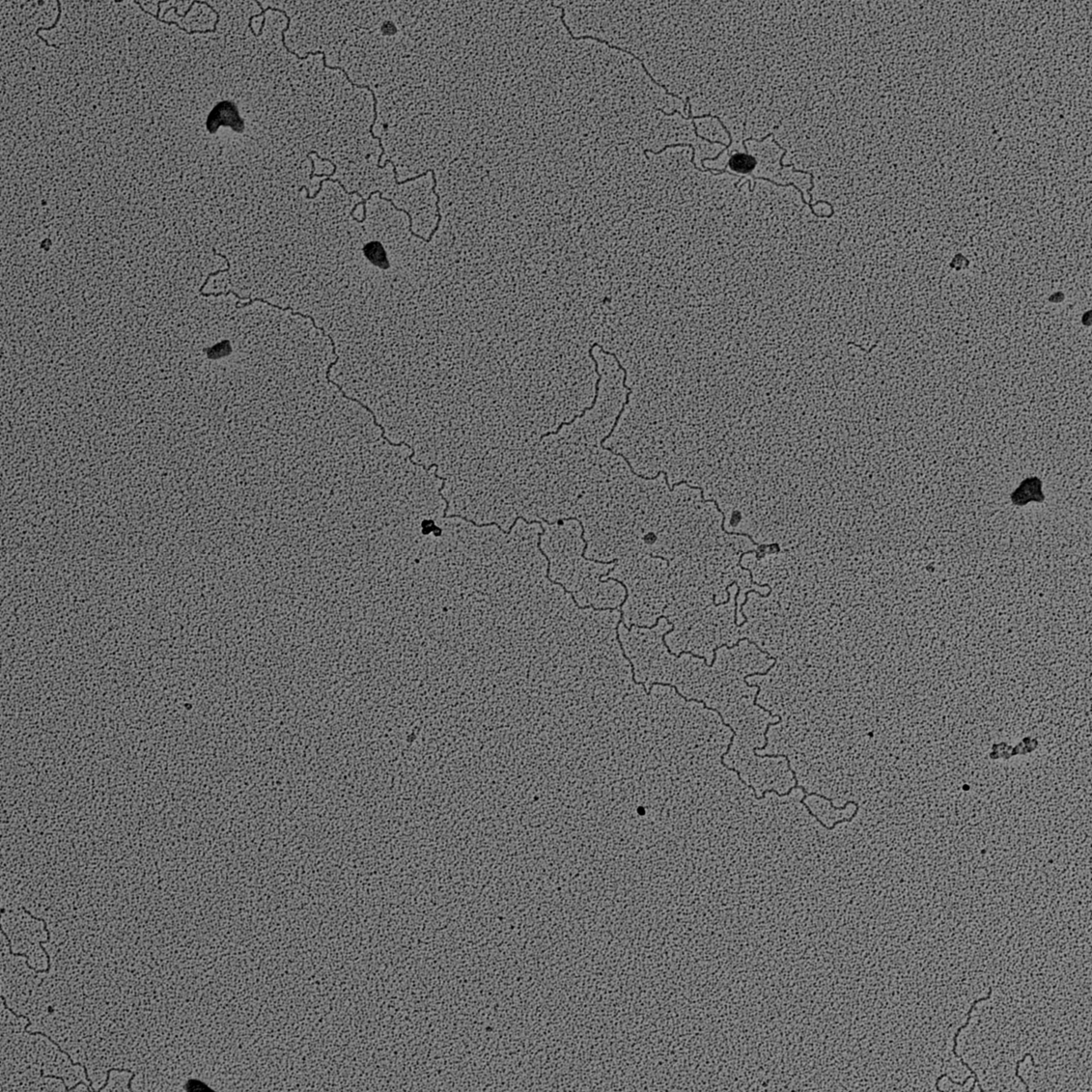


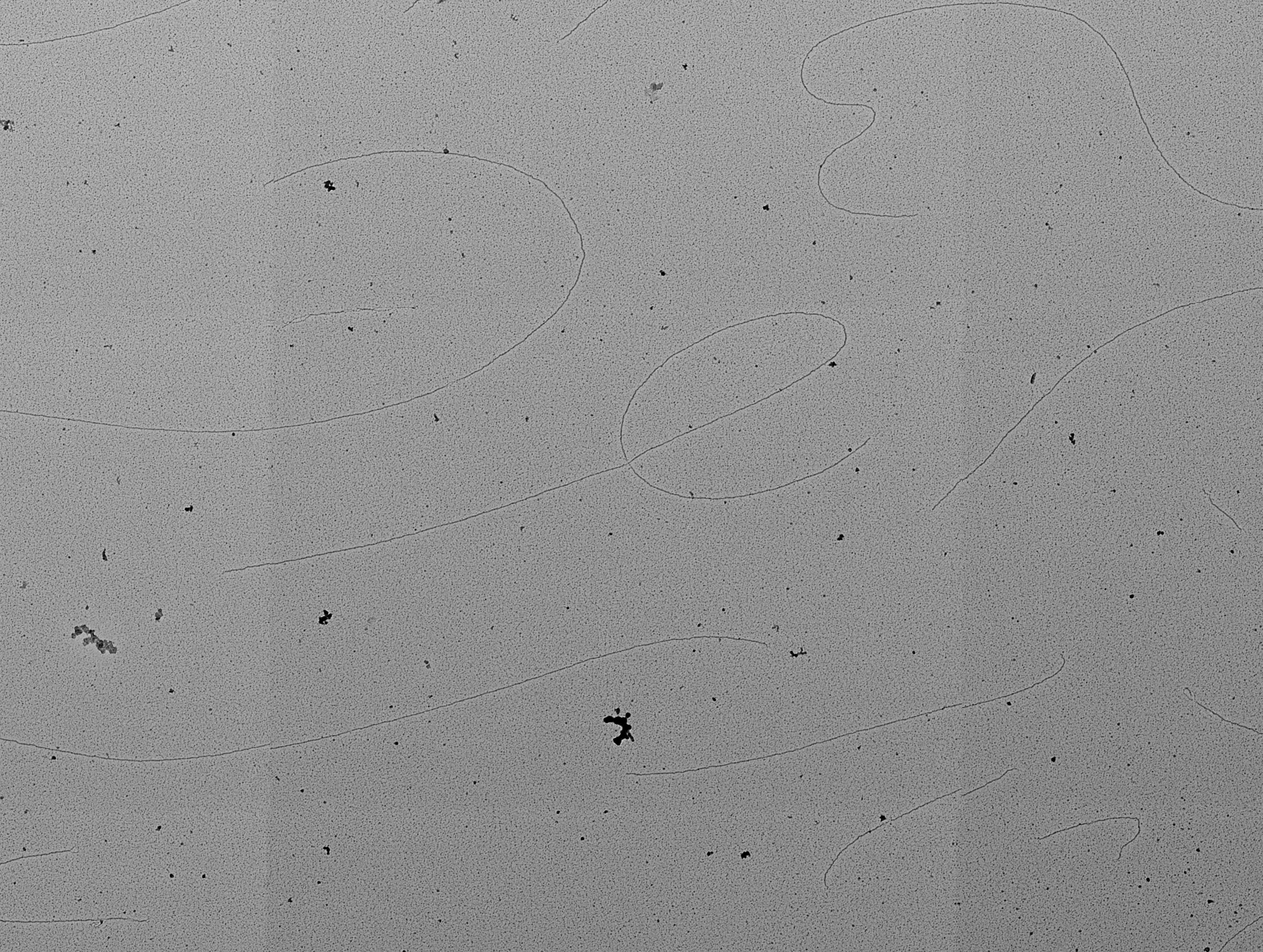
Supplementary Figure 4b

Supplementary Figure 4c

| number of i-loops per kb |  |  |  |
| --- | --- | --- | --- |
| Bulk (Kleinschmidt) | Telomere-enriched (Kleinschmidt) | Bulk (BAC) | Telomere-enriched (BAC) |
| 0.02205456 | 0.01665206 | 0.02846975 | 0.01436552 |
| 0.20652174 | 0.28657617 | 0.01559117 | 0 |
| 0.02328431 | 0.35385411 | 0.05023256 | 0 |
| 0.20119126 | 0.08602151 | 0 | 0 |
| 0.16888889 | 0.25738916 | 0 | 0 |
| 0.02961808 | 0.21482412 | 0.02233251 | 0 |
| 0 | 0.29726206 | 0 | 0 |
| 0 | 0.15405405 | 0 | 0.01773399 |
| 0.09860142 | 0.2472885 | 0 | 0.03658537 |
| 0.07231208 | 0.14393939 | 0.02926829 | 0.0371134 |
| 0.07328833 | 0.53271028 | 0.02977667 | 0.01874024 |
| 0.11875 | 0.36833603 | 0 | 0 |
| 0.08472687 | 0.06163828 | 0 | 0 |
| 0 | 0.37288136 | 0.03296703 | 0 |
| 0.18095238 | 0.2080292 | 0 | 0 |
| 0.04572804 | 0.56769374 | 0 | 0 |
| 0.04583836 | 0.07378641 | 0 | 0 |
| 0.41605839 | 0.48574238 | 0 | 0 |
| 0 | 0.11309524 | 0 | 0 |
| 0 | 0.15245737 | 0 | 0 |
| 0.14357683 | 0.34475806 | 0 | 0.09090909 |
| 0.04878049 | 0.46341463 | 0 | 0 |
| 0 | 0.475 | 0 | 0 |
| 0.1005291 | 0.4 | 0 | 0.02403204 |
| 0.15616438 | 0.44186047 | 0 | 0.02486188 |
| 0 | 0.53175457 | 0 | 0 |
| 0 | 0.37214363 | 0 | 0 |
| 0.11309524 | 0.16648412 | 0 | 0 |
| 0.11326379 | 0.42175361 | 0 | 0 |
| 0 | 0.38255034 | 0 | 0 |
| 0.05714286 | 0.64261556 | 0 | 0 |
| 0 | 0.18160096 | 0 | 0.0258435 |
| 0.17952756 | 0.50789793 | 0 | 0 |
| 0.06031746 | 0.27771011 | 0.04904632 | 0 |
| 0.12140575 | 0.51351351 | 0 | 0 |
| 0.18688525 | 0.14321608 | 0 | 0 |
| 0 | 0.29343629 | 0 | 0 |
| 0.06354515 | 0.49032258 | 0 | 0 |
| 0 | 0.49868766 | 0 | 0 |
| 0.06484642 | 0.70838881 | 0 | 0 |
| 0.13103448 | 0.152 | 0 | 0.02985075 |
| 0.06713781 | 0.41304348 | 0 | 0 |
| 0.13475177 | 0.56870748 | 0 | 0 |
| 0.06761566 | 0.32386364 | 0 | 0 |
| 0 | 0.16618076 | 0 | 0 |
| 0.13571429 | 0.49927007 | 0 | 0 |
| 0.06934307 | 0.33431085 | 0 | 0 |
| 0.06946984 | 0.50220264 | 0 | 0 |
| 0 | 0.61470588 | 0 | 0 |
| 0.1447619 | 0.44837758 | 0 | 0 |
| 0.07321773 | 0.45508982 | 0 | 0 |
| 0 | 0.17065868 | 0 | 0 |
| 0.14643545 | 0.2992126 | 0 | 0 |
| 0 | 0.24012638 | 0 | 0 |
| 0.07465619 | 0.60897436 | 0 | 0 |
| 0.22485207 | 0.81518152 | 0 | 0 |
| 0.07676768 | 0.25165563 | 0 | 0 |
| 0 | 0.69320066 | 0 | 0 |
| 0.15670103 | 0.25375626 | 0 | 0 |
| 0.16450216 | 0.57575758 | 0 | 0 |
| 0 | 0.38841567 | 0 | 0 |
| 0.08351648 | 0.45626072 | 0 | 0 |
| 0.08425721 | 0.39583333 | 0 | 0 |
| 0.0875576 | 0.72569444 | 0 | 0 |
| 0.26511628 | 0.53146853 | 0 | 0 |
| 0 | 0.40070299 | 0 | 0 |
| 0.27078385 | 0.26713533 | 0 | 0 |
| 0.09200969 | 0.47841727 | 0 | 0 |
| 0.46116505 | 0.62068966 | 0 | 0 |
| 0.18627451 | 0.06934307 | 0 | 0 |
| 0 | 0.27992634 | 0 | 0 |
| 0.09405941 | 0.49077491 | 0 | 0 |
| 0.18952618 | 0.1423221 | 0 | 0 |
| 0.19 | 0.21348315 | 0 | 0 |
| 0.19 | 0.43678161 | 0 | 0.04260355 |
| 0.0952381 | 0.43846154 | 0 | 0.04384896 |
| 0.09547739 | 0.22485207 | 0 | 0 |
| 0.38481013 | 0.38383838 | 0 | 0 |
| 0 | 0.23123732 | 0 | 0 |
| 0 | 0.15510204 | 0 | 0 |
| 0 | 0.15510204 | 0 | 0 |
| 0.10026385 | 0.62040816 | 0 | 0 |
| 0.10215054 | 0.31083845 | 0 | 0 |
| 0.20540541 | 0.54508197 | 0 | 0 |
| 0.52054795 | 0.08085106 | 0 | 0 |
| 0.1043956 | 0.3254818 | 0 | 0 |
| 0.1046832 | 0.3254818 | 0 | 0 |
| 0 | 0.51818182 | 0 | 0.04761905 |
| 0.10614525 | 0.45346062 | 0 | 0 |
| 0 | 0.09069212 | 0 | 0 |
| 0.11046512 | 0.46004843 | 0 | 0 |
| 0 | 0.18401937 | 0 | 0.04851752 |
| 0.11411411 | 0.37163814 | 0 | 0.04945055 |
| 0 | 0.28287841 | 0 | 0 |
| 0 | 0.18952618 | 0 | 0 |
| 0.11949686 | 0.67341772 | 0 | 0 |
| 0 | 0.3887468 | 0 | 0.05590062 |
| 0 | 0.69090909 | 0 | 0 |
| 0.24437299 | 0.39480519 | 0 | 0 |
| 0 | 0.2961039 | 0 | 0 |
| 0 | 0.19791667 | 0 | 0 |
| 0.13103448 | 0.29765013 | 0 | 0 |
| 0 | 0.19895288 | 0 | 0 |
| 0.13620072 | 0.6 | 0 | 0 |
| 0.41007194 | 0.1 | 0 | 0.11803279 |
| 0.13818182 | 0.7037037 | 0 | 0 |
| 0.27636364 | 0.20159151 | 0 | 0 |
| 0 | 0.4075067 | 0 | 0 |
| 0 | 0.30978261 | 0 | 0.05980066 |
| 0.28679245 | 0.31147541 | 0 | 0.06101695 |
| 0.29007634 | 0.20821918 | 0 | 0 |
| 0.1484375 | 0.52631579 | 0 | 0.12456747 |
| 0.4453125 | 0.84679666 | 0 | 0 |
| 0.304 | 0.21169916 | 0 | 0 |
| 0 | 0.10704225 | 0 | 0 |
| 0.15261044 | 0.75783476 | 0 | 0 |
| 0.30645161 | 0.54755043 | 0 | 0 |
| 0.15510204 | 0.65706052 | 0 | 0 |
| 0.31147541 | 0.4457478 | 0 | 0 |
| 0.15702479 | 0.33529412 | 0 | 0 |
| 0.16033755 | 0.56047198 | 0 | 0 |
| 0 | 0.4592145 | 0 | 0 |
| 0 | 0.34441088 | 0 | 0 |
| 0 | 0.5775076 | 0 | 0 |
| 0.3438914 | 0.46625767 | 0 | 0 |
| 0 | 0.70153846 | 0 | 0 |
| 0.17431193 | 0.46769231 | 0 | 0 |
| 0.34862385 | 0.47204969 | 0 | 0.07157058 |
| 0.17674419 | 0.94409938 | 0 | 0 |
| 0.17757009 | 0.11875 | 0 | 0 |
| 0.17757009 | 0.35849057 | 0 | 0 |
| 0 | 0.48253968 | 0 | 0 |
| 0.18181818 | 0.6031746 | 0 | 0 |
| 0.36714976 | 0.12063492 | 0 | 0 |
| 0.18627451 | 0.60702875 | 0 | 0.0746888 |
| 0 | 0 | 0 | 0 |
| 0.38383838 | 0.24437299 | 0 | 0 |
| 0.19191919 | 0.36774194 | 0 | 0.07675906 |
| 0.19191919 | 0.61290323 | 0 | 0 |
| 0.3857868 | 0.61889251 | 0 | 0 |
| 0.2 | 0.62091503 | 0 | 0 |
| 0 | 0.24918033 | 0 | 0.07982262 |
| 0.20765027 | 0.62706271 | 0 | 0 |
| 0.21468927 | 0.50498339 | 0 | 0 |
| 0 | 0.25249169 | 0 | 0 |
| 0 | 0.3812709 | 0 | 0 |
| 0 | 0.51178451 | 0 | 0 |
| 0 | 0.63973064 | 0 | 0 |
| 0.23030303 | 0.8956229 | 0 | 0 |
| 0.23170732 | 0.25675676 | 0 | 0 |
| 0.23170732 | 1.02702703 | 0 | 0 |
| 0.23899371 | 0.3890785 | 0 | 0.16666667 |
| 0 | 0.64846416 | 0 | 0 |
| 0 | 0.25938567 | 0 | 0 |
| 0 | 0.3890785 | 0 | 0 |
| 0 | 0.52413793 | 0 | 0 |
| 0.25 | 0.26573427 | 0 | 0 |
| 0.25503356 | 0.4 | 0 | 0 |
| 0.25503356 | 0.53710247 | 0 | 0 |
| 0 | 0.26855124 | 0 | 0 |
| 0 | 0.67137809 | 0 | 0.08759124 |
| 0 | 0.40282686 | 0 | 0 |
| 0 | 0.27142857 | 0 | 0 |
| 0.27737226 | 0.68345324 | 0 | 0 |
| 0 | 0 | 0 | 0 |
| 0 | 0.55272727 | 0 | 0 |
| 0 | 0.41605839 | 0 | 0 |
| 0 | 0.55677656 | 0 | 0 |
| 0 | 0.56296296 | 0 | 0 |
| 0 | 0.42537313 | 0 | 0 |
| 0.32758621 | 0.28464419 | 0 | 0 |
| 0.34234234 | 0.1423221 | 0 | 0 |
| 0.35514019 | 0.42857143 | 0 | 0 |
| 0 | 0.28787879 | 0 | 0.0972973 |
| 0 | 0.14559387 | 0 | 0 |
| 0 | 0.44015444 | 0 | 0.09809264 |
| 0.38383838 | 0.73929961 | 0 | 0 |
| 0 | 0.1484375 | 0 | 0 |
| 0.41304348 | 0.4453125 | 0 | 0 |
| 0 | 0.3015873 | 0 | 0 |
| 0 | 0.60557769 | 0 | 0 |
| 0.42222222 | 0.15384615 | 0 | 0.10084034 |
| 0.42696629 | 0.46153846 | 0 | 0 |
| 0.43181818 | 0.46530612 | 0 | 0 |
| 0 | 0.46721311 | 0 | 0 |
| 0 | 0.31147541 | 0 | 0 |
| 0 | 0.31404959 | 0 | 0 |
| 0 | 0.31666667 | 0 | 0 |
| 0 | 0.16170213 | 0 | 0 |
| 0 | 0.16239316 | 0 | 0 |
| 0 | 0.65800866 | 0 | 0 |
| 0 | 1.15652174 | 0 | 0 |
| 0 | 0.50220264 | 0 | 0 |
| 0 | 0.50666667 | 0 | 0 |
|  | 0.68161435 | 0 | 0 |
|  | 0.17117117 | 0 | 0 |
|  | 0.34545455 | 0 | 0 |
|  | 0.52054795 | 0 | 0 |
|  | 0.34862385 | 0 | 0 |
|  | 0.35348837 | 0 | 0 |
|  | 0.53271028 | 0 | 0 |
|  | 0 | 0 | 0 |
|  | 0.53773585 | 0 | 0 |
|  | 0.18269231 | 0 | 0 |
|  | 0.37438424 | 0 | 0 |
|  | 0.38 | 0 | 0 |
|  | 0.38383838 | 0 | 0 |
|  | 0 | 0 | 0 |
|  | 0.4 | 0 | 0.11042945 |
|  | 0.40425532 | 0 | 0 |
|  | 0.40641711 | 0 | 0 |
|  | 0.41304348 | 0 | 0 |
|  | 0.4198895 | 0 | 0 |
|  | 0.42458101 | 0 | 0 |
|  | 0.2122905 | 0 | 0 |
|  | 0 | 0 | 0 |
|  | 0.21468927 | 0 | 0 |
|  | 0.43181818 | 0 | 0 |
|  | 0.43428571 | 0 | 0 |
|  | 0 | 0 | 0 |
|  | 0.43678161 | 0 | 0 |
|  | 0.65895954 | 0 | 0 |
|  | 0.23170732 | 0 | 0 |
|  | 0.4691358 | 0 | 0 |
|  | 0.24836601 | 0 | 0 |
|  | 0.25165563 | 0 | 0 |
|  | 0 | 0 | 0.12 |
|  | 0.25503356 | 0 | 0 |
|  | 0.25503356 | 0 | 0 |
|  | 1.02702703 | 0 | 0 |
|  | 0 | 0 | 0 |
|  | 0.25675676 | 0 | 0 |
|  | 0.7755102 | 0 | 0 |
|  | 0.26027397 | 0 | 0 |
|  | 0 | 0 | 0.12324546 |
|  | 0.26760563 | 0 | 0 |
|  | 0.54285714 | 0 | 0 |
|  | 0.27737226 | 0 | 0 |
|  | 0 | 0 | 0 |
|  | 0.29457364 | 0 | 0.25174825 |
|  | 0.59375 | 0 | 0 |
|  | 0 | 0 | 0 |
|  | 0.30645161 | 0 | 0 |
|  | 0.31147541 | 0 | 0.25622776 |
|  | 0 | 0 | 0.12811388 |
|  | 0.31932773 | 0 | 0 |
|  | 0 | 0 | 0 |
|  | 0 | 0 | 0 |
|  | 0 | 0 | 0 |
|  | 0 | 0 | 0 |
|  | 0 | 0 | 0 |
|  | 0 | 0 | 0 |
|  | 0.86363636 | 0 | 0 |
|  |  | 0 | 0 |
|  |  | 0 | 0 |
|  |  | 0 | 0 |
|  |  | 0 | 0 |
|  |  | 0 | 0 |
|  |  | 0 | 0 |
|  |  | 0 | 0 |
|  |  | 0 | 0 |
|  |  | 0 | 0.13899614 |
|  |  | 0 | 0 |
|  |  | 0 | 0.14007782 |
|  |  | 0 | 0 |
|  |  |  | 0 |
|  |  |  | 0 |
|  |  |  | 0 |
|  |  |  | 0 |
|  |  |  | 0 |
|  |  |  | 0 |
|  |  |  | 0 |
|  |  |  | 0 |
|  |  |  | 0 |
|  |  |  | 0 |
|  |  |  | 0 |
|  |  |  | 0 |
|  |  |  | 0.14876033 |
|  |  |  | 0 |
|  |  |  | 0 |
|  |  |  | 0 |
|  |  |  | 0 |
|  |  |  | 0 |
|  |  |  | 0 |
|  |  |  | 0 |
|  |  |  | 0 |
|  |  |  | 0 |
|  |  |  | 0 |
|  |  |  | 0 |
|  |  |  | 0 |
|  |  |  | 0 |
|  |  |  | 0 |
|  |  |  | 0 |
|  |  |  | 0 |
|  |  |  | 0 |
|  |  |  | 0 |
|  |  |  | 0 |
|  |  |  | 0 |
|  |  |  | 0 |
|  |  |  | 0 |
|  |  |  | 0 |
|  |  |  | 0 |
|  |  |  | 0 |
|  |  |  | 0 |
|  |  |  | 0 |
|  |  |  | 0 |
|  |  |  | 0 |
|  |  |  | 0 |
|  |  |  | 0 |
|  |  |  | 0 |
|  |  |  | 0 |
|  |  |  | 0.19889503 |
|  |  |  | 0 |
|  |  |  | 0 |
|  |  |  | 0 |
|  |  |  | 0 |
|  |  |  | 0.60674157 |
|  |  |  | 0.20571429 |
|  |  |  | 0 |
|  |  |  | 0 |
|  |  |  | 0 |
|  |  |  | 0 |
|  |  |  | 0 |
|  |  |  | 0 |
|  |  |  | 0 |
|  |  |  | 0 |
|  |  |  | 0 |
|  |  |  | 0 |
|  |  |  | 0 |
|  |  |  | 0 |
|  |  |  | 0 |
|  |  |  | 0 |
|  |  |  | 0 |
|  |  |  | 0 |
|  |  |  | 0 |
|  |  |  | 0 |
|  |  |  | 0 |
|  |  |  | 0 |
|  |  |  | 0 |
|  |  |  | 0 |
|  |  |  | 0 |
|  |  |  | 0 |
|  |  |  | 0 |
|  |  |  | 0 |
|  |  |  | 0 |
|  |  |  | 0 |
|  |  |  | 0 |
|  |  |  | 0 |
|  |  |  | 0 |
|  |  |  | 0 |
|  |  |  | 0 |
|  |  |  | 0 |
|  |  |  | 0.32432432 |
|  |  |  | 0 |
|  |  |  | 0 |
|  |  |  | 0 |
|  |  |  | 0 |
|  |  |  | 0 |
|  |  |  | 0 |
|  |  |  | 0.34285714 |
|  |  |  | 0 |
|  |  |  | 0 |
|  |  |  | 0 |
|  |  |  | 0 |
|  |  |  | 0 |
|  |  |  | 0 |
|  |  |  | 0 |
|  |  |  | 0 |
|  |  |  | 0 |
|  |  |  | 0 |
|  |  |  | 0 |
|  |  |  | 0 |
|  |  |  | 0 |
|  |  |  | 0 |
|  |  |  | 0 |
|  |  |  | 0 |
|  |  |  | 0 |
|  |  |  | 0 |
|  |  |  | 0 |
|  |  |  | 0 |
|  |  |  | 0 |
|  |  |  | 0 |
|  |  |  | 0 |
|  |  |  | 0 |
