# Supplementary material for: Telomere damage induces internal loops that generate telomeric circles
Source: Nat Commun. 2020 Oct 20;11:5297. doi: 10.1038/s41467-020-19139-4 (PMC7576219; doi:10.1038/s41467-020-19139-4)
Supplement: Supplementary file 6 — Source Data [file 41467_2020_19139_MOESM6_ESM.zip › Source data 2nd rev/Source data Supplementary Figure 6.pdf]

Figure S6A

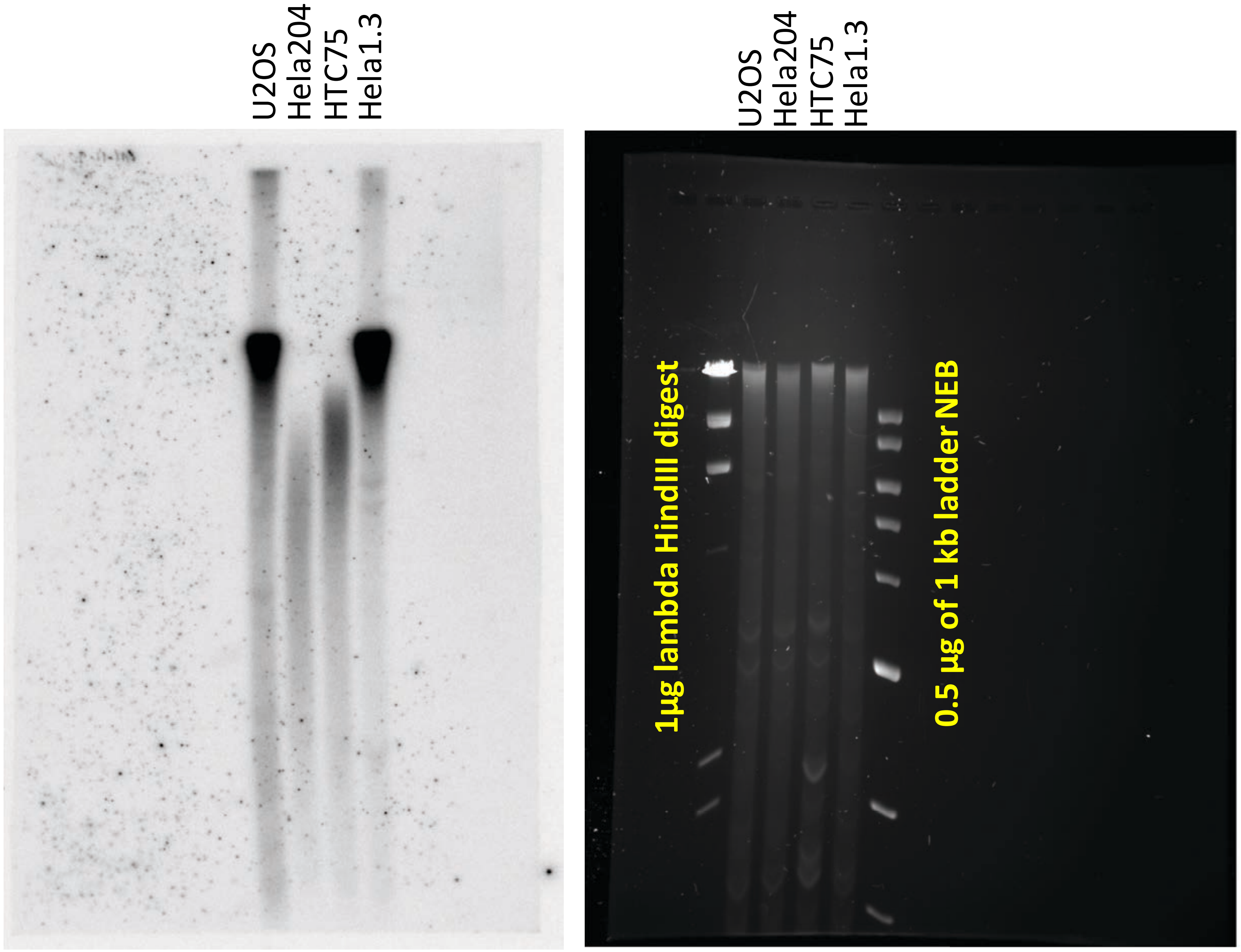
U2OS
Hela204
HTC75
Hela1.3
U2OS
Hela204
HTC75
Hela1.3
1µg lambda HindIII digest
0.5 µg of 1 kb ladder NEB

Figure S6B

DNaseI treatment in Hela1.3 nuclei

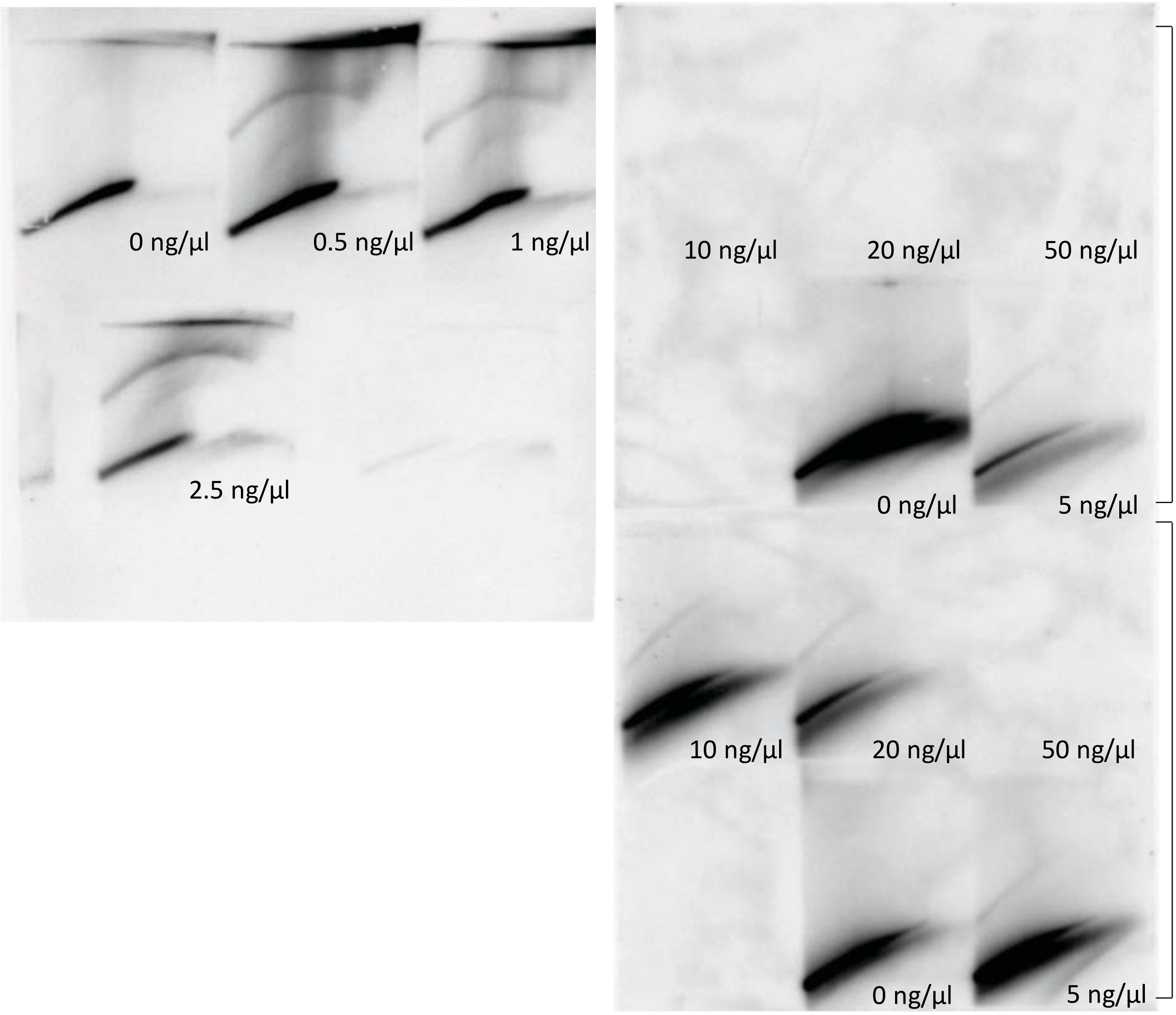

DNaseI treatment in HeLa204 nuclei

DNaseI treatment in HTC75 nuclei

Figure S6C

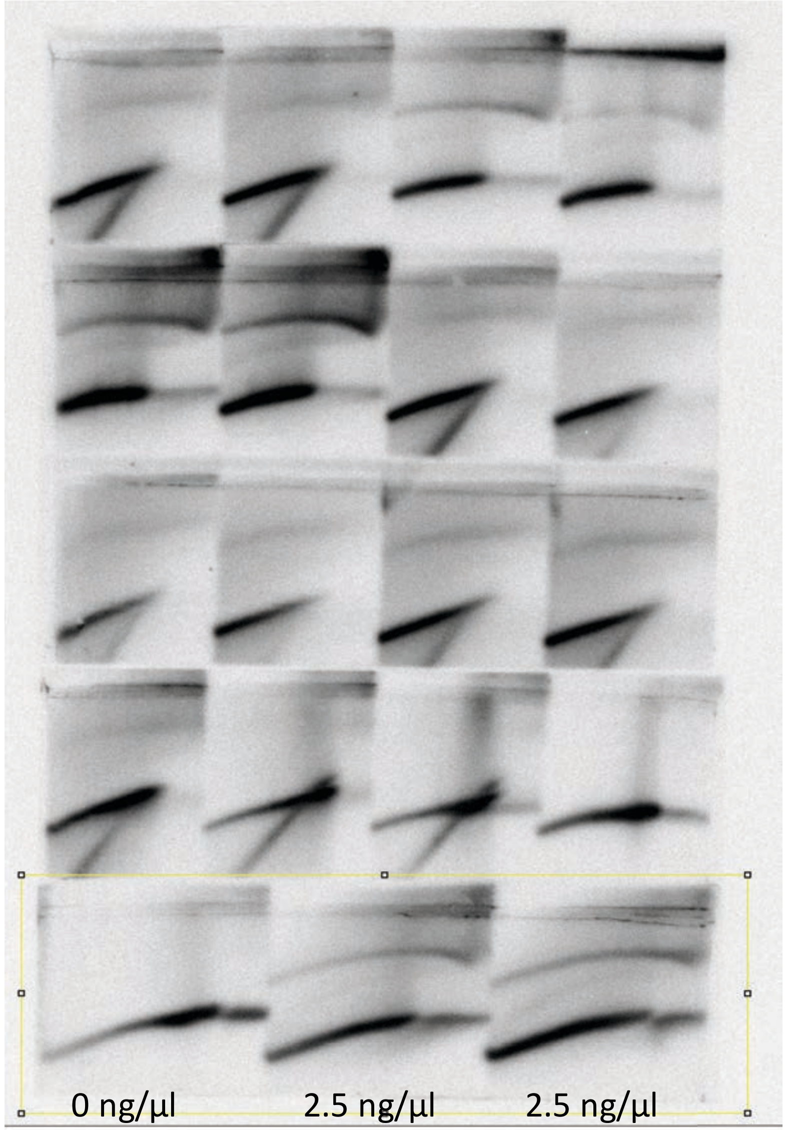

telomere probe

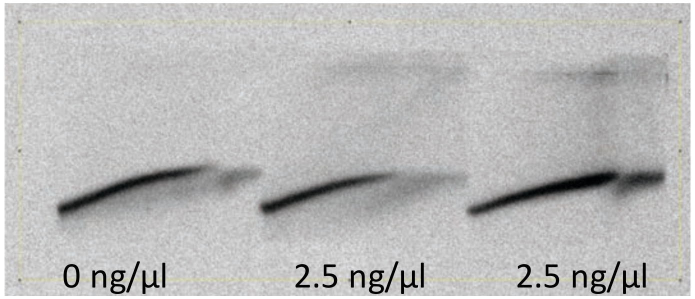

L1 repeats probe

DNaseI treatment in MEFs nuclei, telomeric probe

Figure S6D

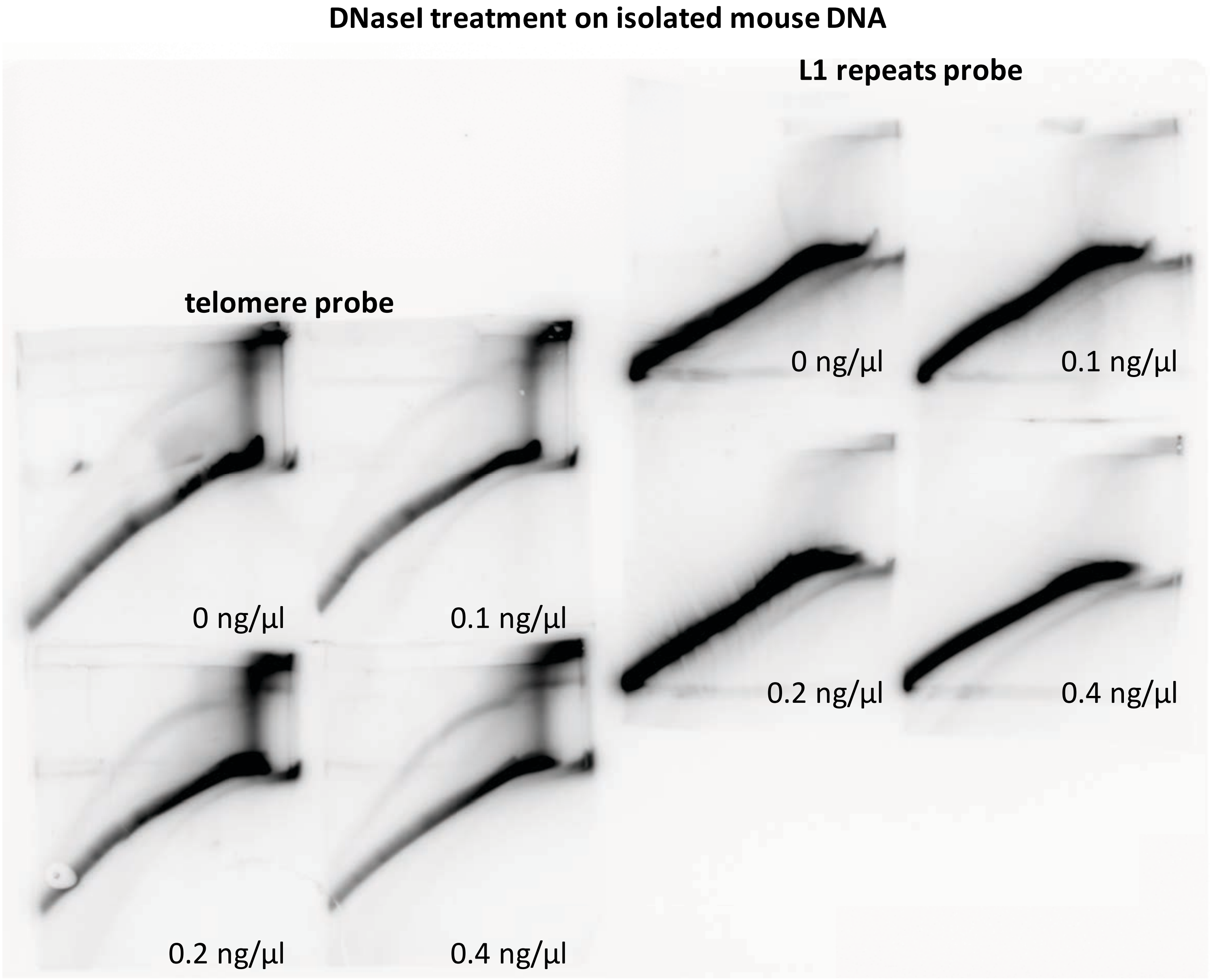
DNaseI treatment on isolated mouse DNA
L1 repeats probe
telomere probe
0 ng/µl
0.1 ng/µl
0 ng/µl
0.1 ng/µl
0.2 ng/µl
0.4 ng/µl
0.2 ng/µl
0.4 ng/µl
