# Supplementary figures and images for: Telomere damage induces internal loops that generate telomeric circles
Source: Nat Commun. 2020 Oct 20;11:5297. doi: 10.1038/s41467-020-19139-4 (PMC7576219; doi:10.1038/s41467-020-19139-4)

Figure 1A

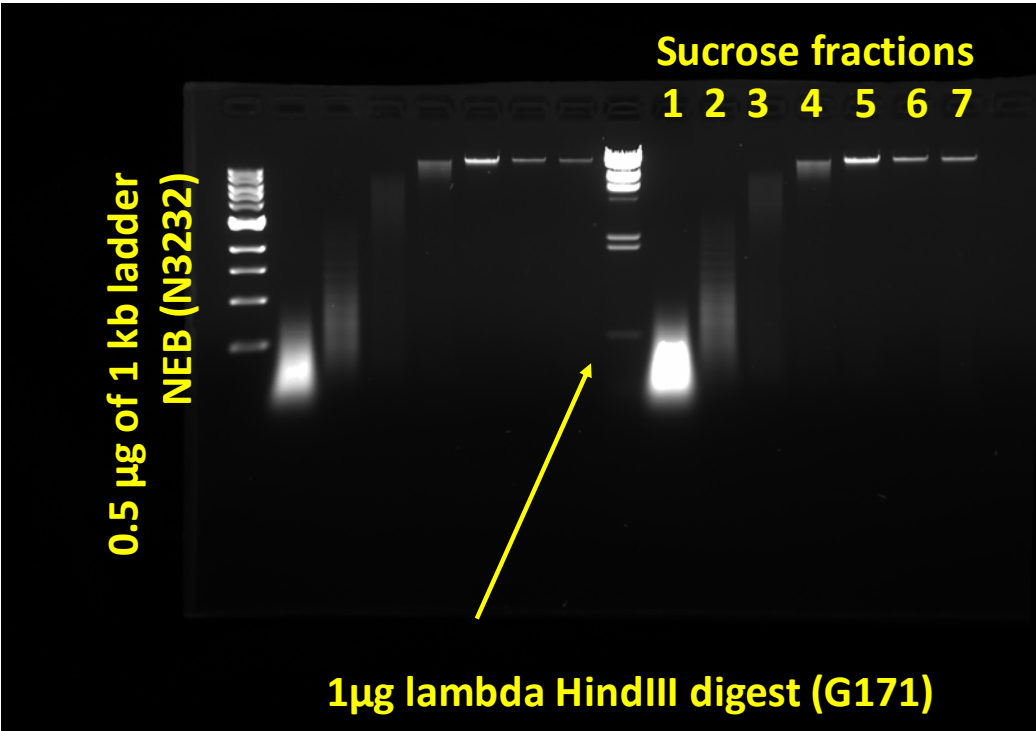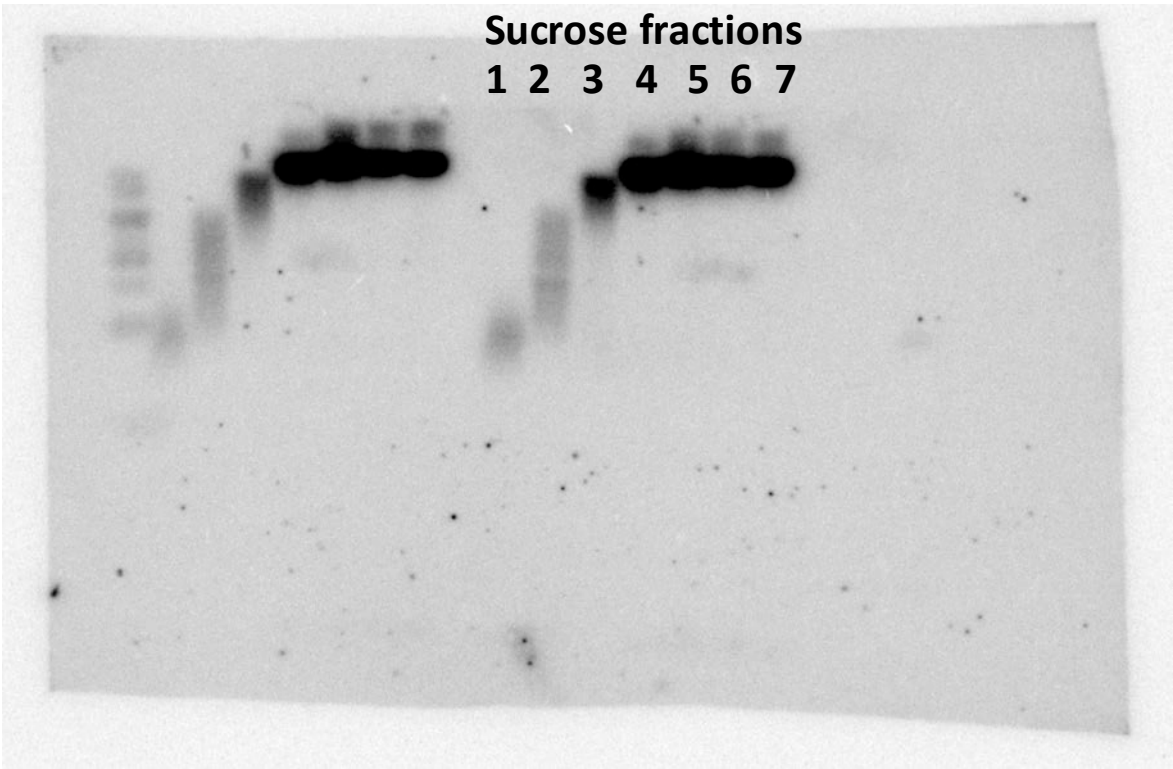

Figure 1B

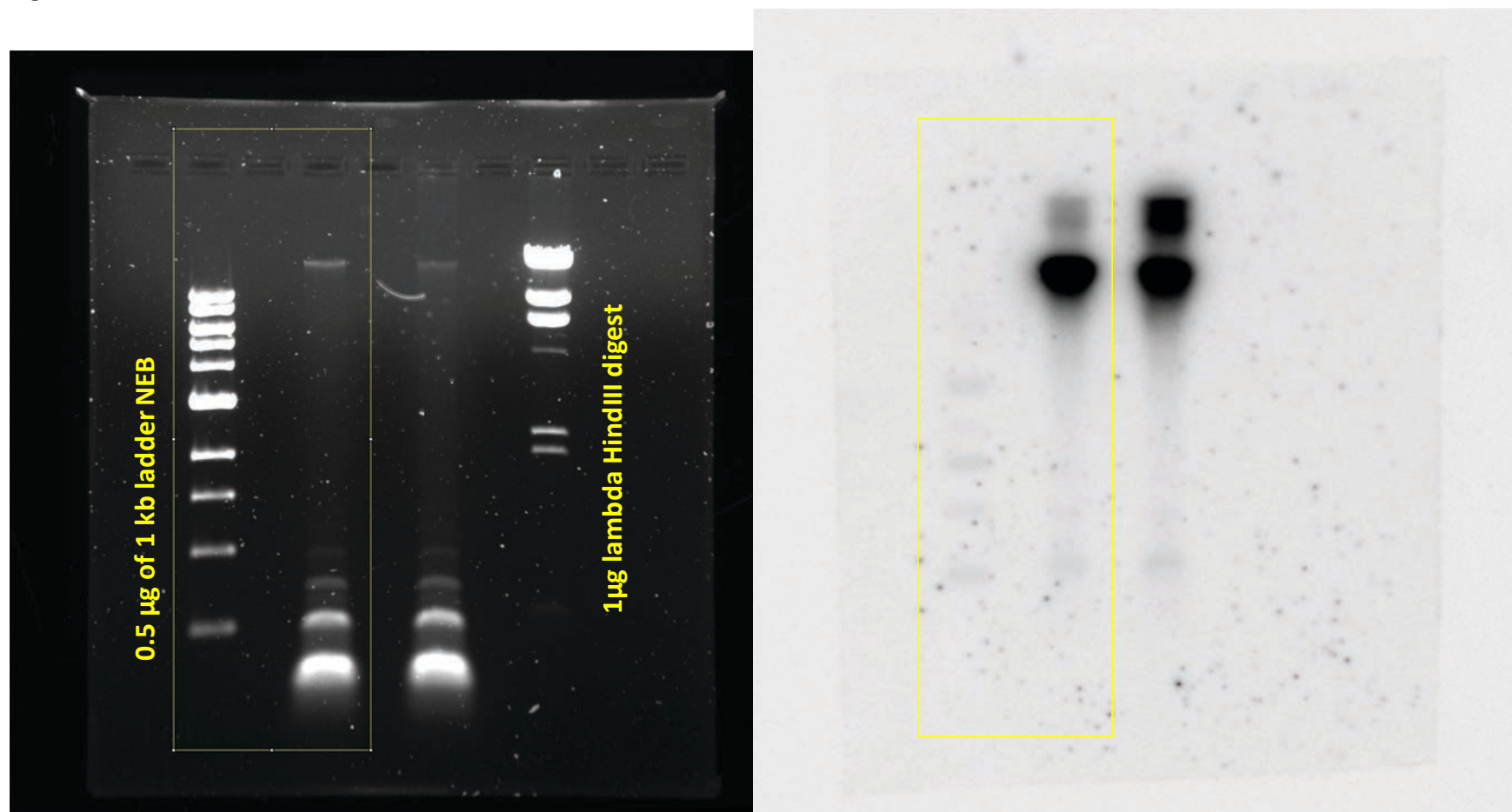

**Figure 1C**

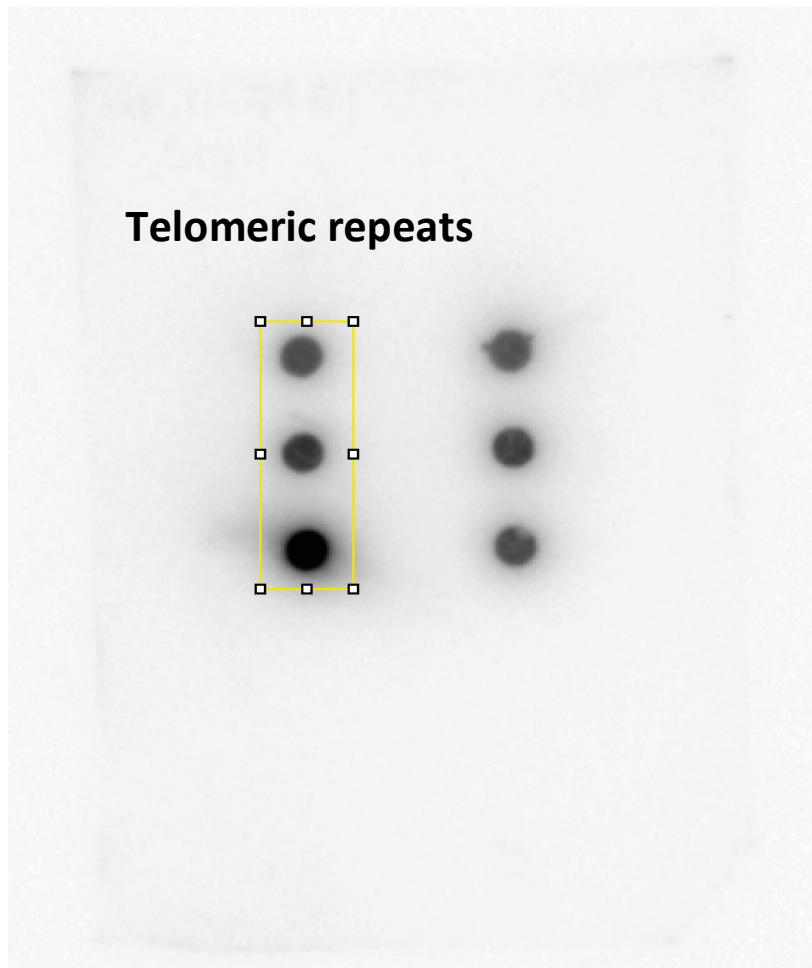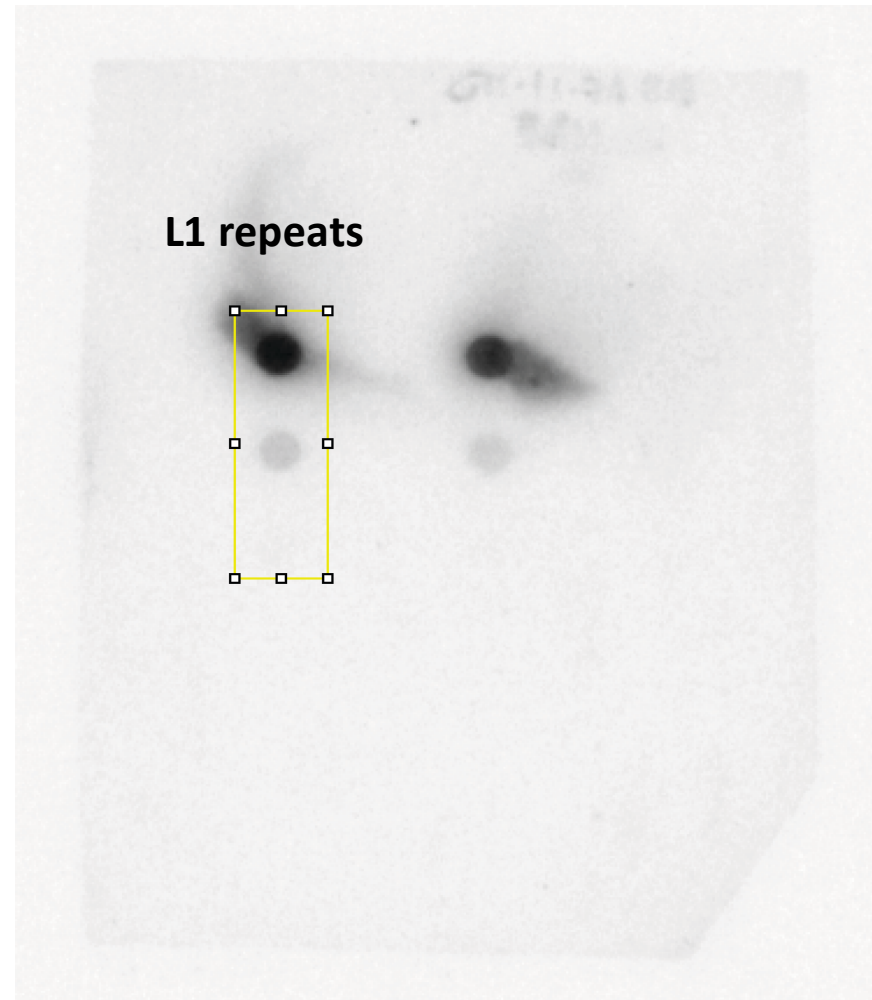

Supplement: Supplementary file 6 — Source Data [file 41467_2020_19139_MOESM6_ESM.zip › Source data 2nd rev/Source data Figure 1.pdf]

**Figure 3**
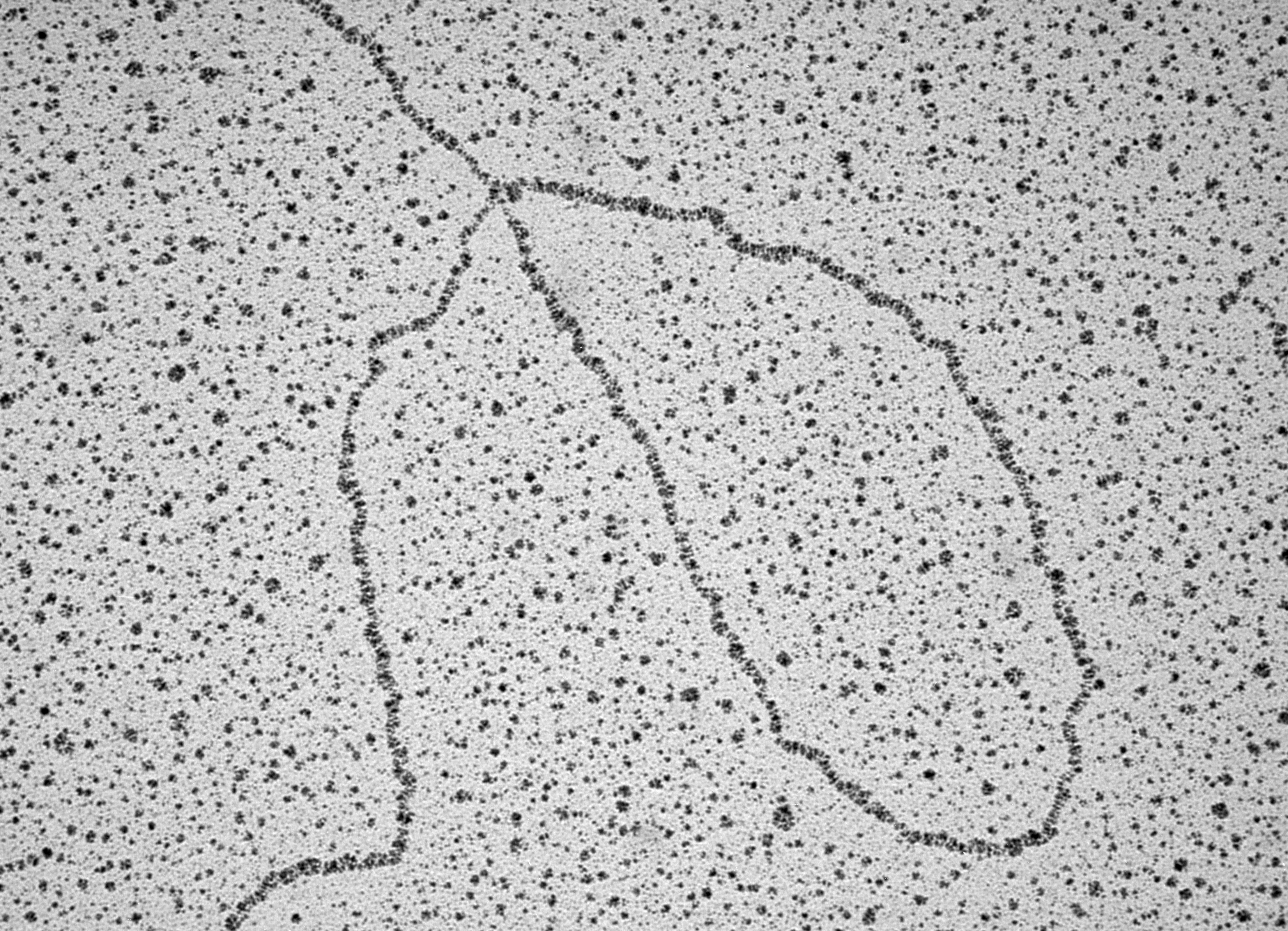

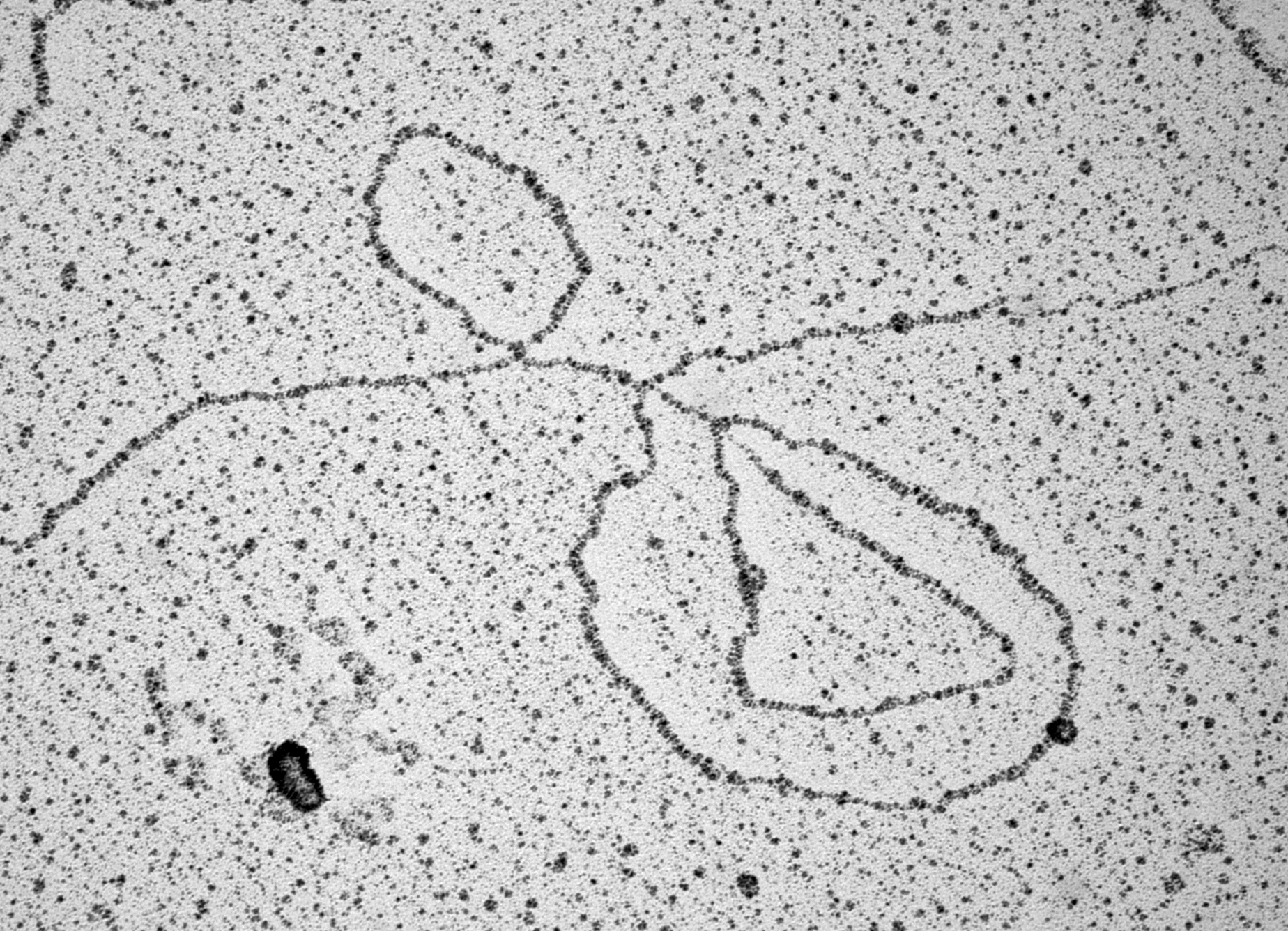

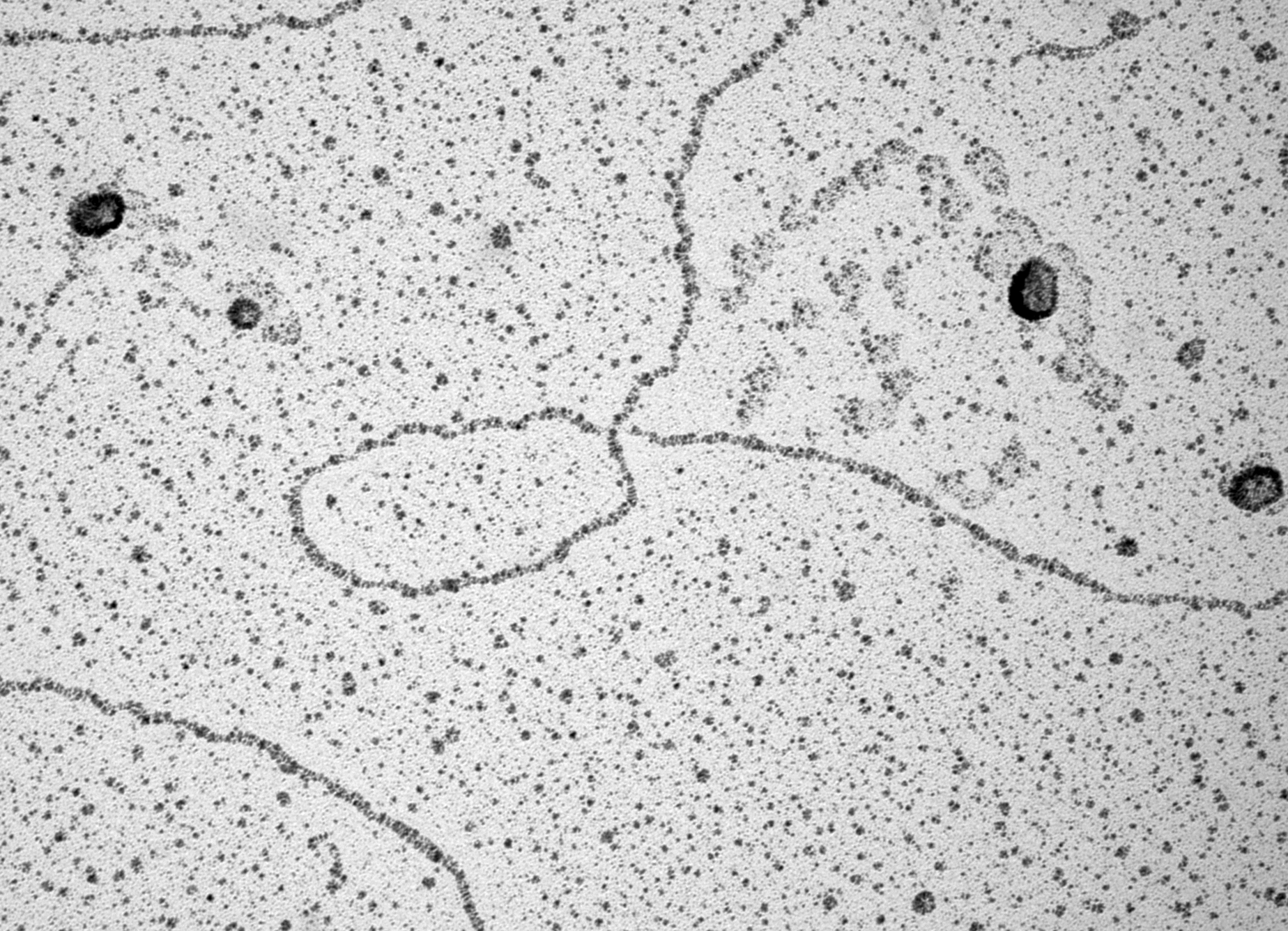

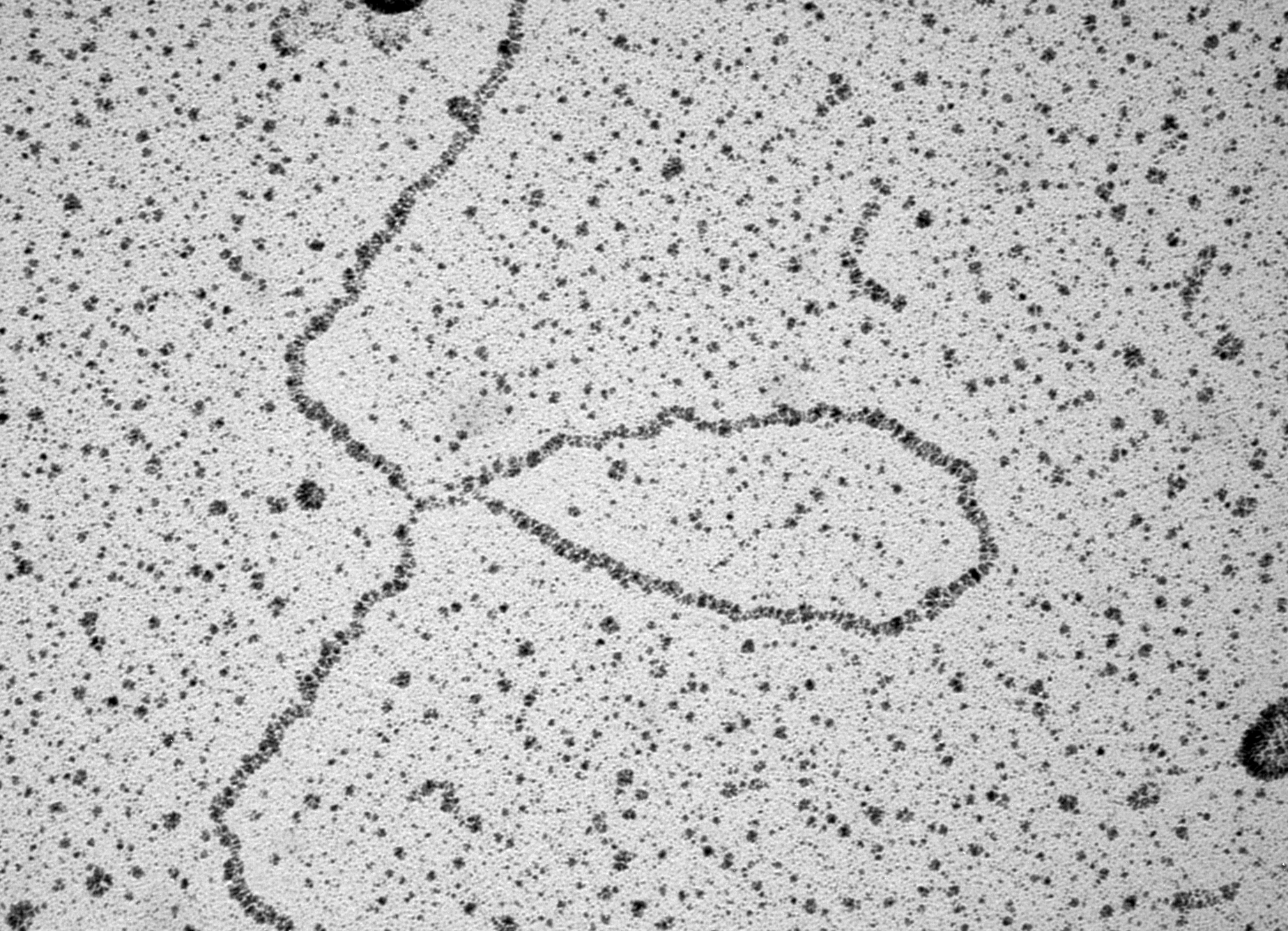

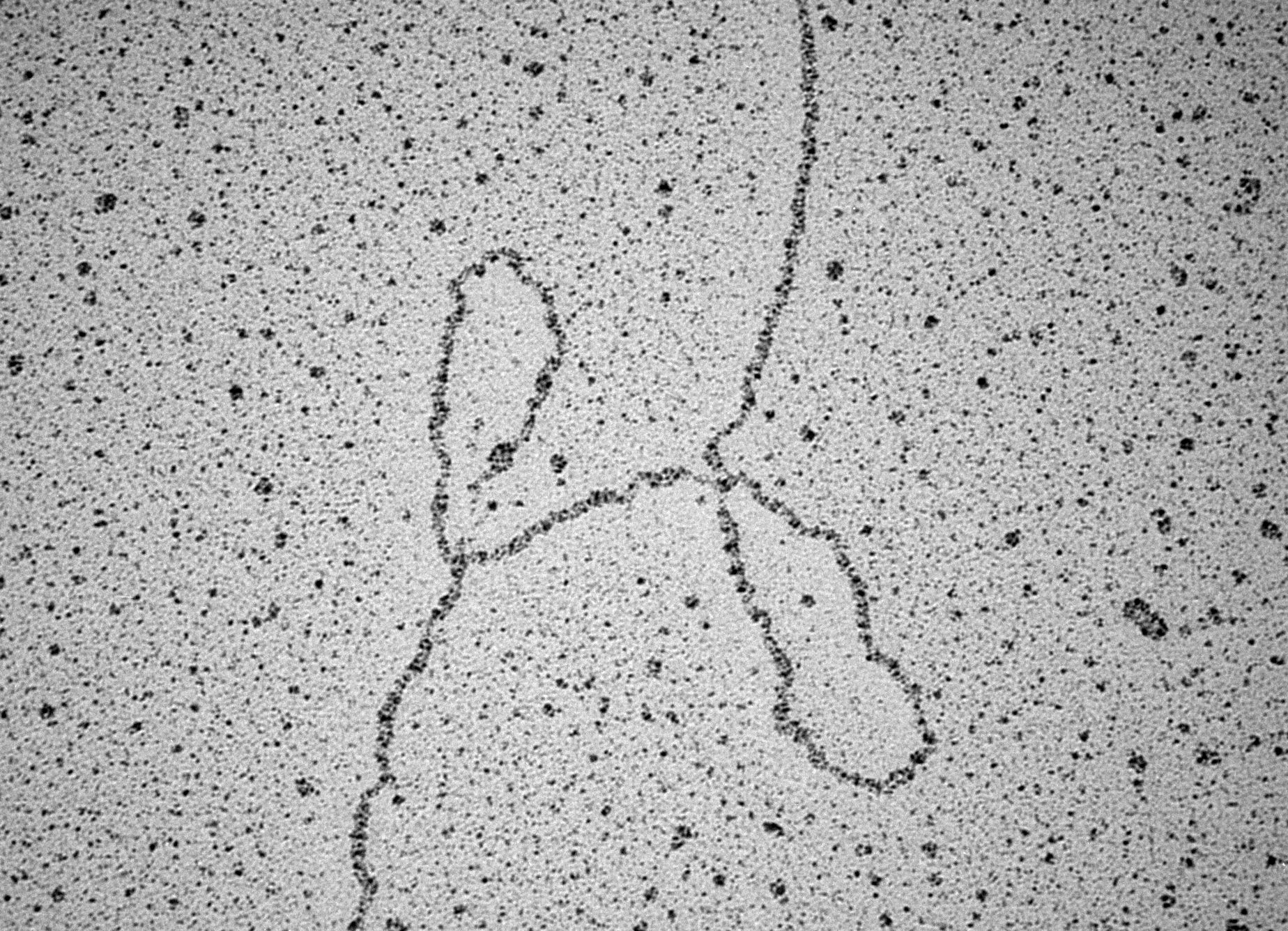

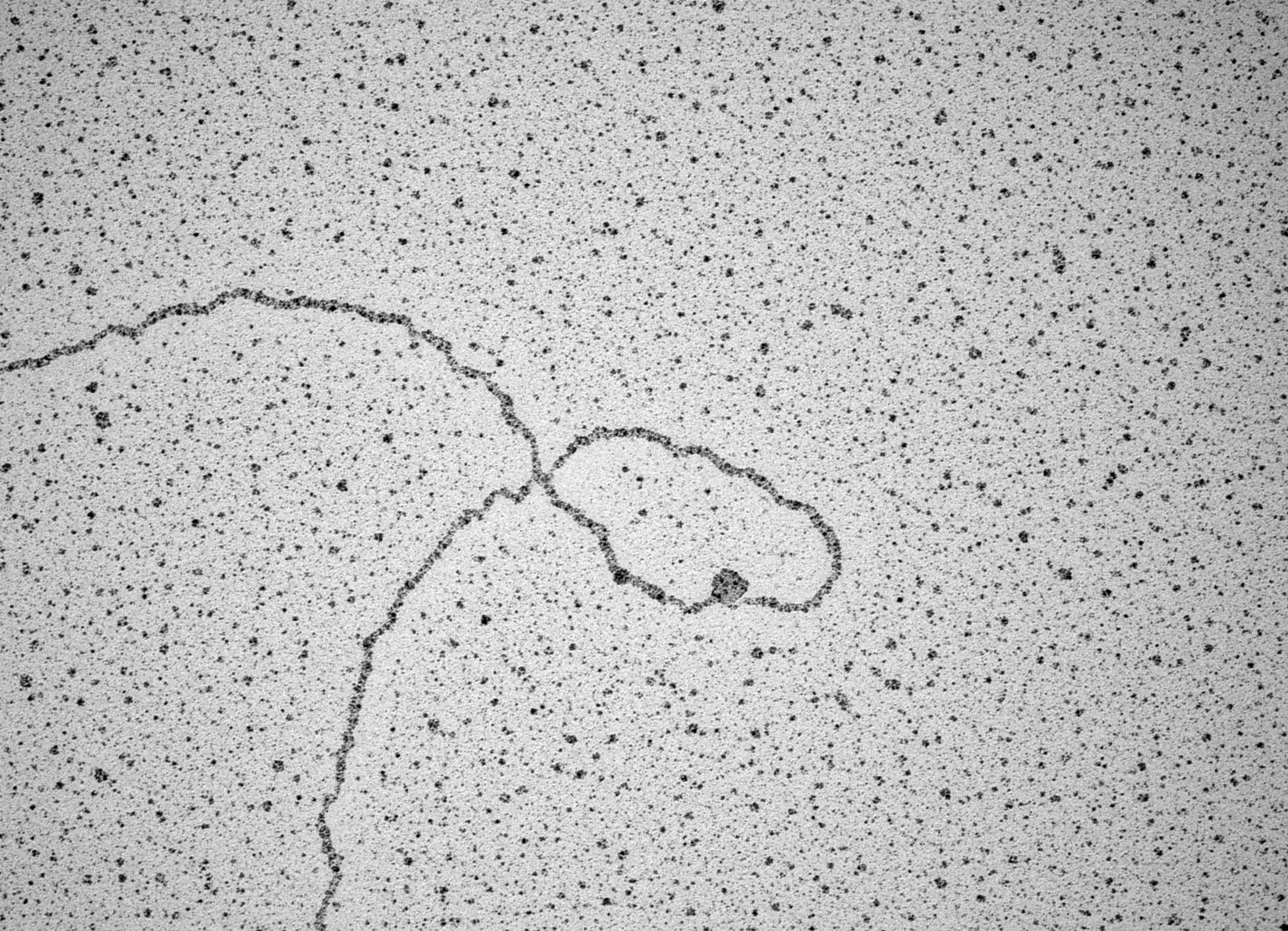

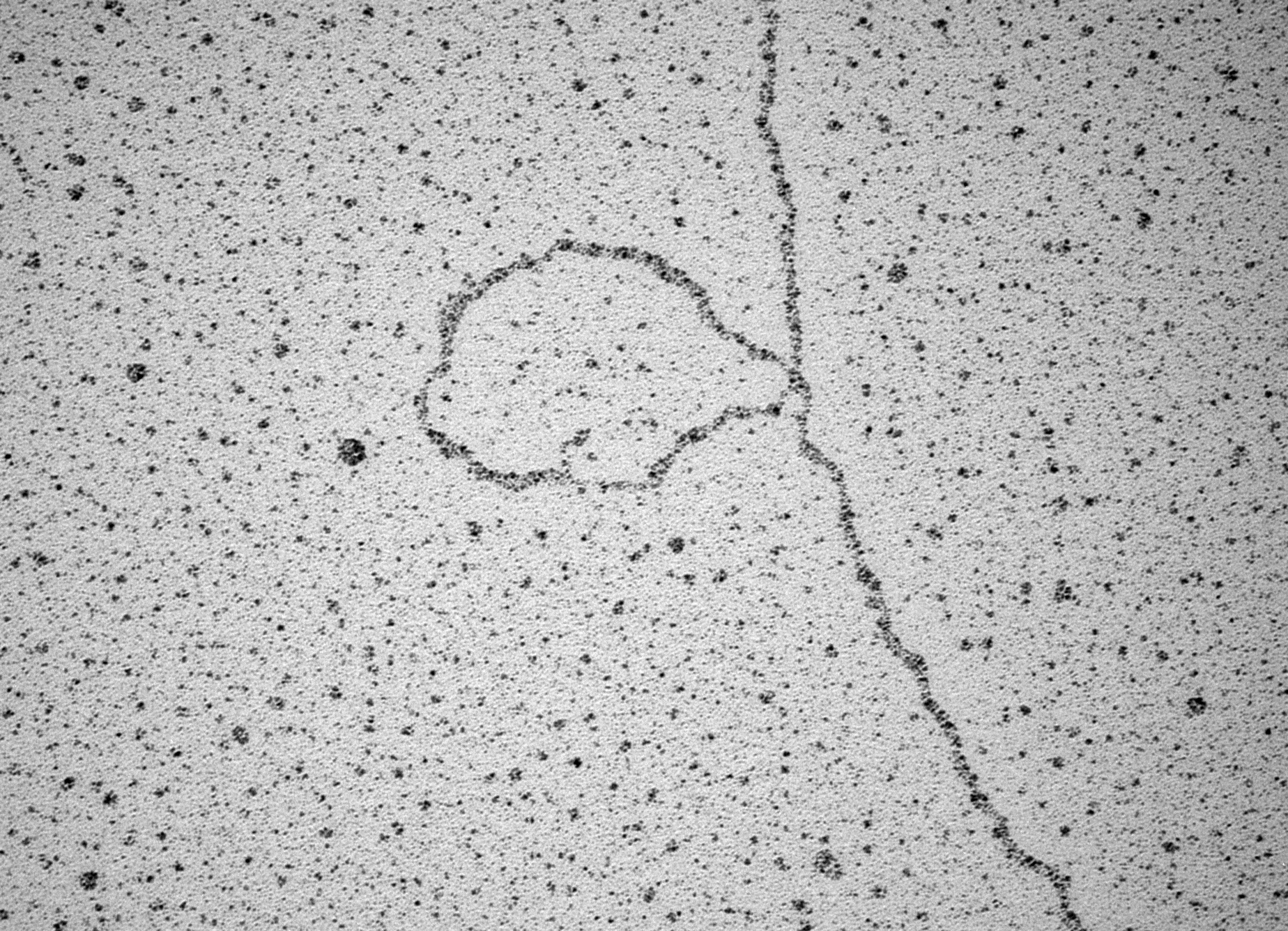

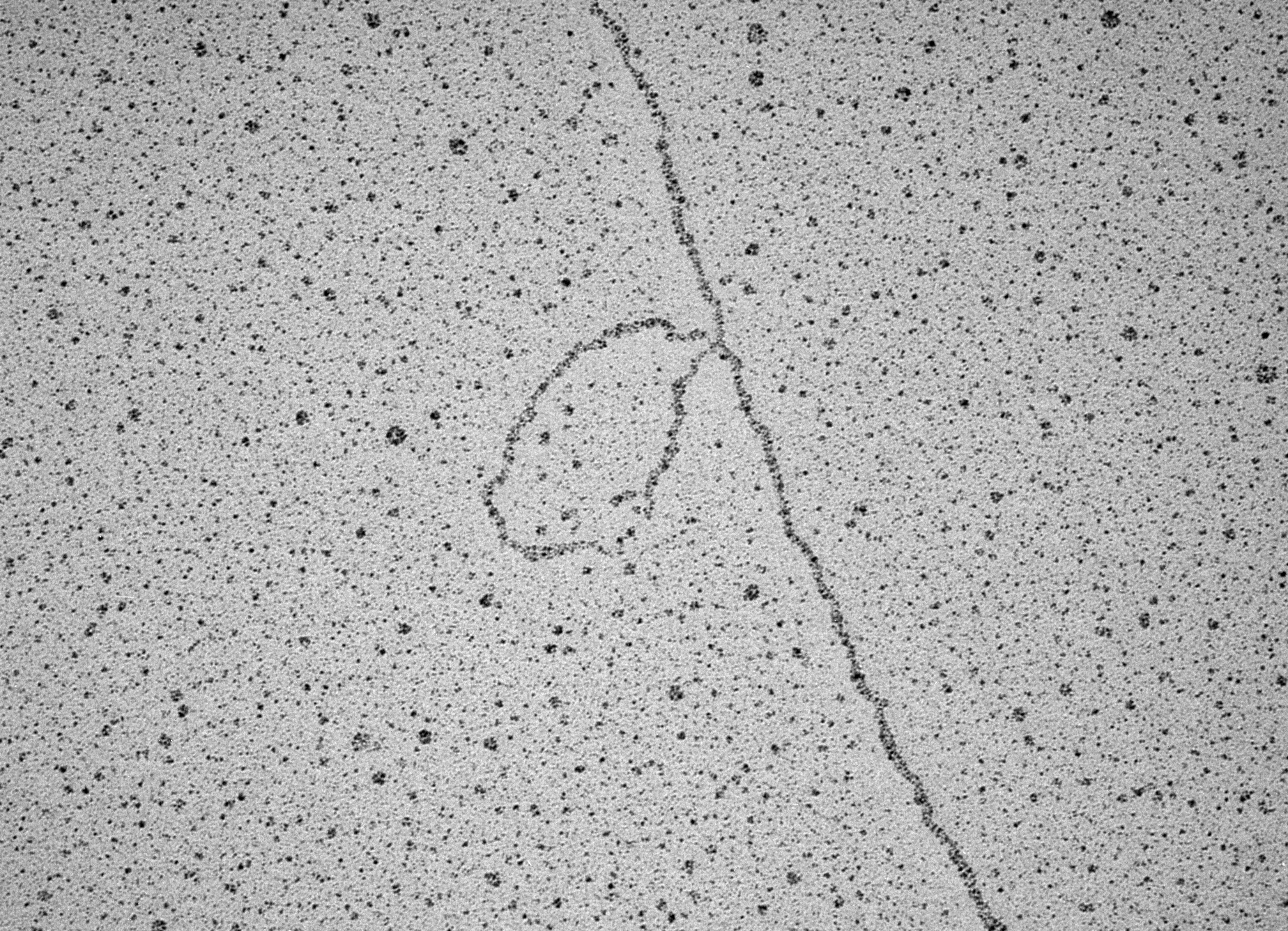

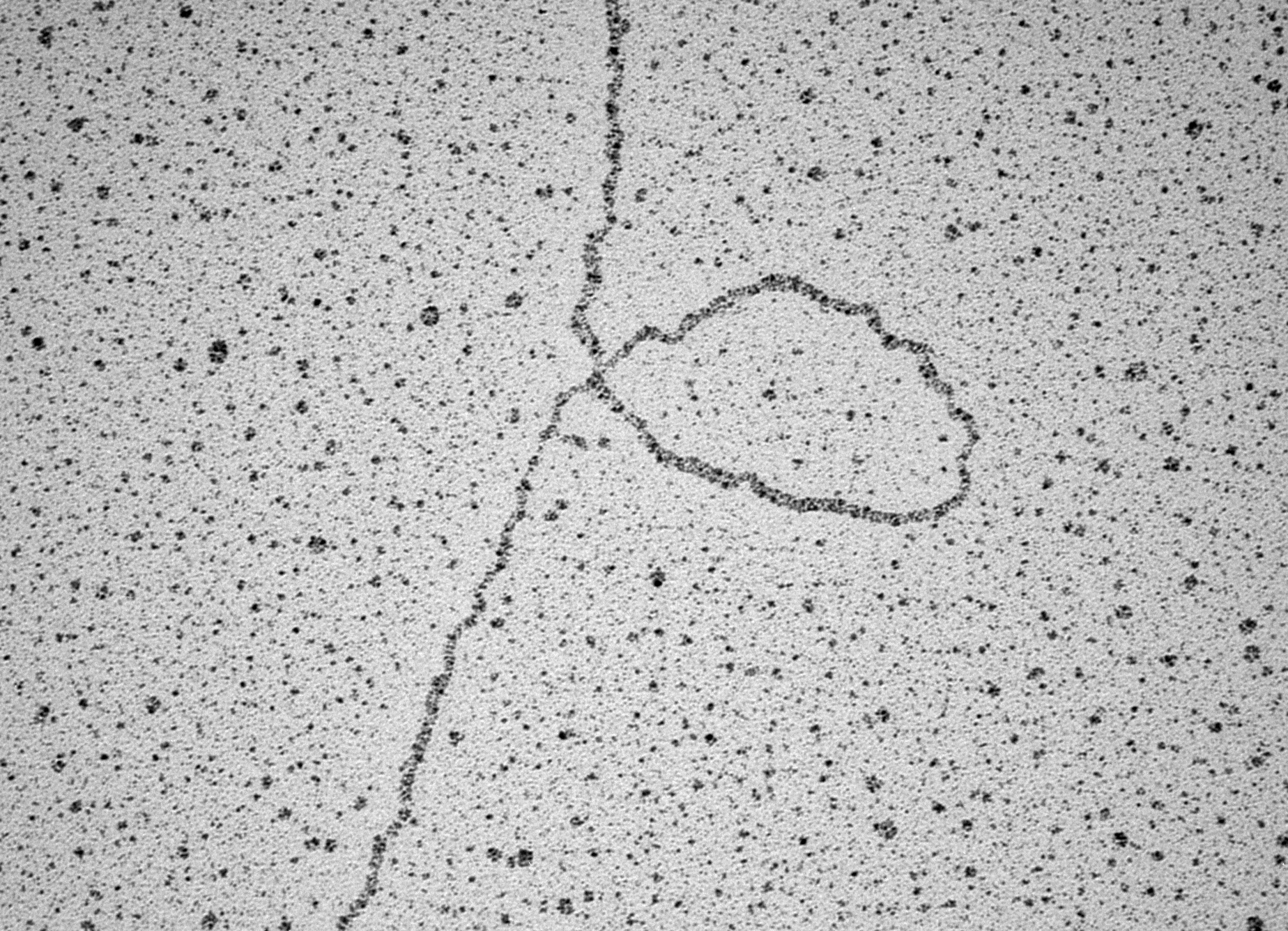

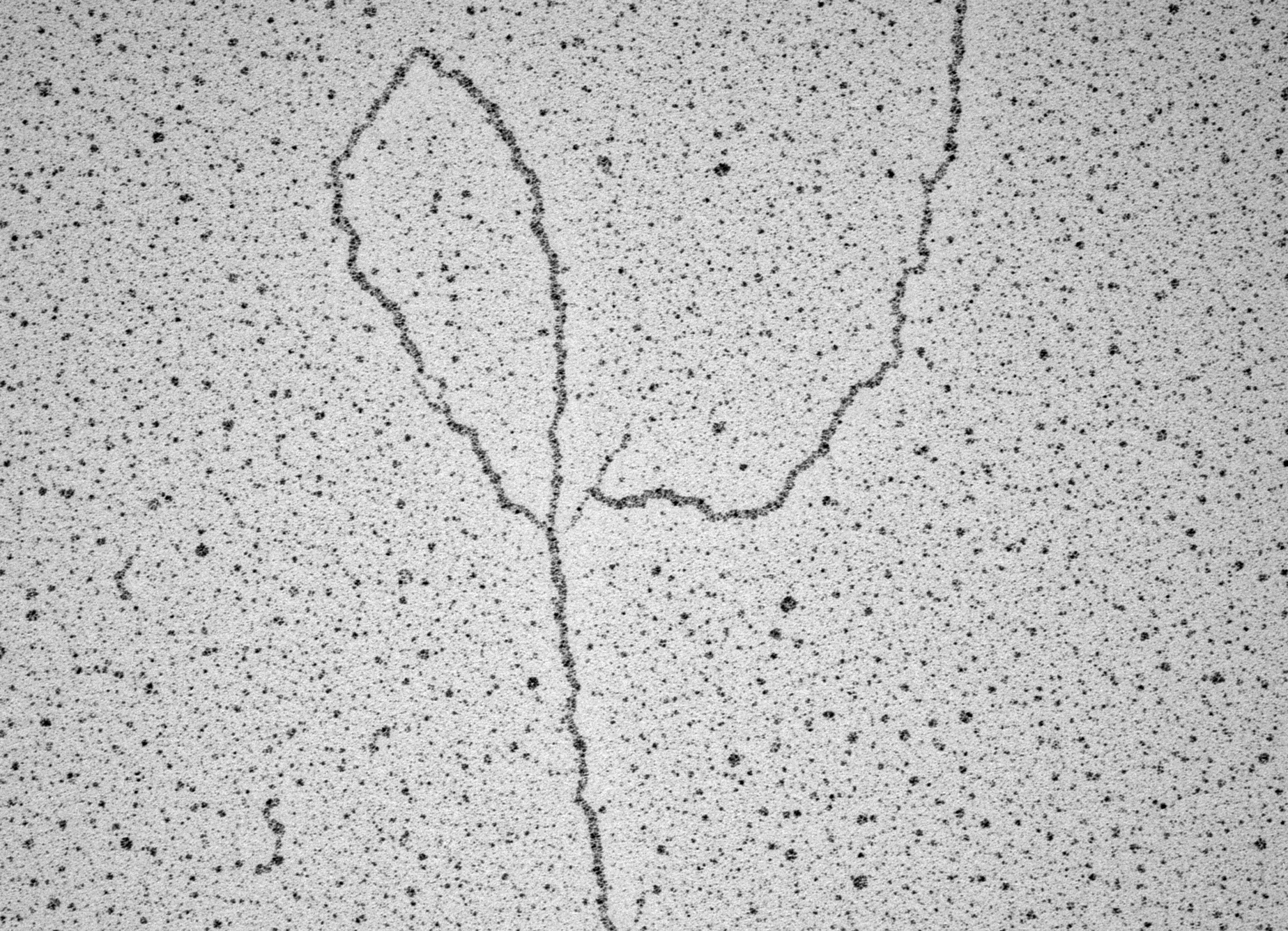

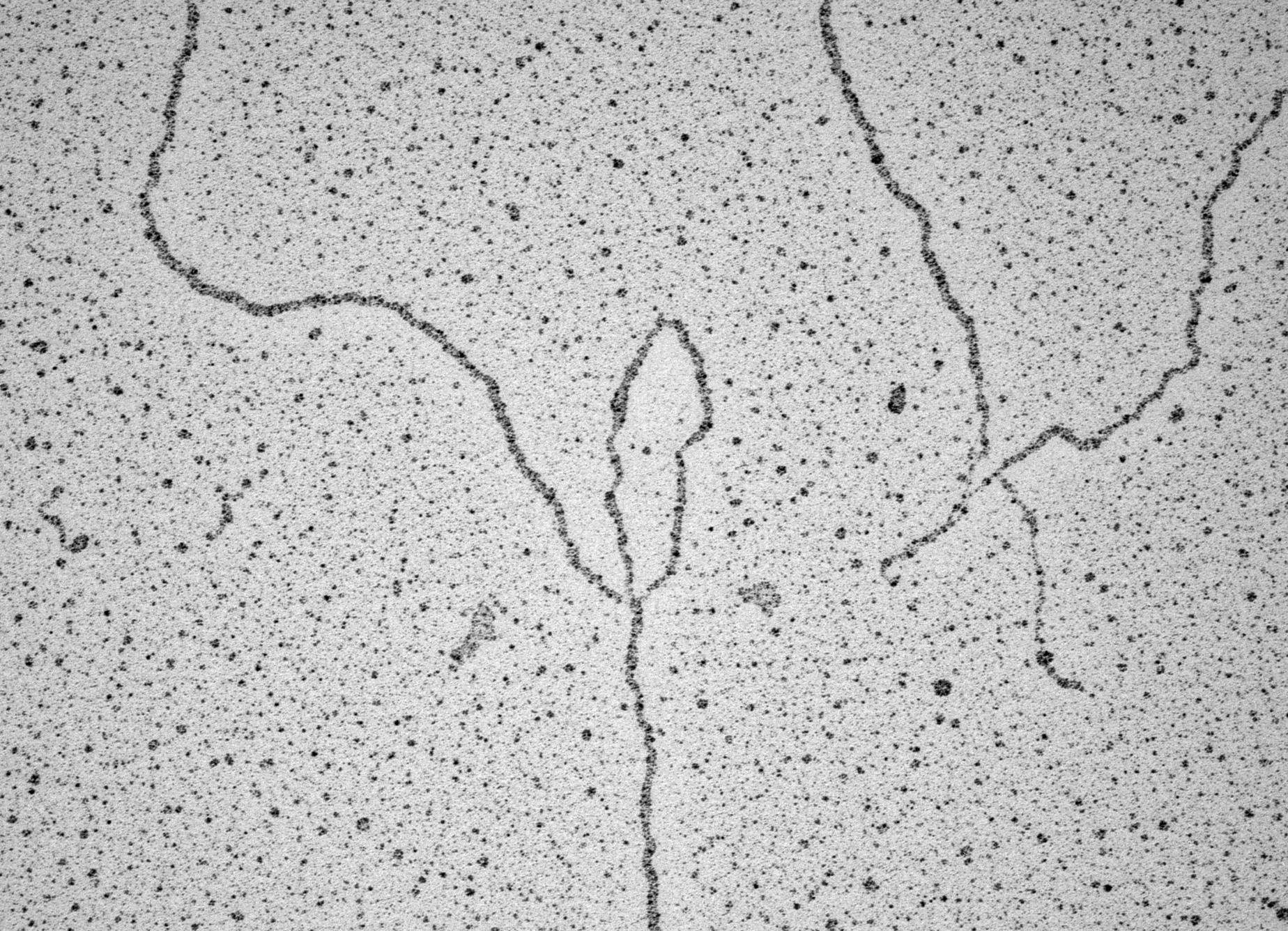

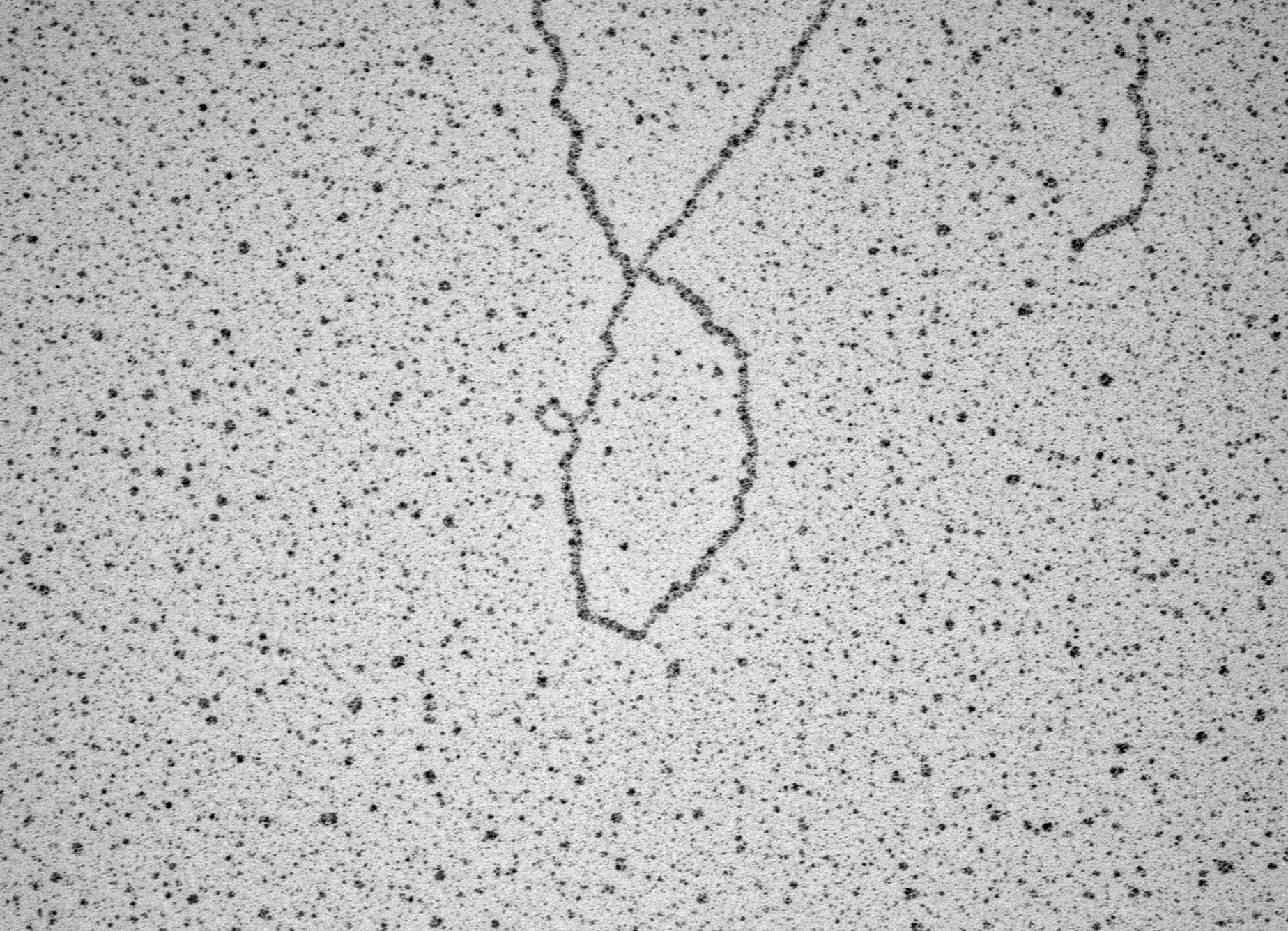

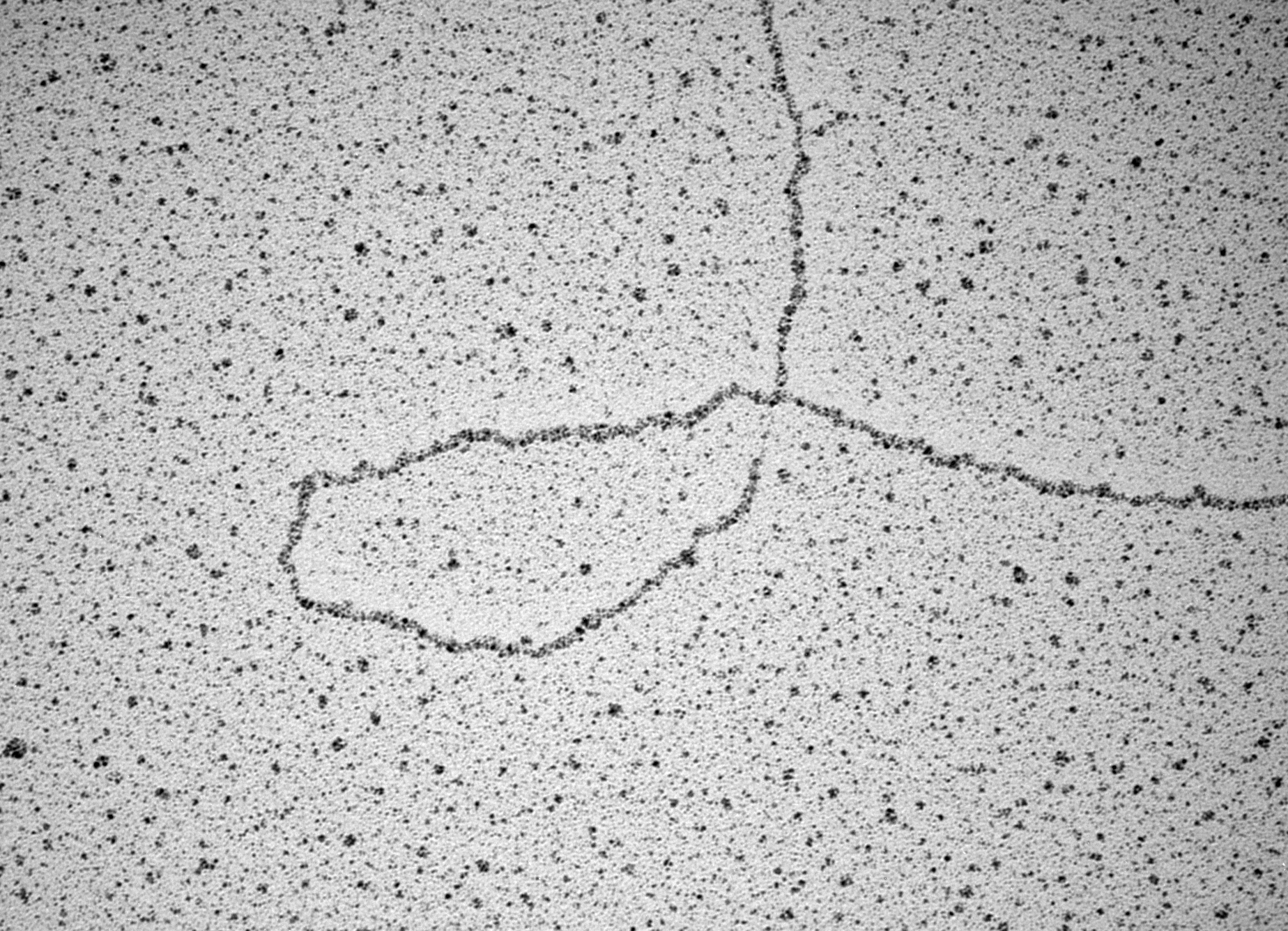

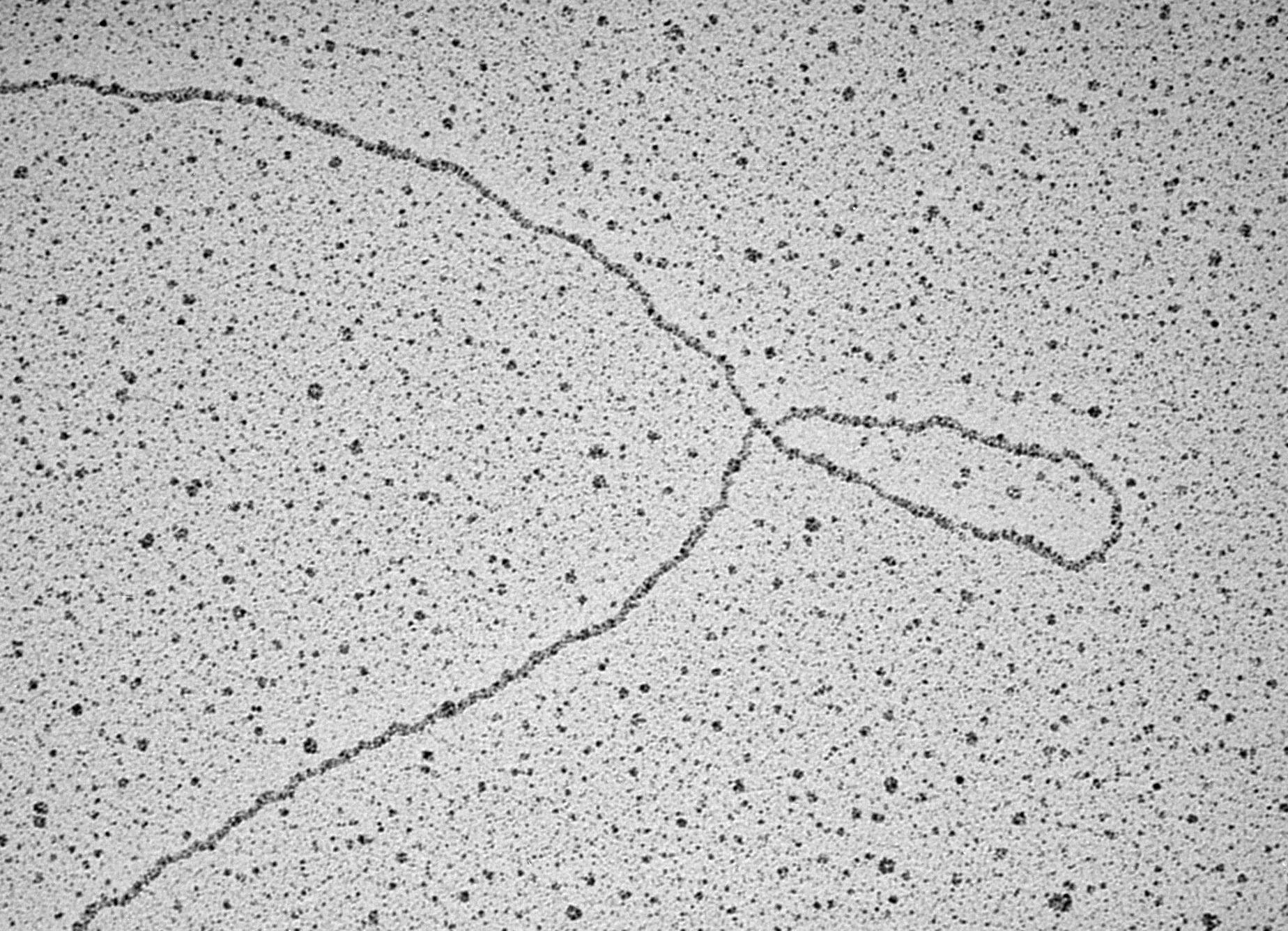

Supplement: Supplementary file 6 — Source Data [file 41467_2020_19139_MOESM6_ESM.zip › Source data 2nd rev/Source data Figure 3.docx]

Figure 4A

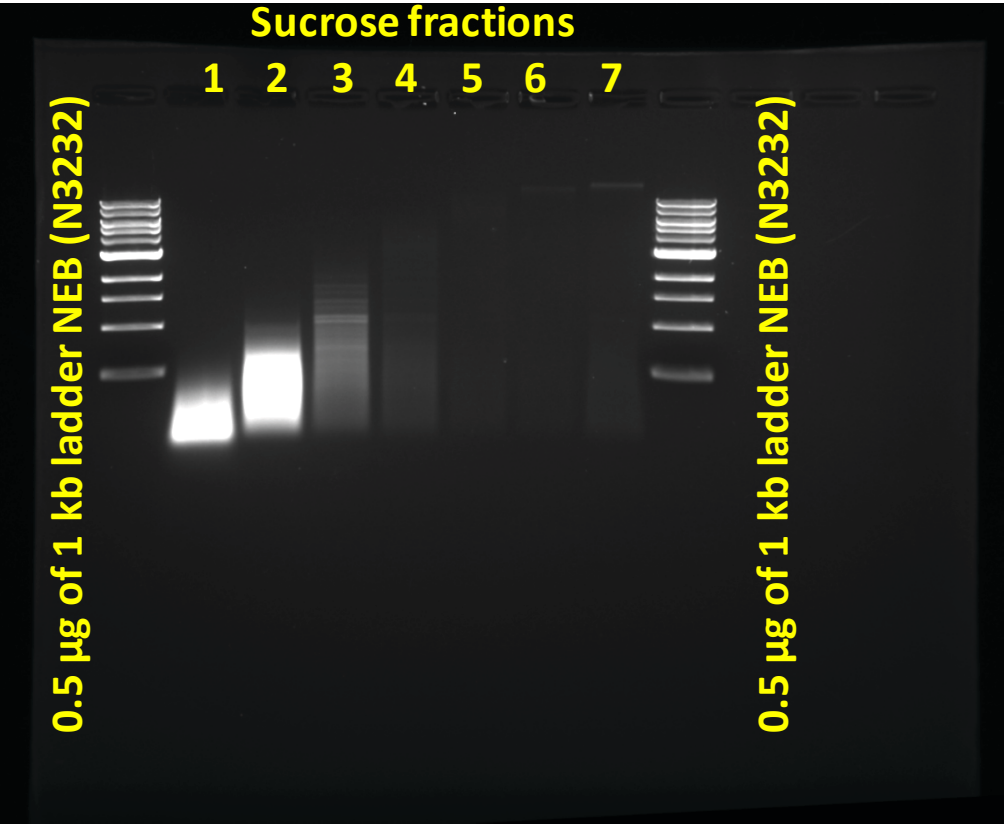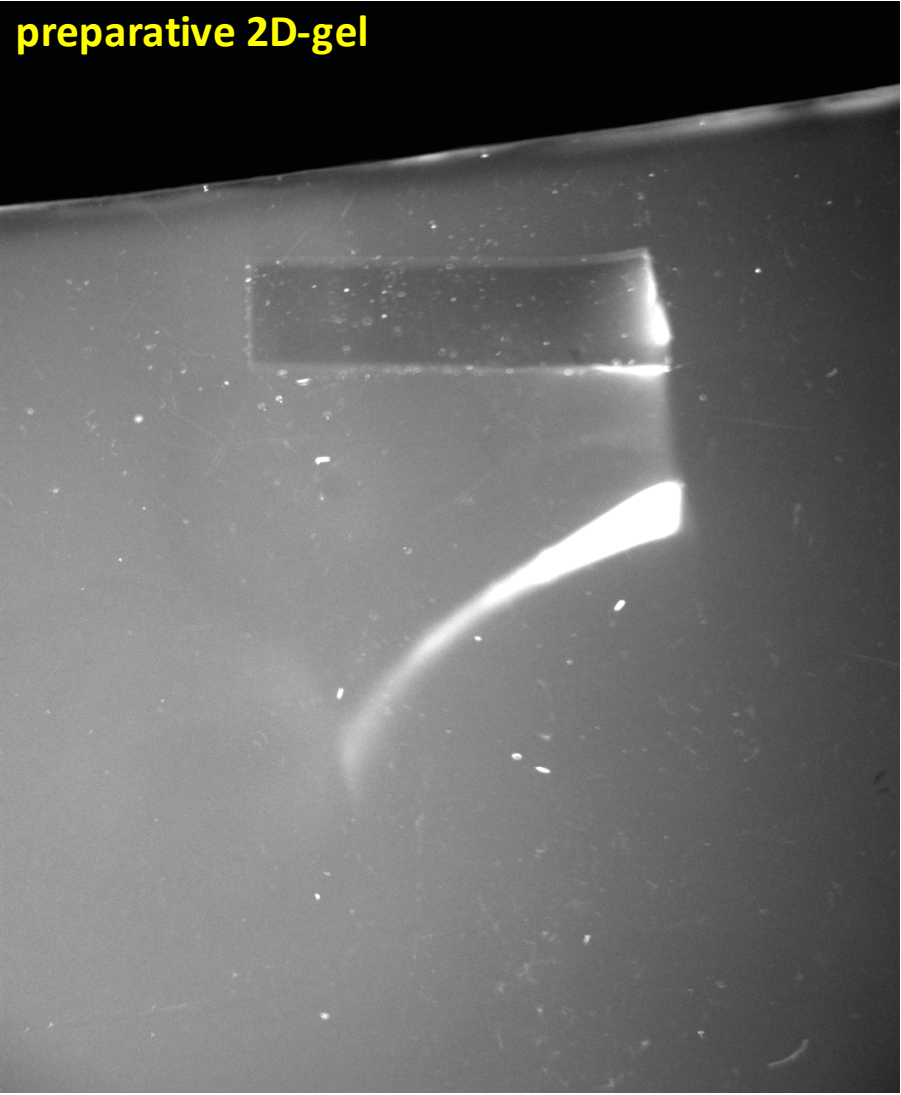

Figure 4c

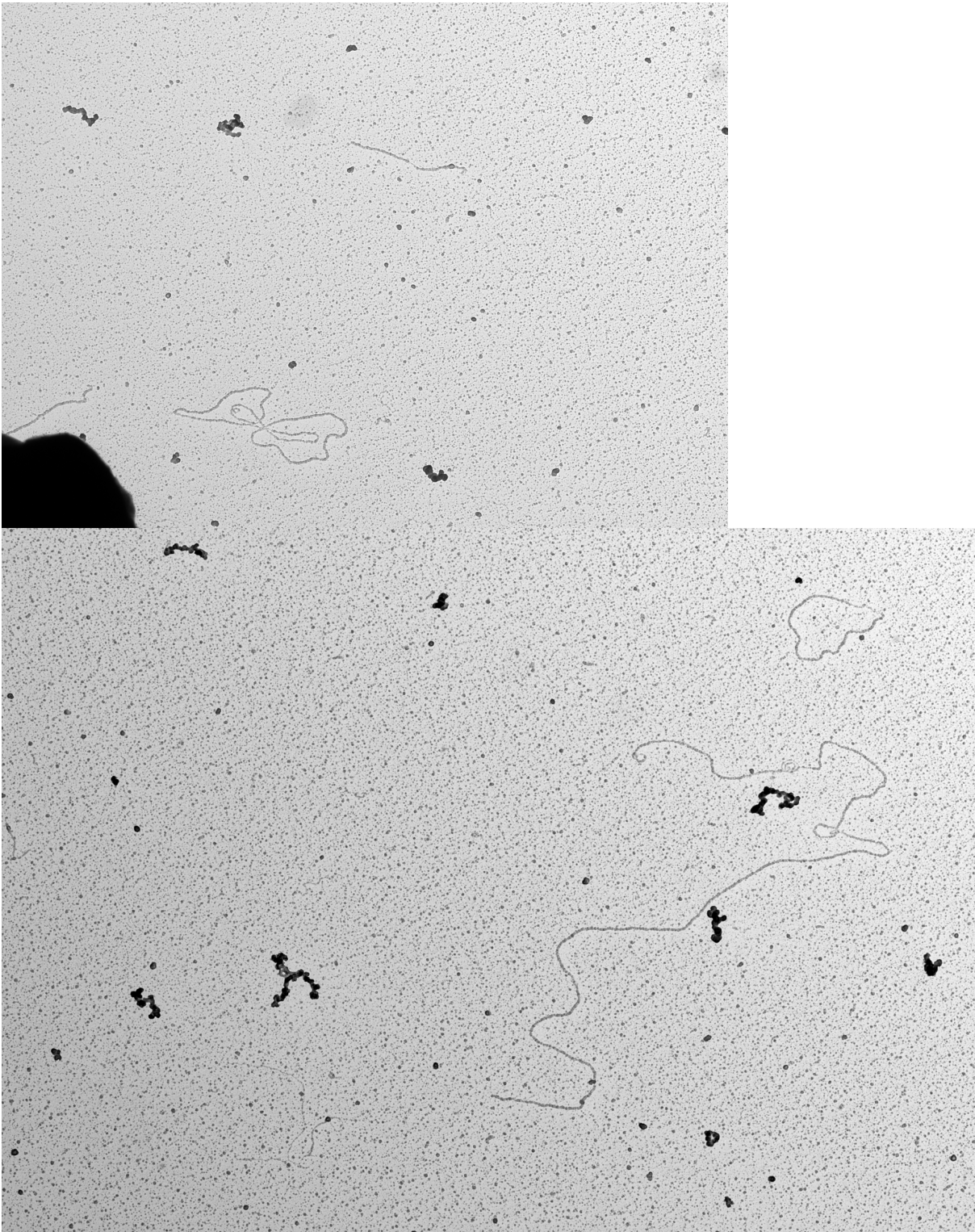

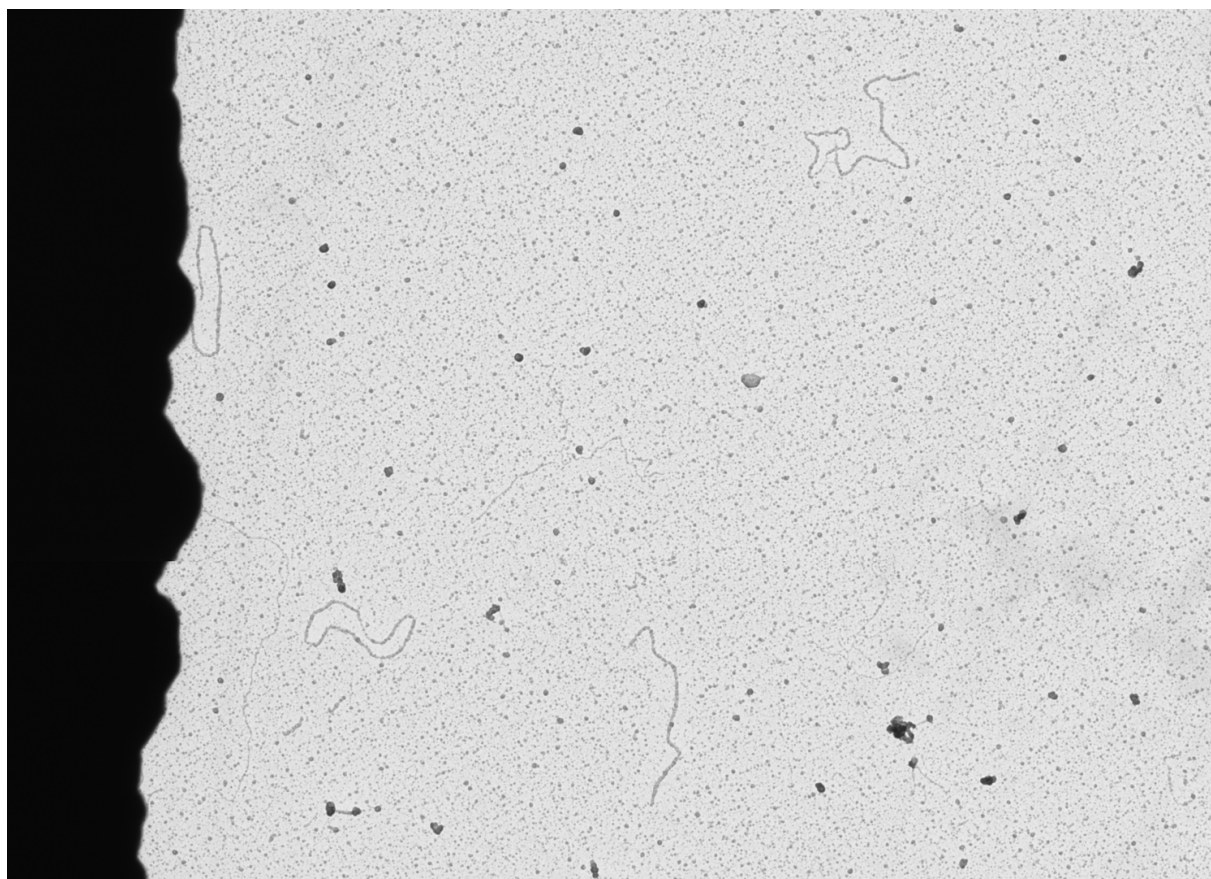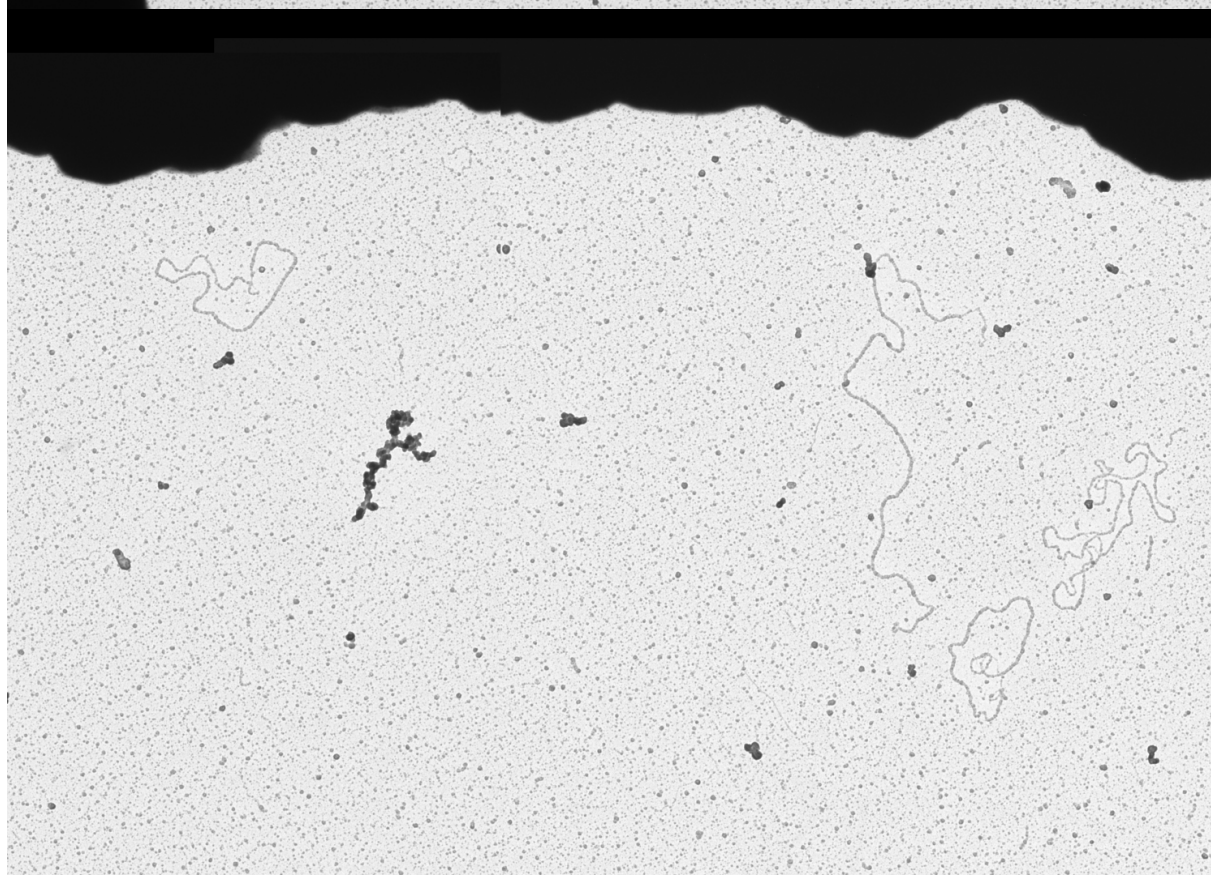

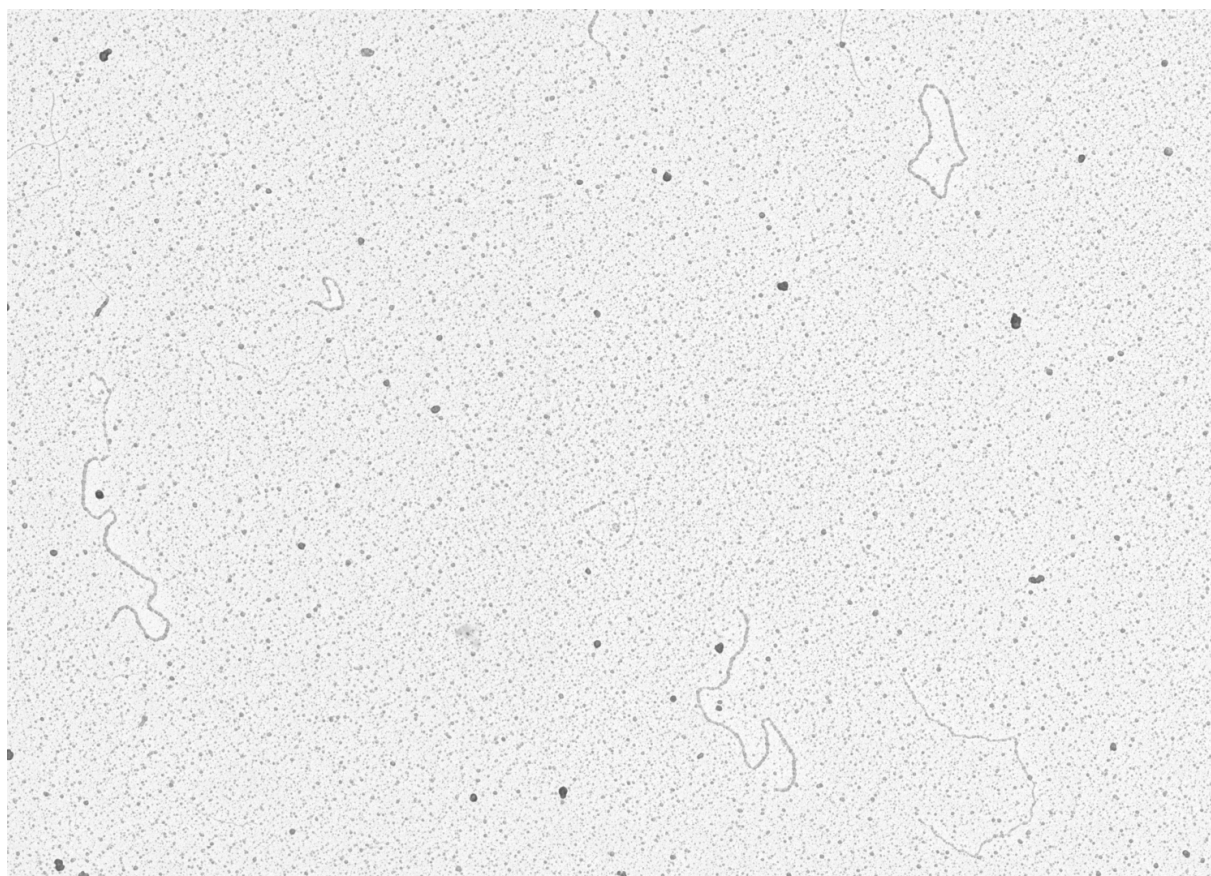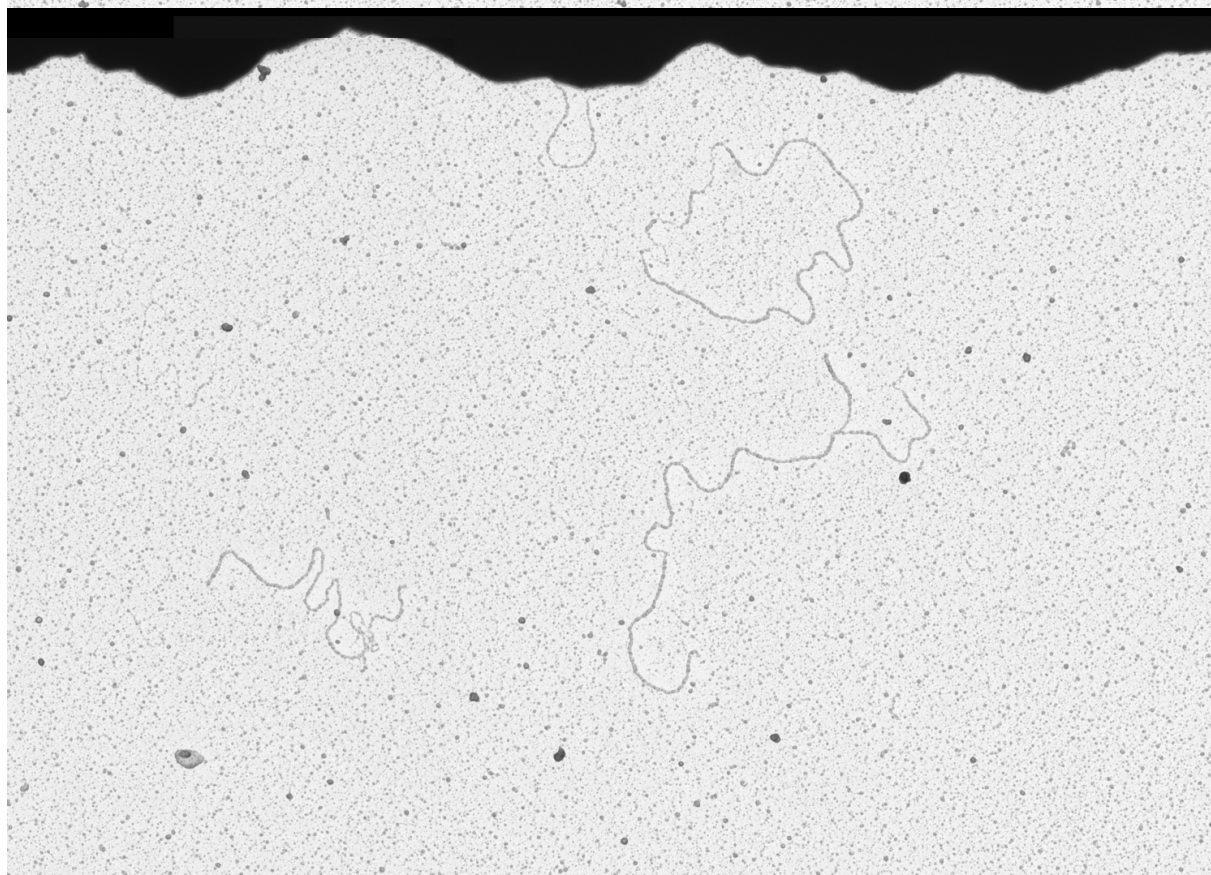

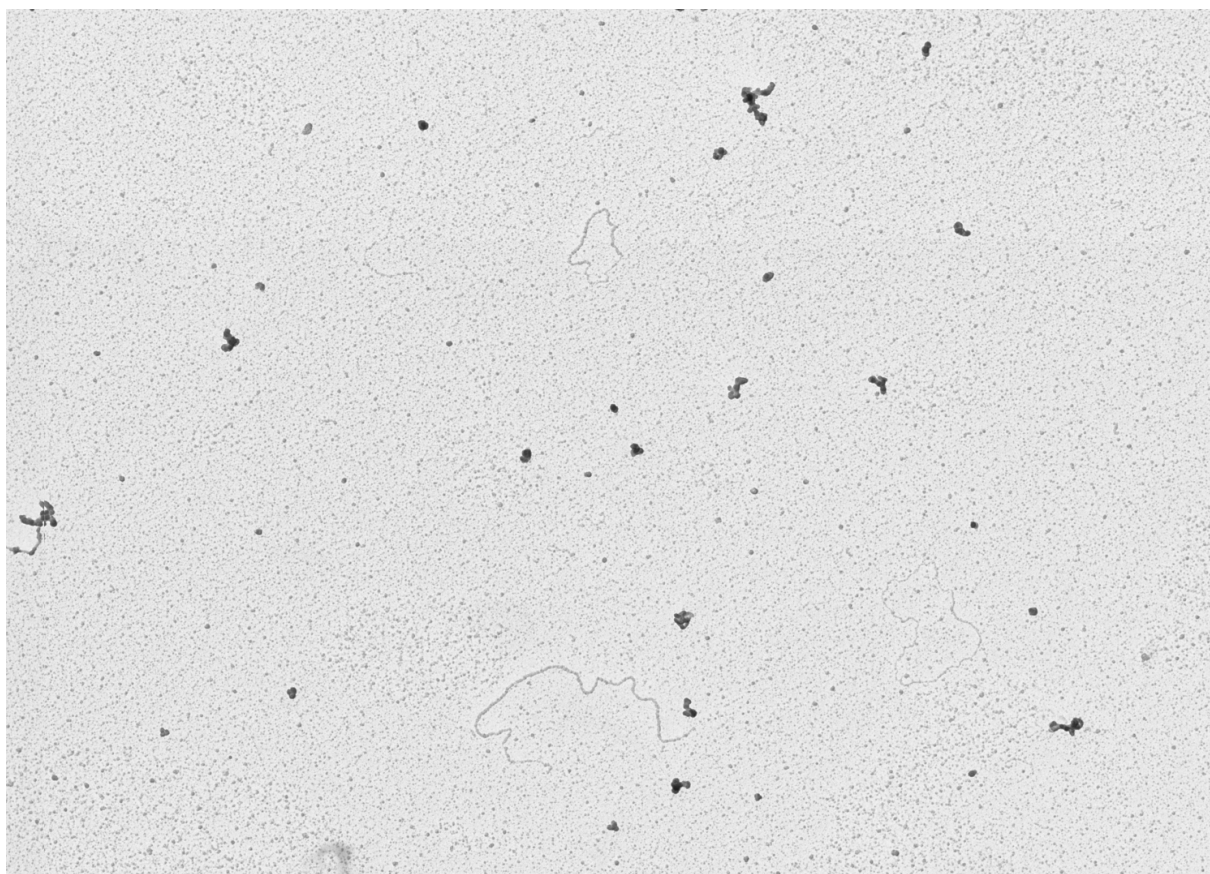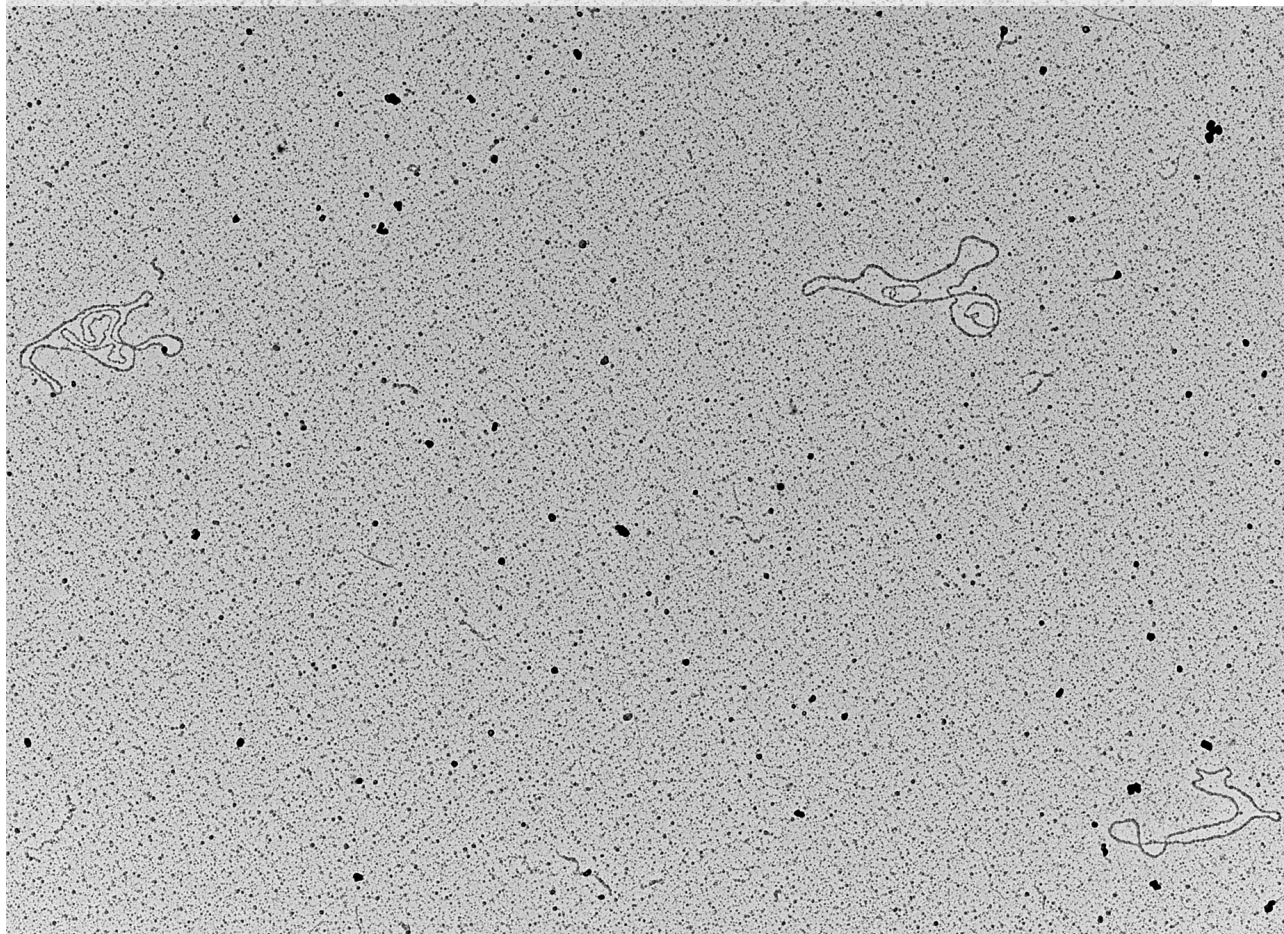

Figure 4d

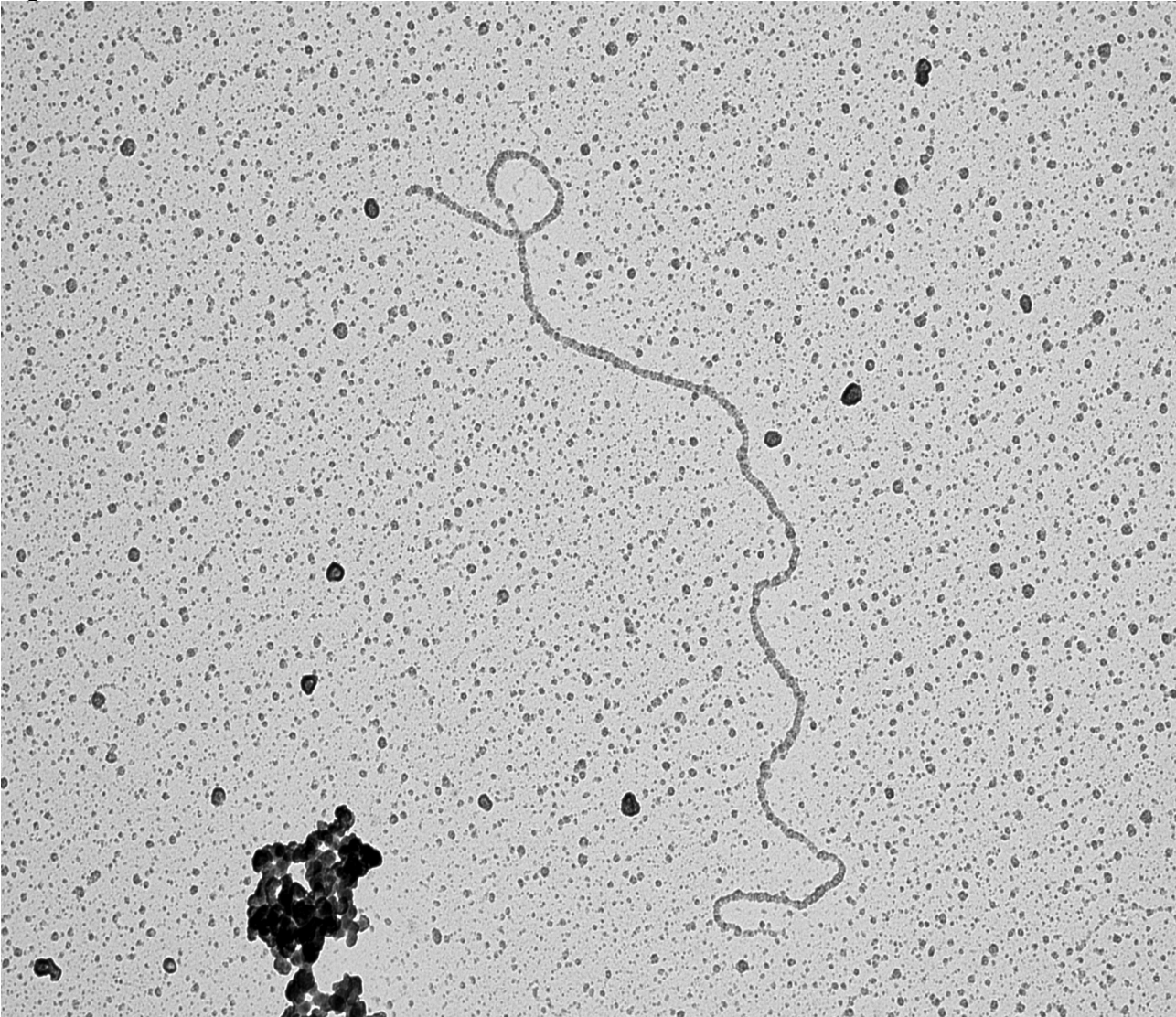

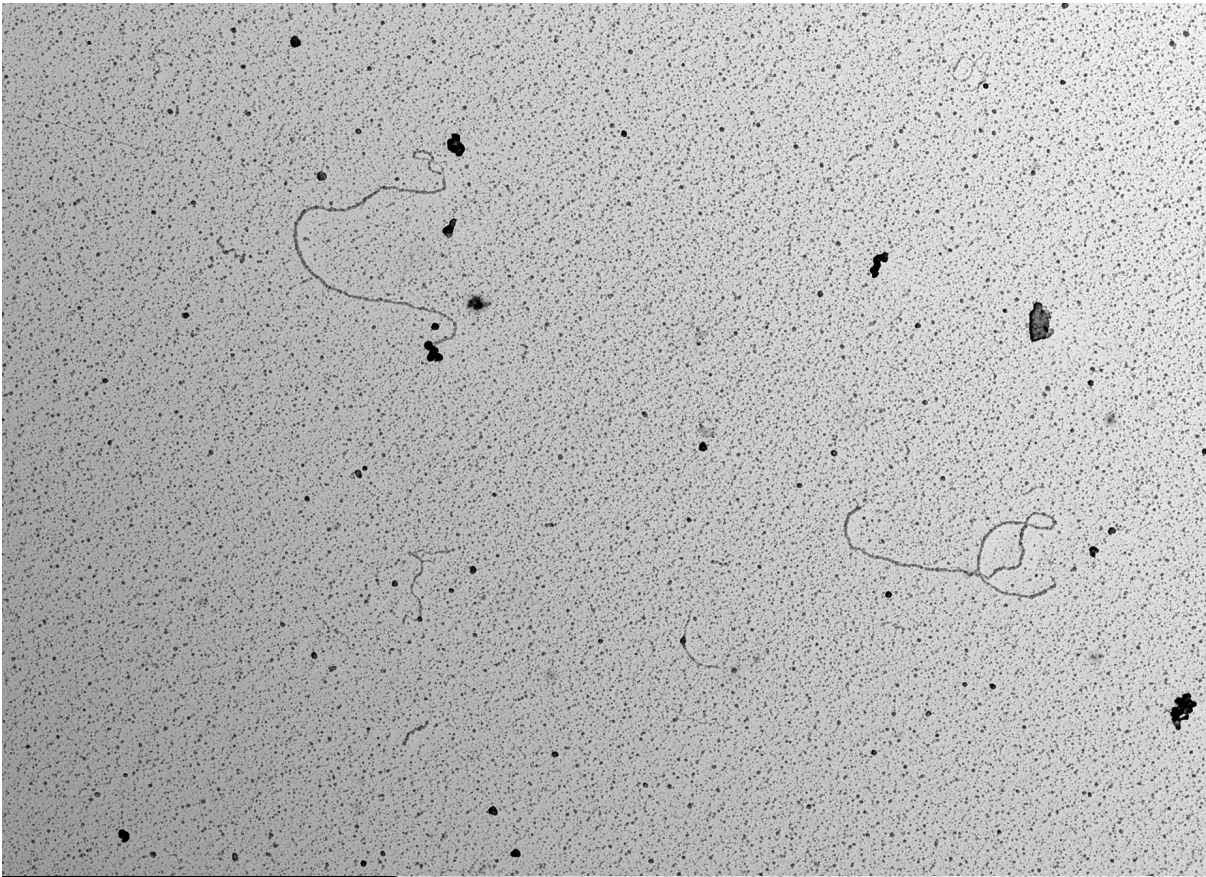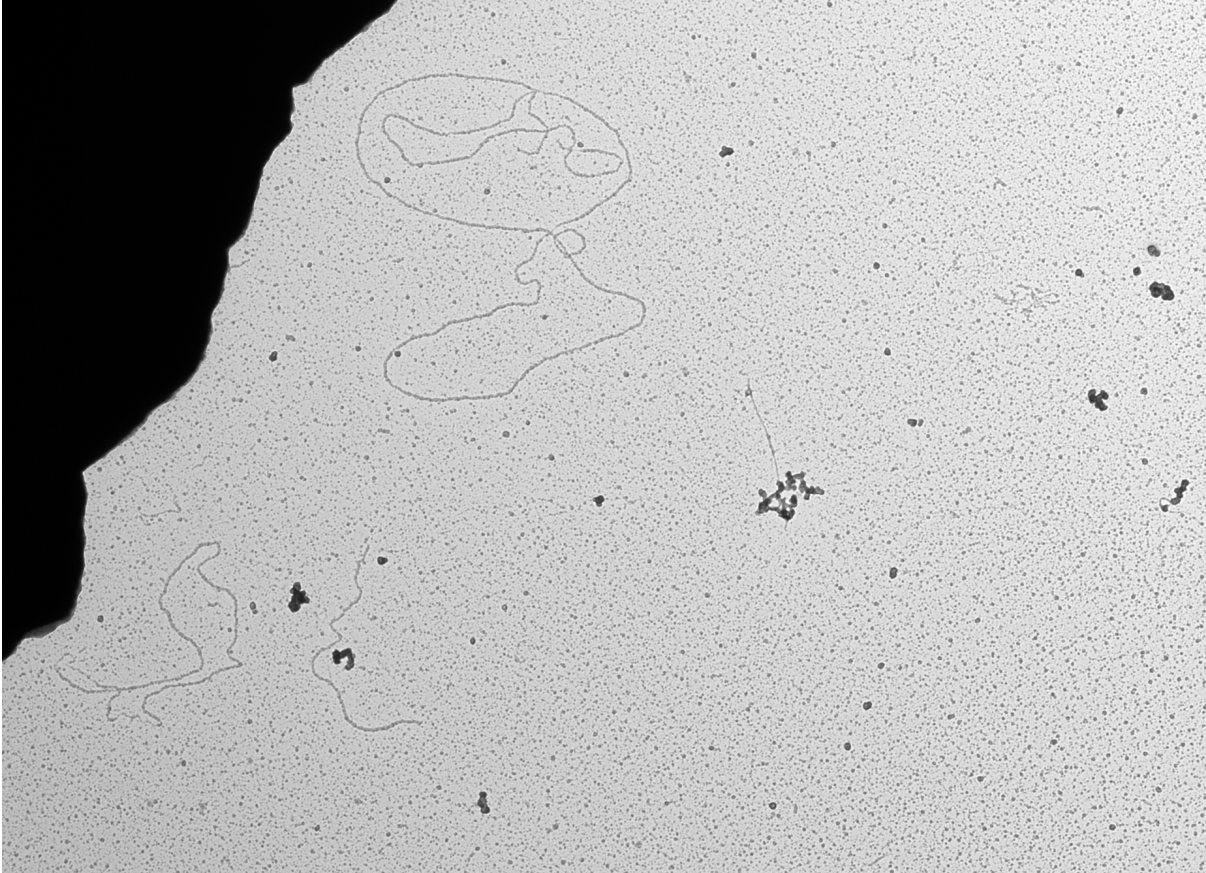

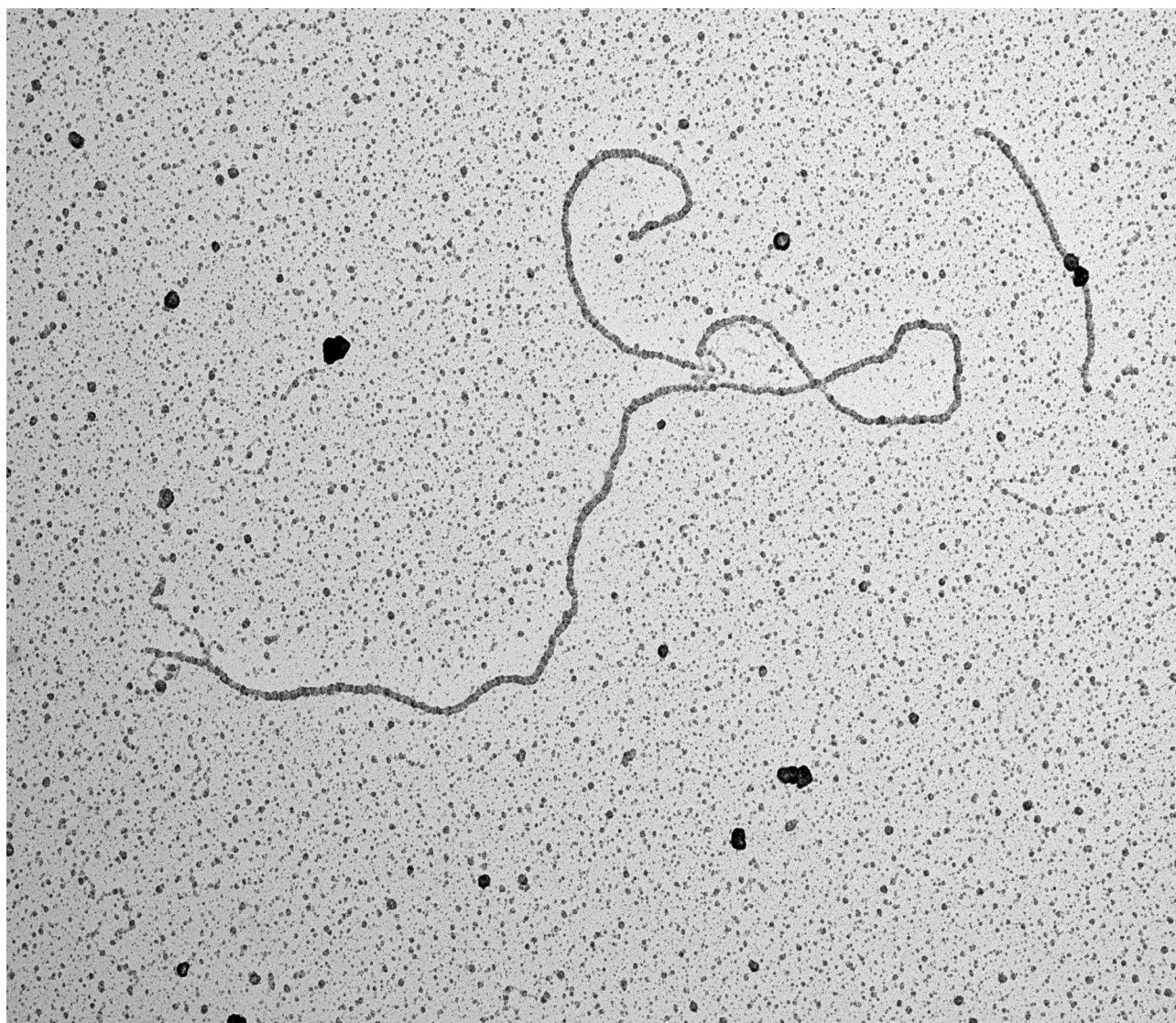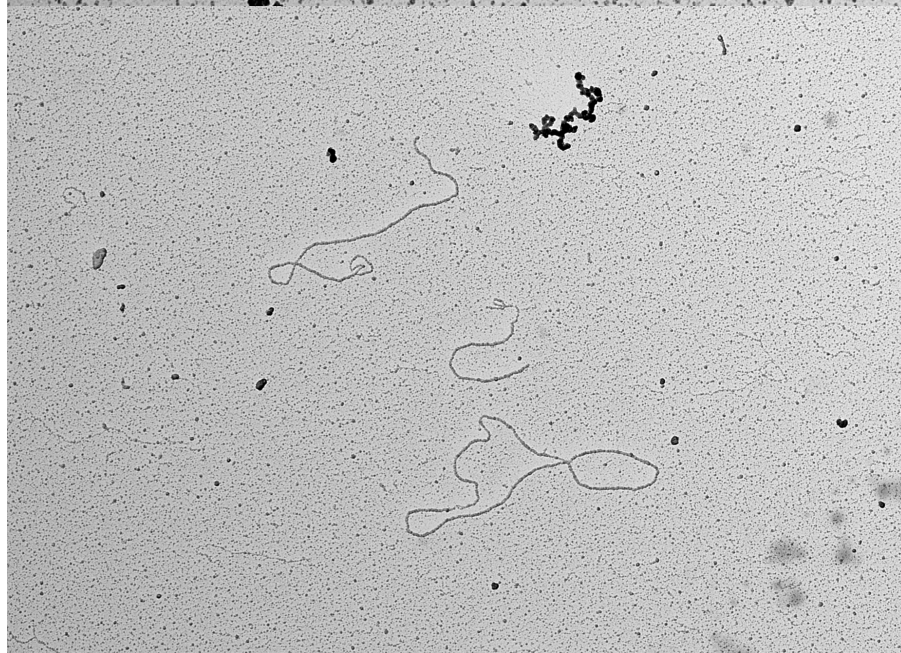

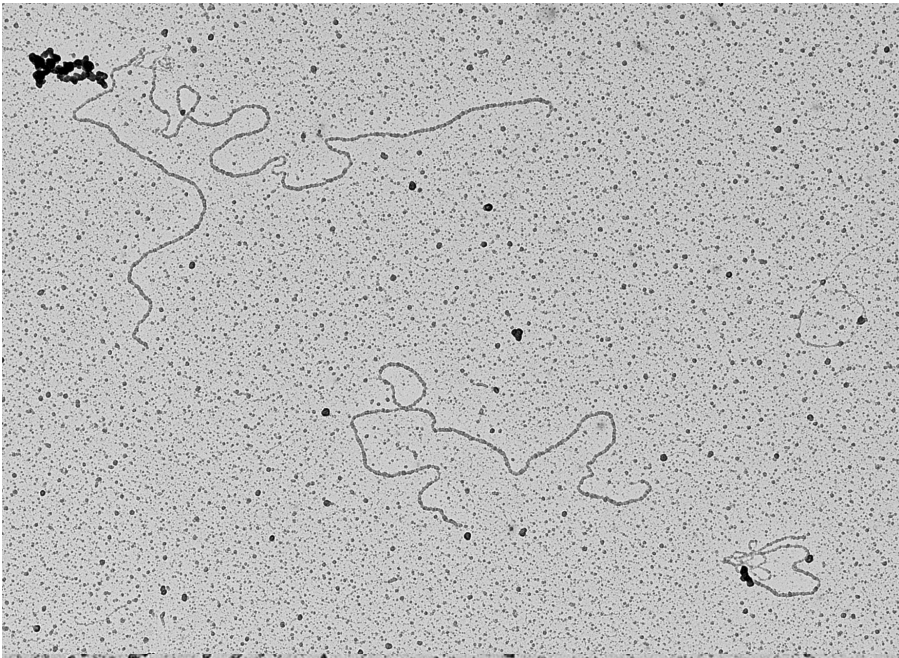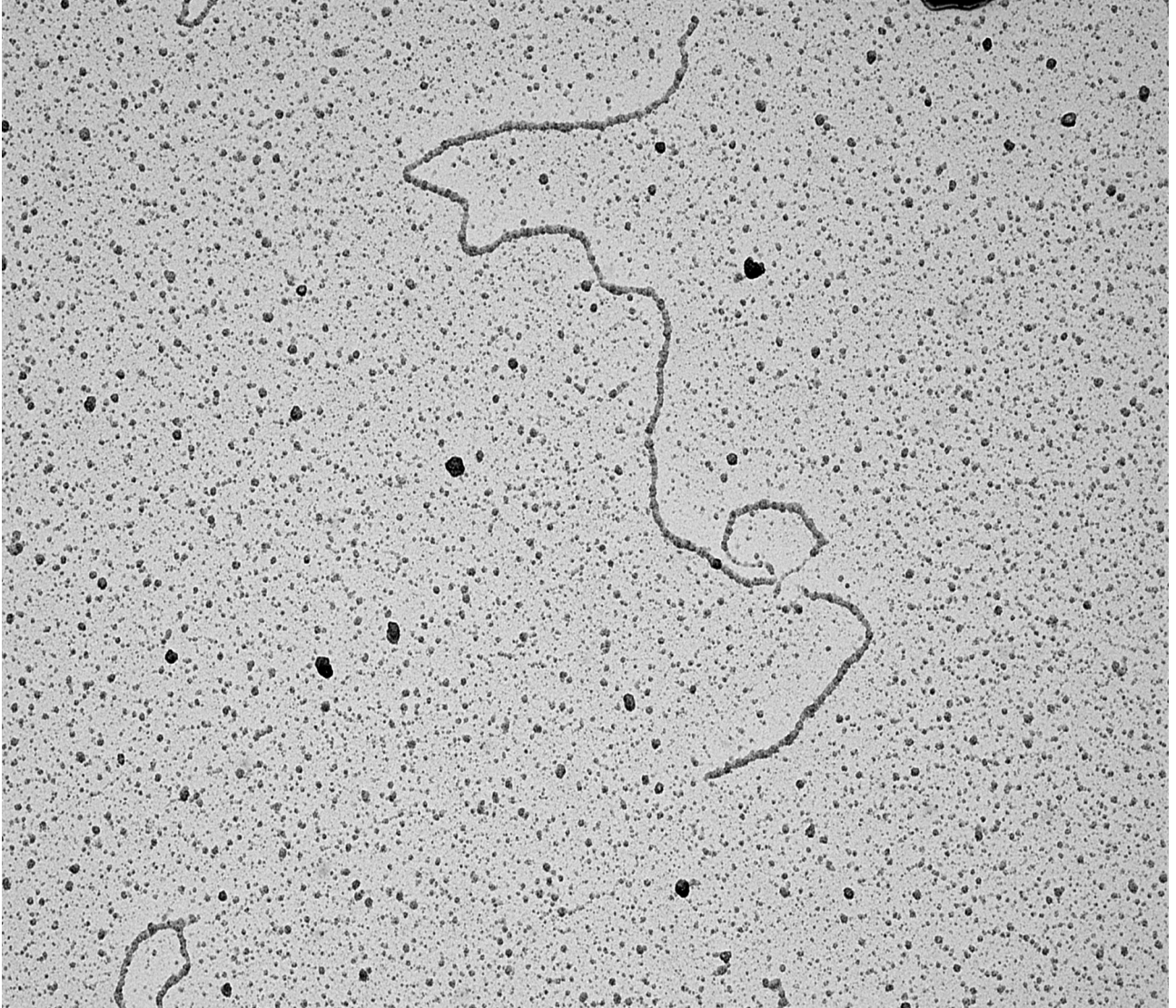

Supplement: Supplementary file 6 — Source Data [file 41467_2020_19139_MOESM6_ESM.zip › Source data 2nd rev/Source data Figure 4.pdf]

Figure 6A

DNaseI treatment in MEFs nuclei, telomeric probe

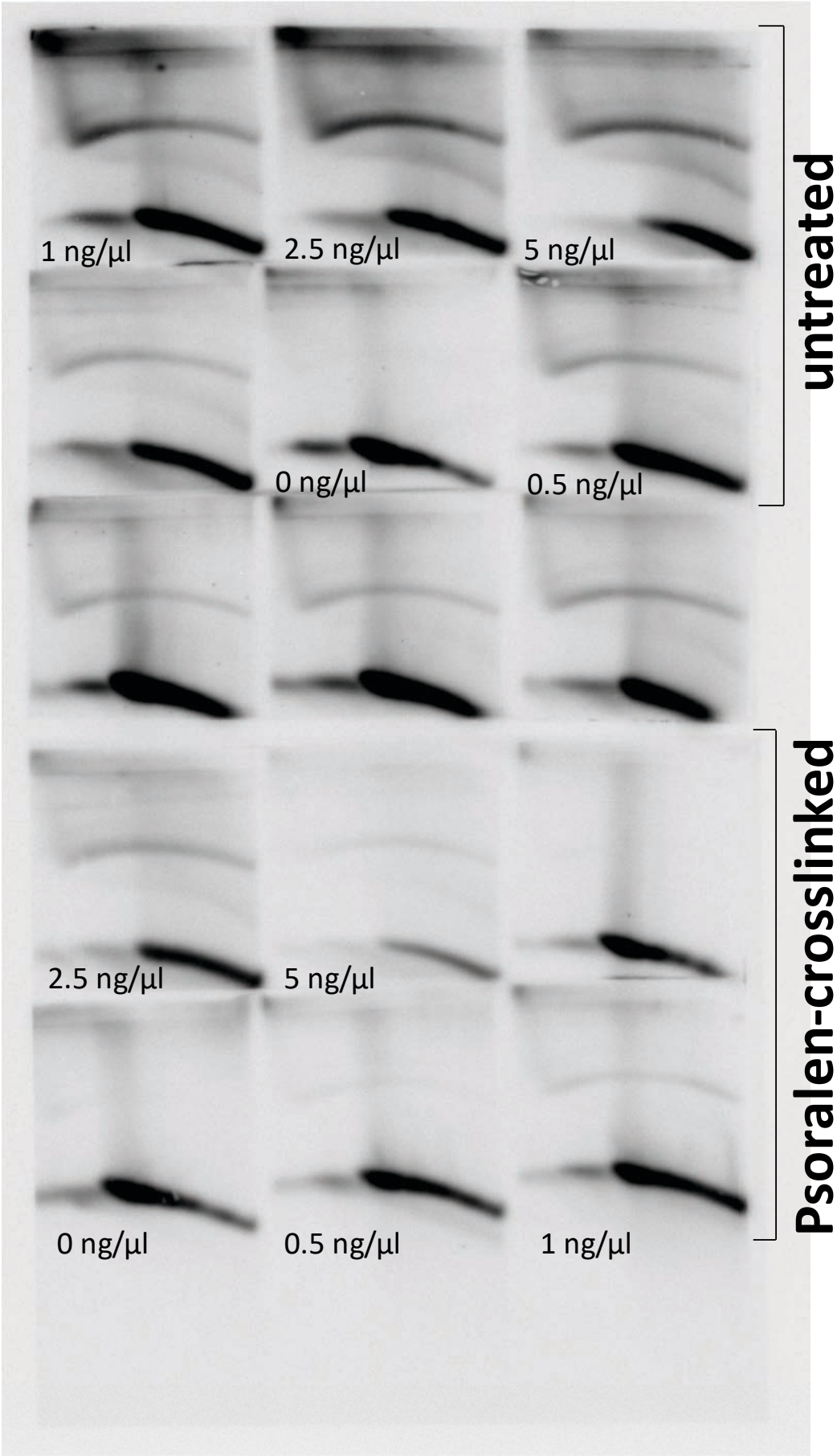

Figure 6B

U2OS cells

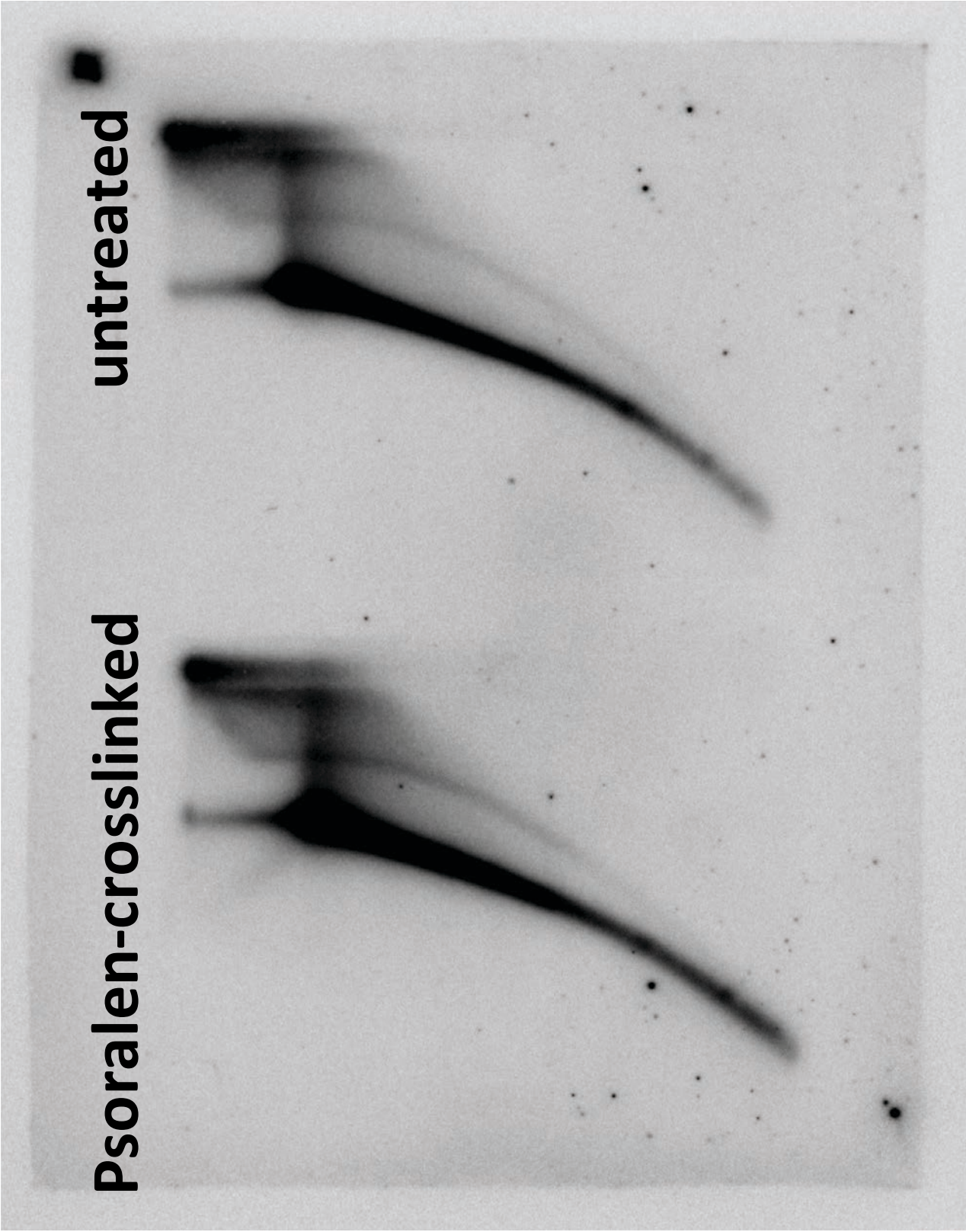

Supplement: Supplementary file 6 — Source Data [file 41467_2020_19139_MOESM6_ESM.zip › Source data 2nd rev/Source data Figure 6.pdf]

Figure S1A

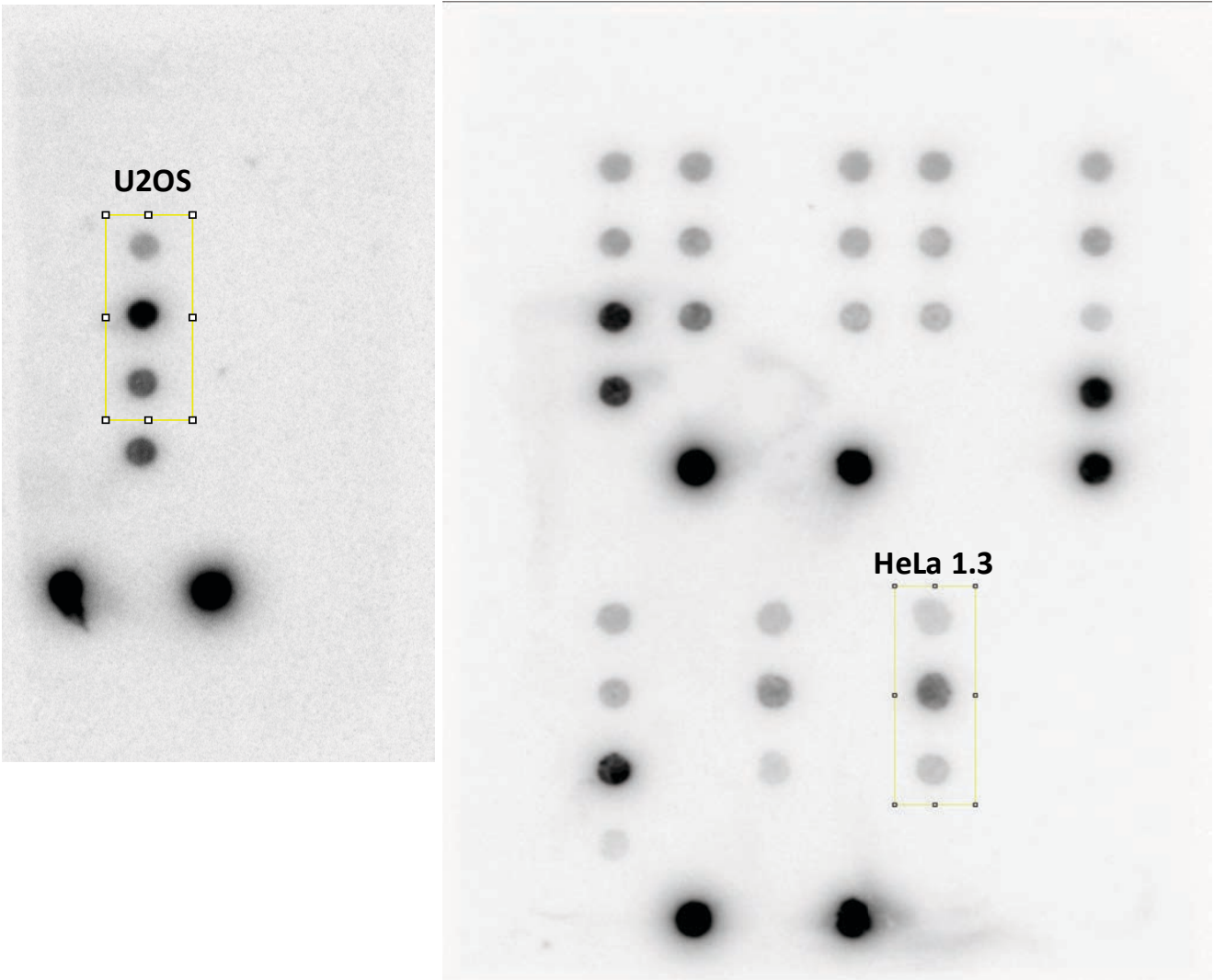

Supplement: Supplementary file 6 — Source Data [file 41467_2020_19139_MOESM6_ESM.zip › Source data 2nd rev/Source data Supplementary Figure 1.pdf]

Supplementary Figure 3b

| Loop size distribution (kb) |
| --- |
|  |
| 9.5000 |
| 6.3056 |
| 5.9444 |
| 4.8889 |
| 3.6944 |
| 3.6667 |
| 3.5556 |
| 2.7778 |
| 2.1667 |
| 2.0833 |
| 1.4444 |
| 1.3333 |
| 1.3333 |
| 1.2778 |
| 1.1111 |
| 1.0833 |
| 1.0278 |
| 0.9167 |
| 0.8889 |
| 0.7778 |
| 0.7778 |
| 0.6944 |
| 7.3056 |
| 4.3889 |
| 3.9167 |
| 2.9722 |
| 2.3889 |
| 1.8889 |

Supplementary Figure 3c


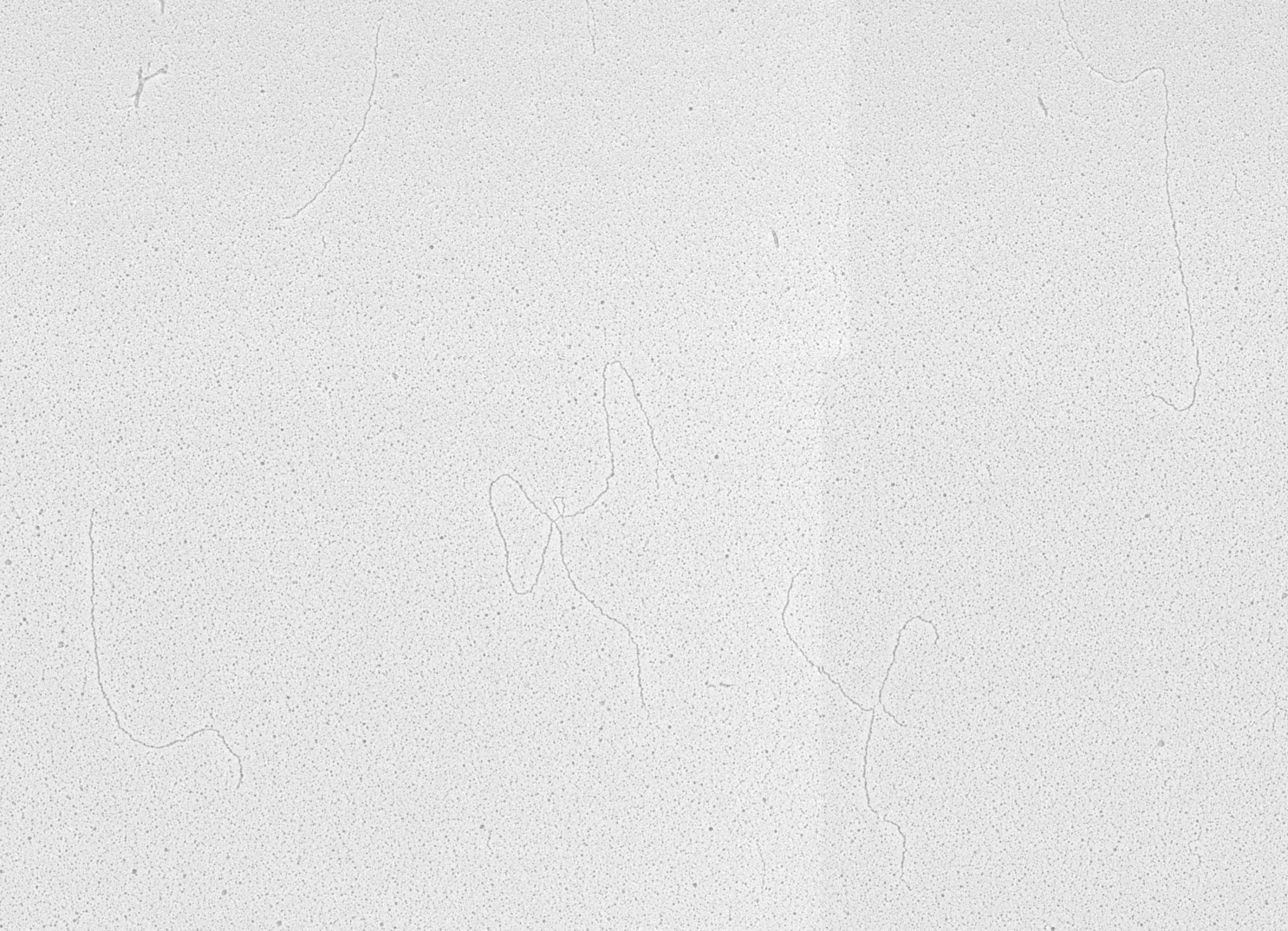

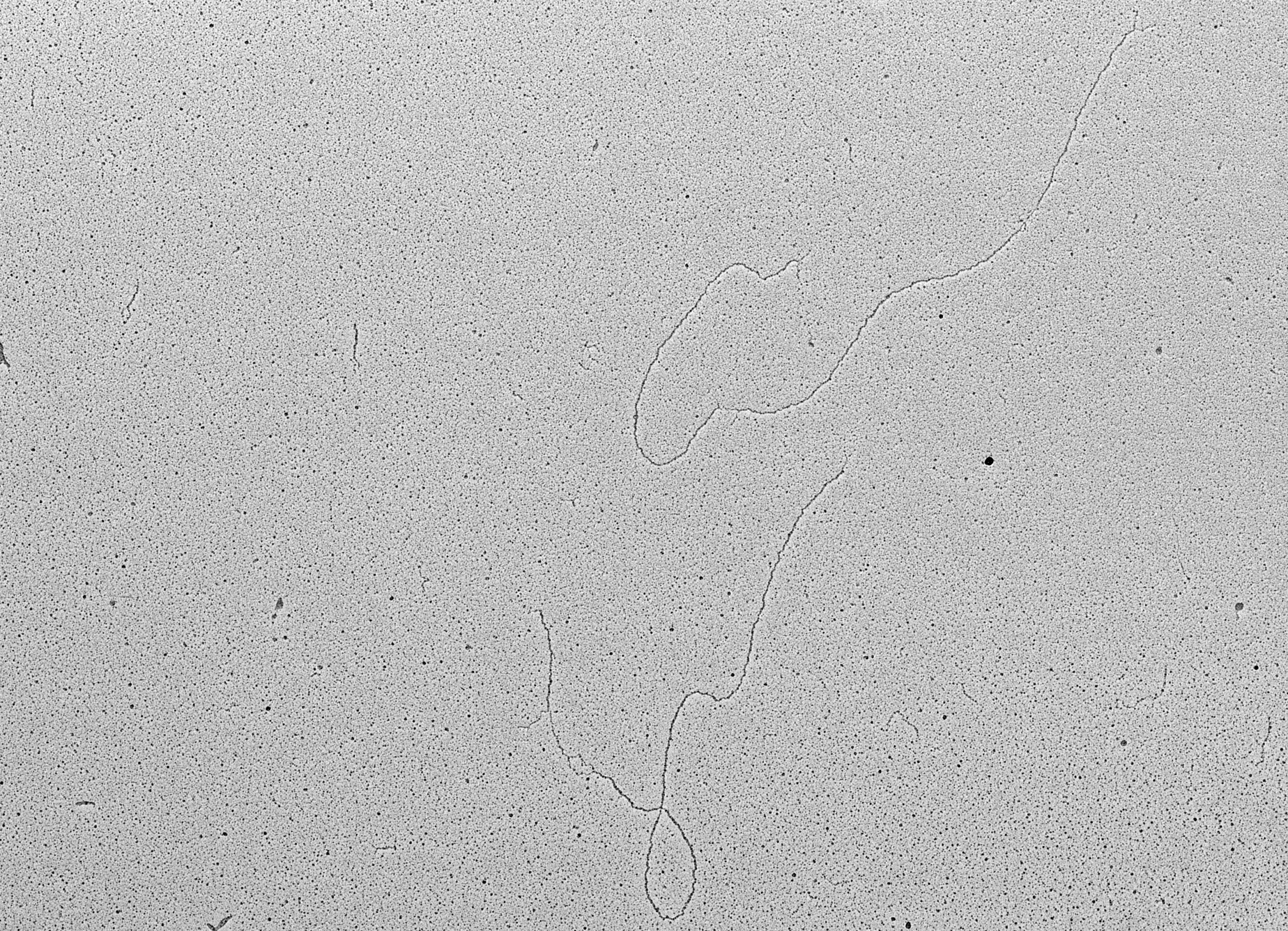

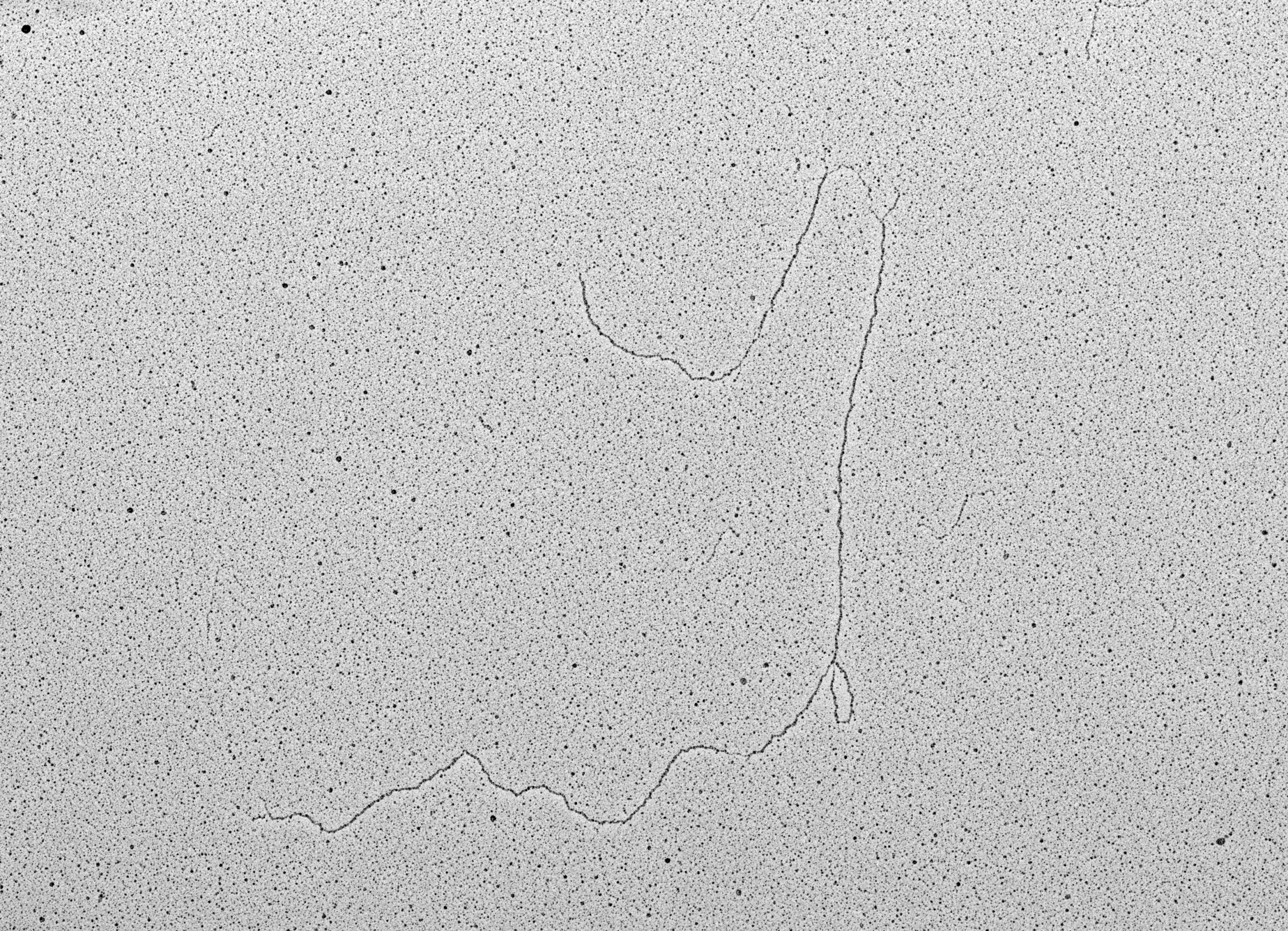

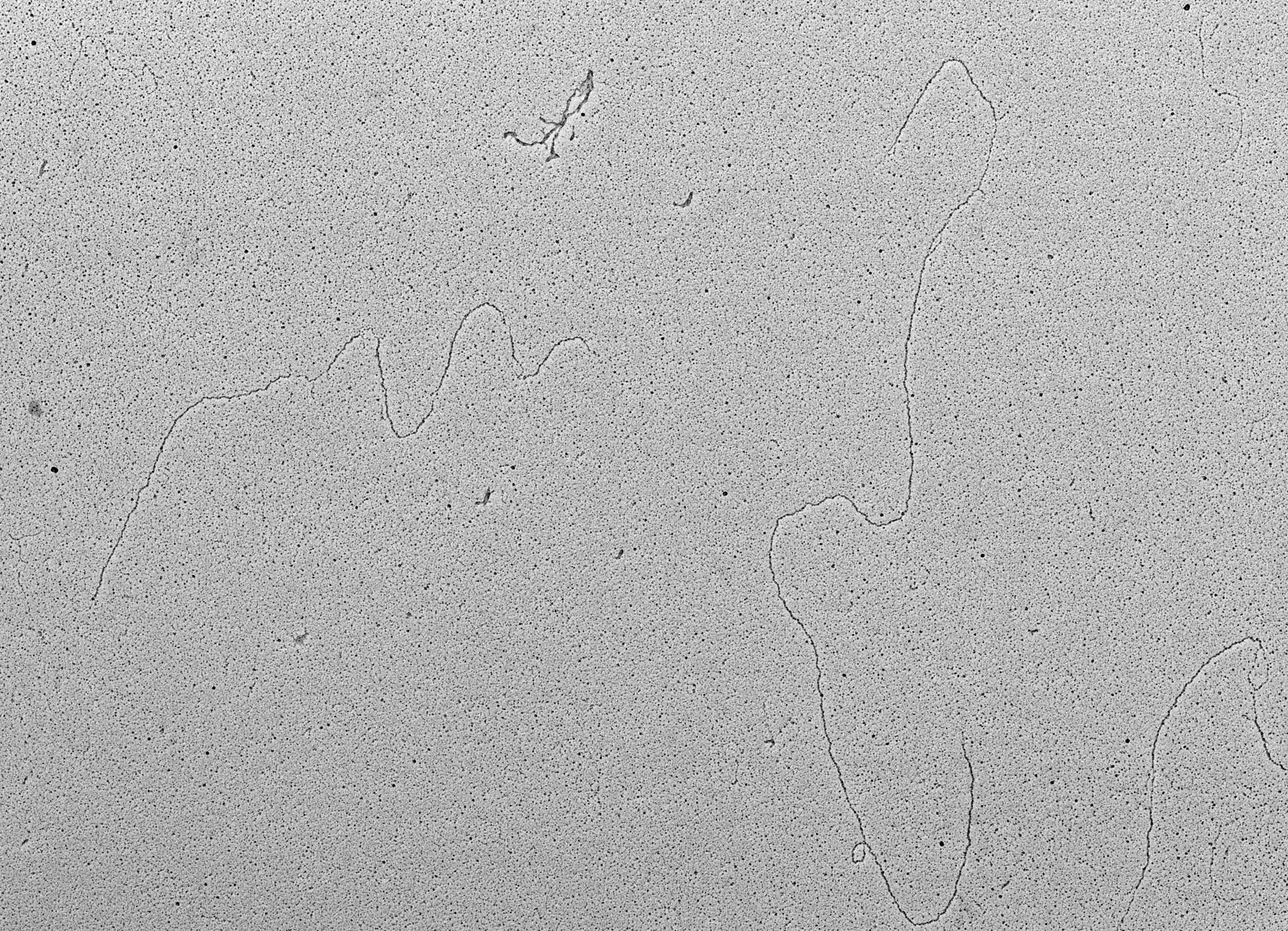

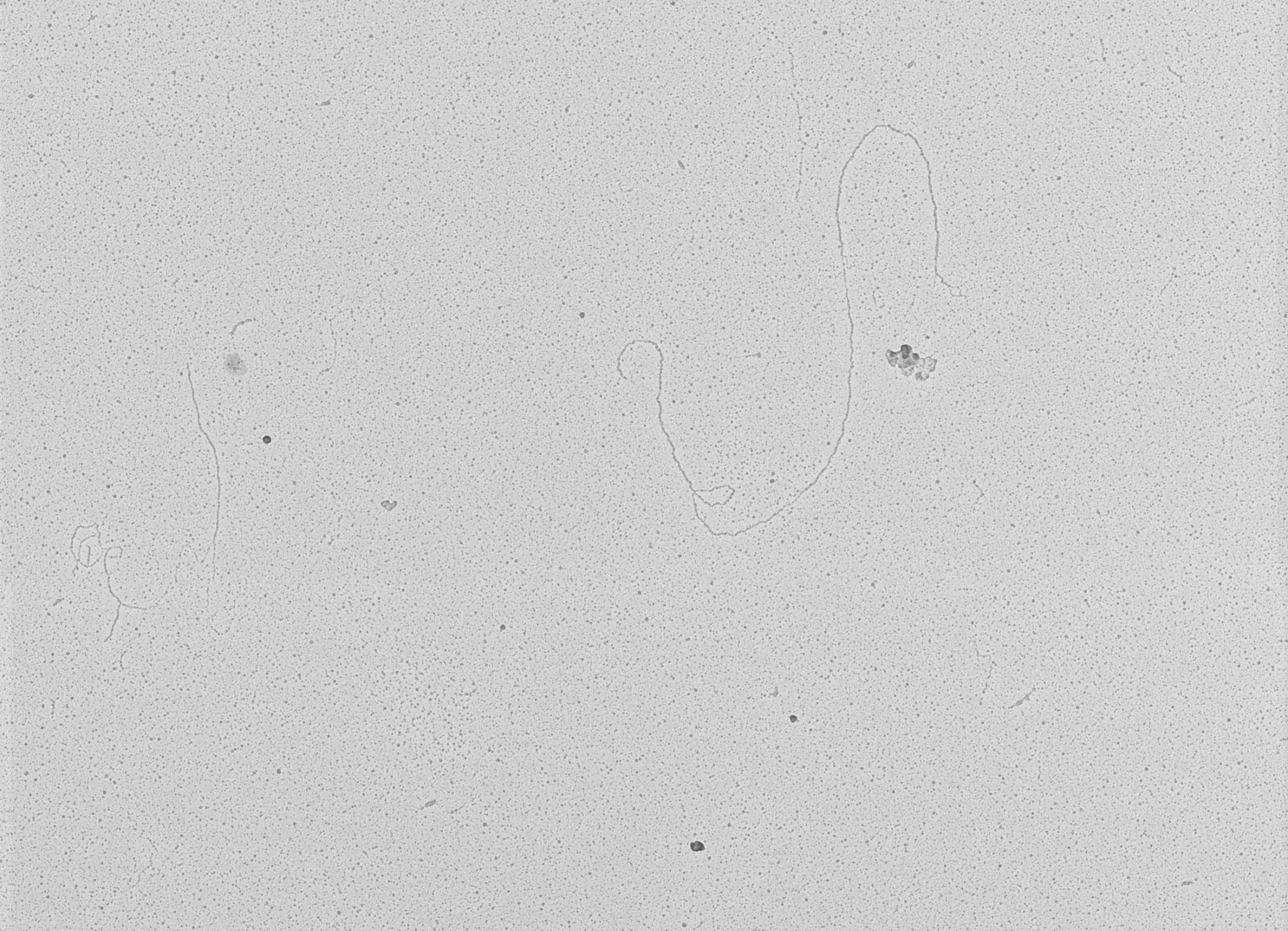

Supplement: Supplementary file 6 — Source Data [file 41467_2020_19139_MOESM6_ESM.zip › Source data 2nd rev/Source data Supplementary Figure 3.docx]

Figure S5A

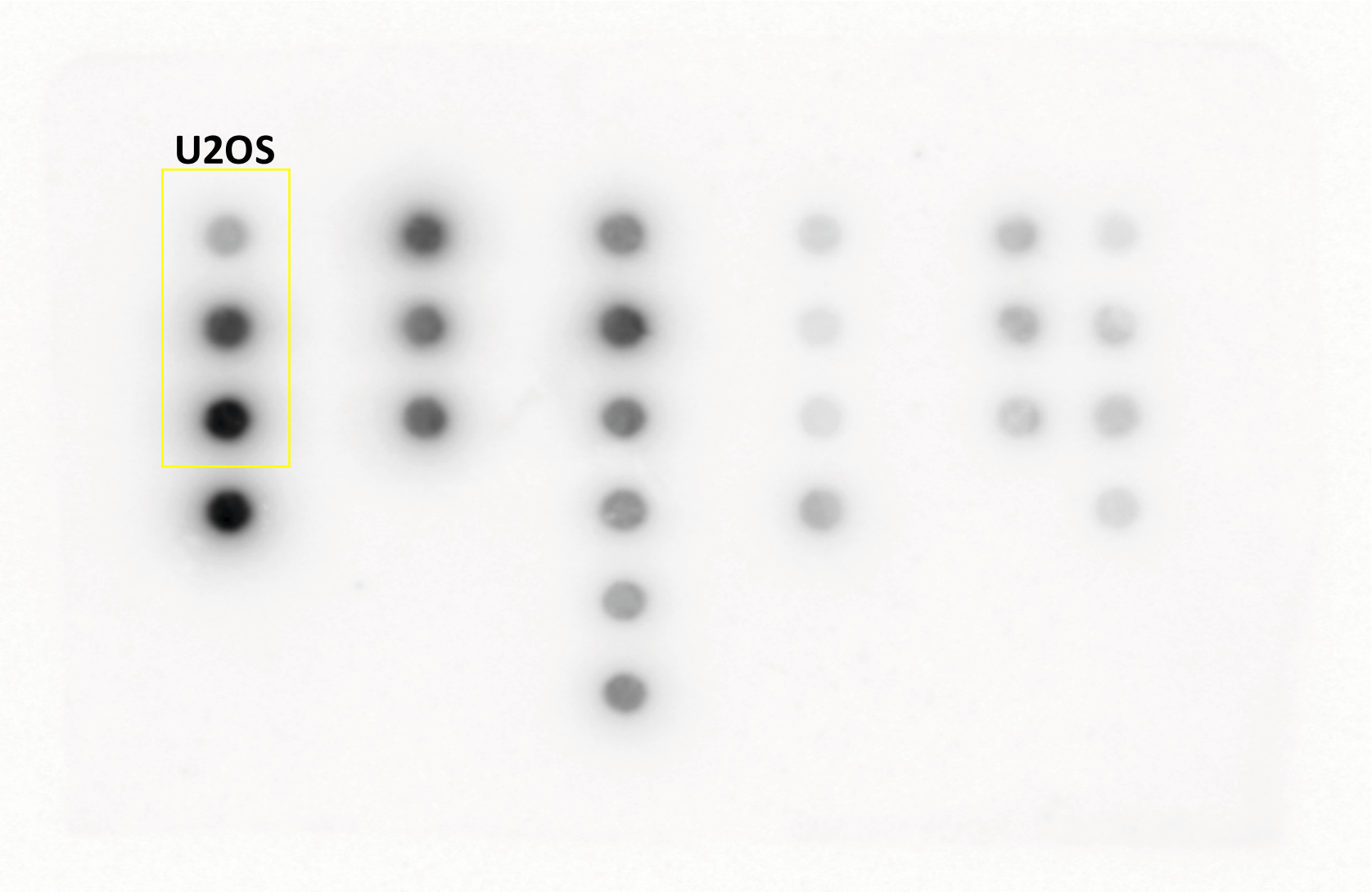

Supplement: Supplementary file 6 — Source Data [file 41467_2020_19139_MOESM6_ESM.zip › Source data 2nd rev/Source data Supplementary Figure 5.pdf]
